# Enzyme-Loaded Microcapsules as Intracellular Organelles for the Degradation of Nanoplastics by Cells

**DOI:** 10.1021/acsnano.5c15213

**Published:** 2025-12-16

**Authors:** Xin Liu, Jennifer Chow, Wenbo Wang, Robert Dierkes, Neus Feliu, Florian Schulz, Wolfgang R. Streit, Wolfgang J. Parak

**Affiliations:** † Fachbereich Physik, Center for Hybrid Nanostructures (CHyN), 14915 Universität Hamburg, Hamburg 22761, Germany; ‡ Department of Microbiology and Biotechnology, Universität Hamburg, Hamburg 22609, Germany; § Fraunhofer Center for Applied Nanotechnology (IAP-CAN), Hamburg 20146, Germany

**Keywords:** nanoplastics, intracellular degradation, endocytosis, enzymatic degradation, colloids

## Abstract

Increasing spills in the environment with plastic nanoparticles causes unwanted contamination. A proof-of-concept study is presented in which mammalian cells are loaded with enzymes capable of degrading capsules, here poly­(ethylene terephthalate) (PET) hydrolase. Loading into cells via endocytosis is achieved by polymeric encapsulation, which upon integration of poly­(ethylenimine) also provides suitable local working conditions for the enzymes. In this way, the enzymatic activity of the PET hydrolase is also maintained in the acidic environment of endosomes/lysosomes. It is demonstrated that PET nanoparticles endocytosed by cells can be degraded by cells upon exposure to encapsulated PET hydrolase enzymes. For this first, a colocalization analysis of endocytosed PET nanoparticles and encapsulated PET hydrolase is described, showing qualitatively that enzymes can encounter the plastics nanoparticles. Second, degradation of fluorescence-labeled plastics nanoparticles via enzymatic degradation is monitored in terms of loss of intracellular fluorescence over time. Limitations and potential future applications perspectives of this concept are discussed. A roadmap is presented on how this semiquantitative study could be extended into obtaining quantitative data and first applications.

## Graphical abstract

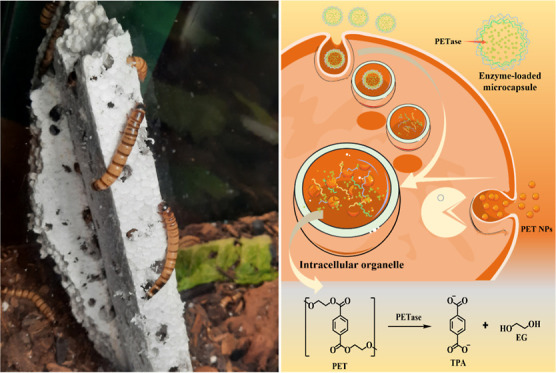

## Introduction

There is an ongoing concern about pollution of the environment with nano- and microplastics.^1,2^ Despite the fact that plastics is one of the most used materials, there are unsolved issues with its recycling and as a matter of fact, there is an increasing mass of waste plastics, including nano- and microplastics as degradation product.^3,4^ It is known that plastics-based particles may have negative long-term effects on the health of animals and humans.^5–7^ In the last years, a large number of research articles, reviews, and comments were published dealing with this topic.^8–10^ Toxicity has been demonstrated at different levels, from effects observed with in vitro models,^11^ unicellular eukaryotes,^12^ crop growth,^13^ marine organisms,^14^ the central nervous system,^15^ and male reproduction.^16^ Nano- and microplastics originate as degradation products of plastics-based industrial and consumer products, whereby toxicity may not only originate from the plastics but in addition also by released integrated additives.^17^ Nano- and microplastics also interact with the environment, for example, by adsorption of molecules. Such adsorption may influence biodistribution. In case of adsorbed heavy metal ions, nano- and microplastics may act as vehicles facilitating cellular entry of these ions via exocytosis.^18^ Also, adsorption of proteins, leading to the formation of a protein corona, influences the biodistribution and fate of nano- and microplastics in organisms.^19–22^

Spread and toxicity of nano- and microplastics in the environment consequently demand solutions on how they could be removed. Apart from technical solutions,^23,24^ there might be also a biological route: Some worms and bacteria are, for example, known for being able to digest plastics,^25–29^ see also the table of content (TOC) figure (including a photo of superworms feeding on styrofoam; photo WJP taken at Audubon Nature Institute, Butterfly Garden & Insectarium, New Orleans, LA, US). On the biomolecular level, this is facilitated by enzymes that are able to degrade different types of plastics.^30,31^ This suggests that one could downscale the plastics-eating worm to the cellular level, which would offer several different potential application routes for plastics degradation, as will be outlined in the discussion.

Nano- and microplastics nanoparticles (NPs) are endocytosed by eukaryotic cells,^32,33^ similar to most other types of NPs, locating them inside intracellular vesicles, i.e., endosomes/lysosomes. Thus, having plastic-degrading enzymes as “intracellular organelles” located in endosomes/lysosomes could allow for intracellular degradation of endocytosed plastic NPs. There are however several challenges toward the implementation of such concept. First, free enzymes are not endocytosed to a high extend by cells and thus it is not efficient to deliver them to endosomes/lysosomes.^34,35^ This has been, however, demonstrated with encapsulated enzymes toward the context of enzyme replacement therapy.^36^ Second, the operation range of enzymes often is limited to a certain pH window, and enzymes operational at neutral pH may not work in the acidic environment of endosomes/lysosomes.^37,38^ Third, when enzymes are delivered to endosomes/lysosomes, it is not certain whether they are in the same endosomes/lysosomes as the endocytosed plastic NPs.^39,40^ Fourth, when the enzymes finally degrade the plastic NPs, it needs to be warranted that the degradation products are nontoxic and can be efficiently exocytosed. In the following, concepts to tackle the first three technical challenges will be presented, and perspectives toward the fourth are being discussed. At the end, a “roadmap” is presented what would be needed to turn such proof-of-concept in a practical application.

## Results and Discussion

In our work, we used the *Ideonella sakaiensis*-derived PET hydrolase (IsPETase, hereafter referred to as PETase),^28^ an enzyme known to degrade polyethylene terephthalate (PET) NPs. While PET NPs are a specific type of nanoplastics, enzymes which can hydrolyze other nanoplastics materials are known.^30,31,41^ Recombinant IsPetase (here termed as PETase) was produced in *E. coli* BL21 and extracted using a His-tagged protein, and PET NPs were produced according to published methods (see the Supporting Information for details),^42,43^ cf. Figure S1. For visualization purpose, also PET NPs, which integrated rhodamine B (PET-RB NPs), were produced, which had a diameter of around *d*_c_ = 138 ± 19 nm, see Figure 1a. The physicochemical properties of PET and PET-RB NPs were characterized. Also, their fluorescence as their colloidal properties in salt- and protein-containing solutions was found to be stable over time (see Figures S2–S8 for characterization data).

**Figure 1:**
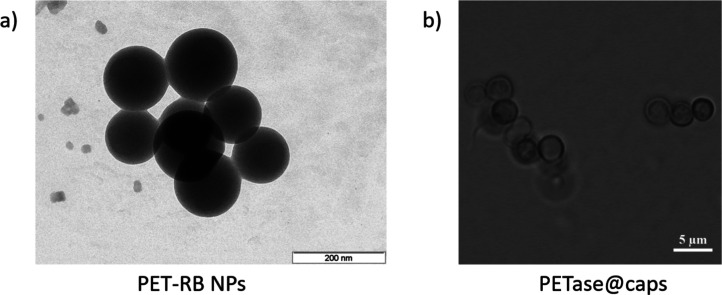
(a) Transmission electron microscopy (TEM) image of PET-RB NPs. The scale bar indicates 200 nm. (b) Optical microscopy image of PETase@caps. The scale bar indicates 5 μm.

Degradation of PET by PETase was monitored by the presence of fluorescent 2-hydroxyterephthalate (HOTP), which is formed by the Fenton reaction between the hydrolysis product of PET (terephthalic acid (TPA)) and FeSO_4_ + H_2_O_2_, see Figures 2a and S9–S12. PETase, however, operates best under neutral or slightly alkaline conditions, whereas there is only insufficient hydrolysis of PET at a low pH (pH = 4), see Figure 2b. This would exclude the working of PETase inside highly acidic endosomes/lysosomes. However, for working as an intracellular organelle, PETase also needs to be present in cells. Proteins can be delivered to cells via endocytosis, which leaves them in acidic intracellular compartments, i.e., endosomes/lysosomes. Operation of PETase under such acidic conditions thus is required.

**Figure 2:**
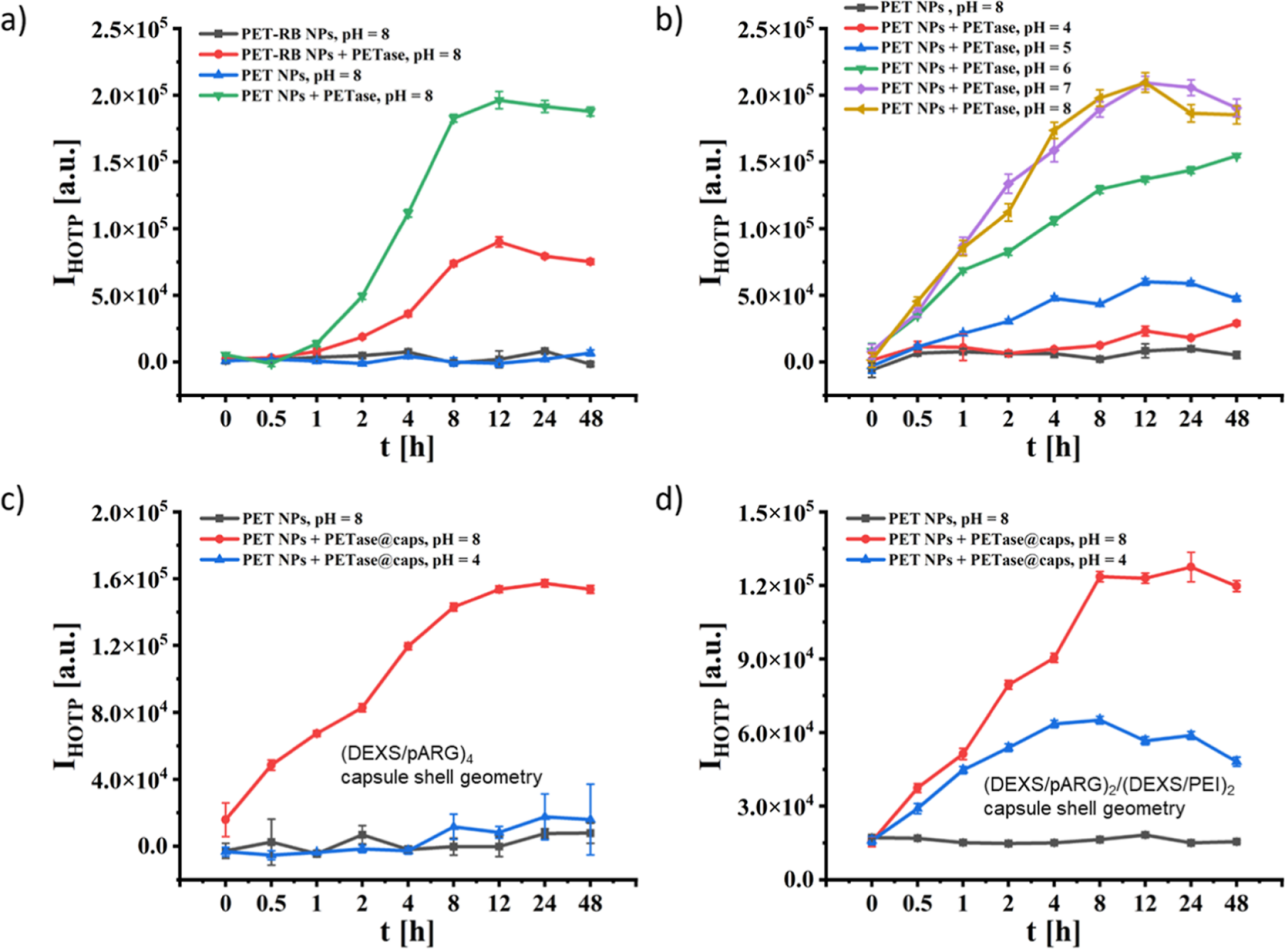
Fluorescence-based assay to monitor the hydrolysis of PET by PETase upon the incubation time *t*, based on the detection of the fluorescence intensity I_HOTP_ (excitation and emission recorded at λ_ex_ = 326 nm, λ_em_ = 438 nm) of HOTP. (a) Incubation of PET and PET-RB NPs with or without the presence of PETase in phosphate buffer (PB) at pH = 8. (b) Incubation of PET NPs under the presence of PETase in PB at different pH values. (c) Incubation of PET NPs under the presence of PETase@caps with (DEXS/pARG)_4_ capsule shell geometry (which had been disrupted prior to the assay by ultrasound sonication) in phosphate buffer (PB) at pH = 8 or 4. (d) Incubation of PET NPs under the presence of previously sonicated PETase@caps with (DEXS/pARG)_2_/(DEXS/PEI)_2_ capsule shell geometry in PB at pH = 8 or 4. Note that the absolute values of (a,b) cannot be directly compared with the ones of (c,d), as the absolute amount of PETase in the capsules could be only estimated (cf. Figures S12 and S13).

While proteins can be endocytosed,^44^ efficiency is rather low. On the other hand, by encapsulation and thus formation of nano- or microparticles, proteins can be well delivered to endosomes/lysosomes of cells via endocytosis.^45^ Upon using biodegradable polymers as an encapsulation matrix, the encapsulated proteins can be released in the endosomes/lysosomes.^46,47^

In our work, we used polyelectrolyte layers made by alternating deposition of negatively and positively charged biodegradable polymers,^48,49^ dextran sulfate sodium salt (DEXS, *M*_w_ ≈10 kDa) and poly-l-arginine hydrochloride (pARG, *M*_w_ ≈15–70 kDa), respectively, to encapsulate PETase. This led to PETase@caps particles with the shell geometry (DEXS/pARG)_4_ with a diameter of around *d*_c_ ≈ 3.3 μm. These capsules degrade after having been endocytosed.^50^

For a first test, whether enzymatic activity is preserved after encapsulation and degradation, PETase@caps was sonicated to disrupt the capsules and thus allow access of PET NPs to the PETase. The enzymatic activity was then demonstrated with the same assay as used for free PETase at pH = 4 or 8, see Figure 2c. Encapsulation thus largely preserved enzymatic activity at pH = 8.

In order to solve the problem of unfavorable pH conditions inside endosomes/lysosomes, a buffer was included in the shells of the capsules. This would buffer the protons inside endosomes/lysosomes and thus provide less acidic local conditions around the PETase. For this, capsules were made in which two layers of pARG were substituted by branched poly­(ethylenimine) (PEI, *M*_w_ ≈25 kDa), which is known to buffer pH, leading to the shell geometry (DEXS/pARG)_2_/(DEXS/PEI)_2_, see Figure 1b.^51^ In a previous study, it was shown by incorporation of a pH-responsive fluorophore that the presence of PEI in fact buffered the pH inside the lysosomes where the capsules were residing, i.e., the pH was less acidic.^51^ Presence of PEI on the other hand reduced cell viability and thus only a limited amount of few internalized capsules per cell is possible in order to avoid harmful effects.^51^

Presence of PEI allows for (some) enzymatic activity also in a globally acidic environment: In Figures 2d and S13, it is shown that in the PEI-containing shell configuration, PETase@caps also operates at pH = 4. Thus, by encapsulation, PETase can be effectively delivered to endosomes/lysosomes of cells, and due to the added proton buffer, it retains activity in this acidic compartment. In the following, “@caps” always refers to encapsulation with PEI-containing shells. Of note, for reasons discussed at the end of the articles, the tests of enzymatic activity as carried out here are qualitative and not quantitative. This is in particular due to the reason that the HOTP assay is only used “in test tube” but not for the in vitro experiments. For this reason, details, such as the linear range and the limit of detection of this assay, were not determined in our study.

Despite their micrometer size, capsules as the ones reported here (i.e., PETase@caps) are endocytosed.^52^ It has to be noted that “endocytosis” is an umbrella expression for different pathways,^53^ all leading to intracellular delivery in vesicular structures (endosomes/lysosomes). In the case of capsules, this has been confirmed with pH-responsive indicators showing the location in acidic vesicles,^54^ with electron microscopy and fluorescence microscopy showing the membrane of the surrounding vesicles,^55^ with blockers for specific pathways,^55^ and with colocalization studies of endocytosis markers.^55^ Lipid-raft-mediated macropinocytosis plays an important role in the endocytosis of such micrometer-sized capsules.^55^ Due to their different physicochemical properties (here much smaller size), the PET NPs likely will be endocytosed via a different endocytosis pathway and thus are not automatically colocated with the capsules.^39,40^

While PETase@caps and PET NPs are therefore assumed to be endocytosed, it still needs to be proven that they can be present in the same intracellular compartments. For this, bovine serum albumin (BSA) with a fluorescein isothiocyanate (FITC) label was encapsulated (BSA-FITC@caps) and used as fluorescent capsules to emulate the nonfluorescent PETase@caps. Human cervical carcinoma (HeLa) cells were first exposed to PET-RB NPs under serum supplemented culture conditions, followed by addition of BSA-FITC@caps the next day and after another overnight exposure staining of endosomes/lysosomes by LysoTracker. Exposure of Hela cells to both, PET-RB NPs and BSA-FITC@caps, was done under nontoxic conditions (Figures S14 and S15; for more information about the toxicity of capsules, we refer to the literature^56,57^ and the discussion in the Supporting Information). The colocalization of PET-RB NPs and BSA-FITC@caps was probed by confocal microscopy in the FITC and RB channels; see Figure 3. First, the controls shown in Figure 3, where not all components were added, show that there is no bleed-through between the different channels of fluorescence. Second, as reported before,^58^ the PEI in the shell of the capsules transiently opens the membrane of the endosomes/lysosomes where the capsules are located, followed by a partial release of BSA-FITC to the cytosol. This can be seen by the fluorescence spread from the capsules to the volume of cells in the FITC channel. The images indicate that at least part of endocytosed PET NPs are colocalized with FITC-BSA, but the images do not allow for quantitative conclusions, for example, whether colocalization happens inside endosomes/lysosomes or in the cytosol. Due to the limited amount of data of only a few images of cells, no quantitative analysis (such as, for example, quantification via Manders coefficients) was performed, in particular, as in the roadmap given at the end of this report, it is pointed out that for future studies, a different enzyme/capsule system should be used. Thus, no statistical analysis is provided.

**Figure 3:**
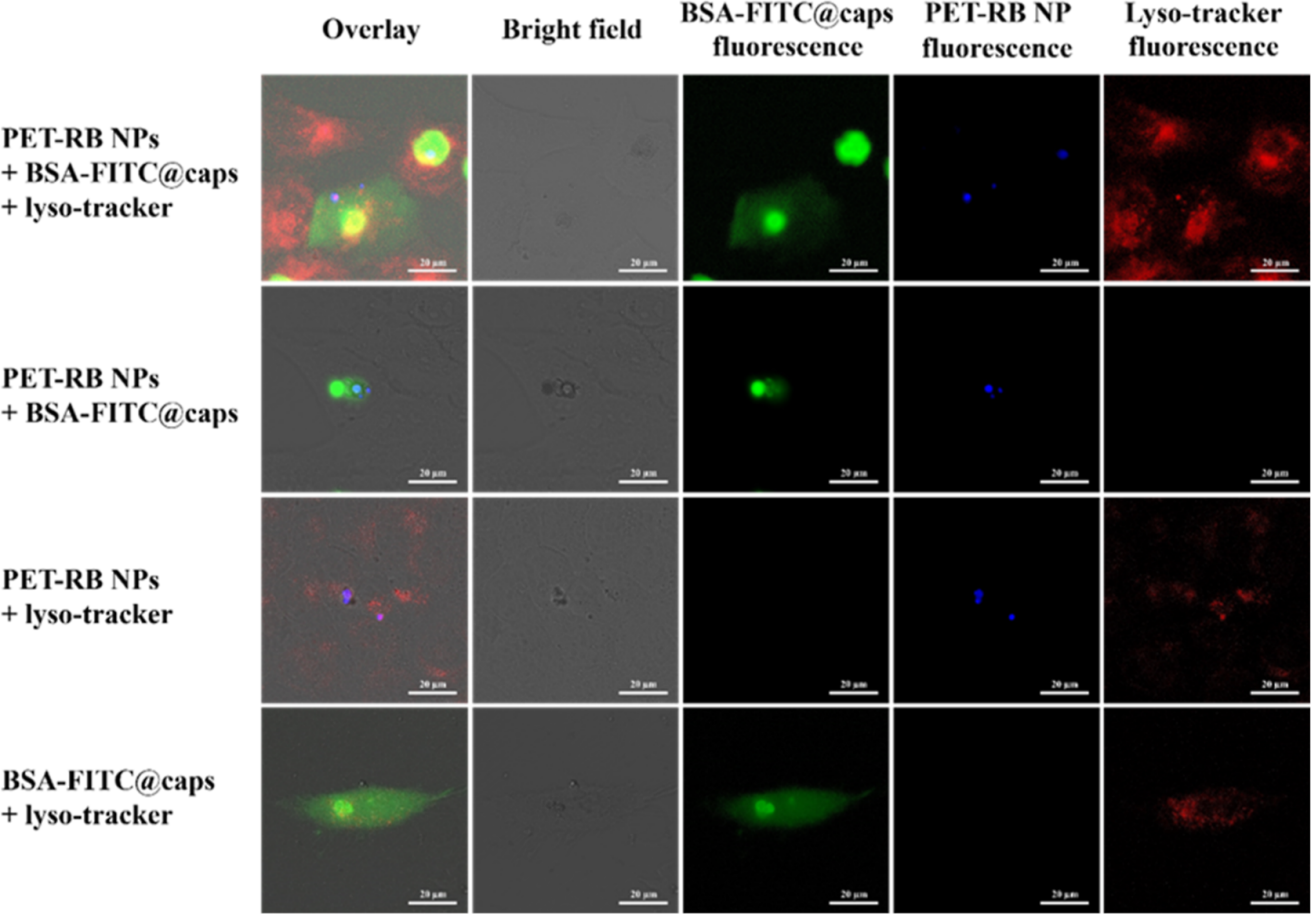
Confocal microscopy images of HeLa cells which had been exposed to optionally PET-RB NPs, BSA-FITC@caps, and stained by LysoTracker. Note that there is some overexposure in the FITC channel in order to see the released BSA-FITC. The RB-fluorescence is shown in false color (blue instead of yellow) in order to facilitate better colocalization analysis by the naked eye. The scale bars represent 20 μm. For additional data, we refer to Figures S16–S18 and Movies S1, S2, and S3.

However, from the data, one can extrapolate that in case PETase@caps capsules are added instead of FITC-BSA@caps, that after intracellular degradation of the shell of PETase@caps, there should be some PETase colocalized with some of the PET-RB NPs. In this way, also hydrolysis of some of the PET NPs by PETase should be possible, as neutral pH is present in the cytosol as well as in the lysosomes with the endocytosed capsules due to the PEI. The precise reaction pathway, however, could not be predicted. In fact, there might be an overlay of both pathways, i.e., part of the enzymatic reaction in the lysosomes where the pH has been buffered by PEI and part in the cytosol, to which parts of the enzymes have been released after transient perforation of the lysosomal membrane due to PEI. Both scenarios warrant for approximately neutral pH.

In the next step, we probed whether PET NPs incorporated by HeLa cells can be degraded by endocytosed PETase@caps. For this, first HeLa cells were incubated with PET-RB NPs. After 1 day of incubation, PET-RB NPs, which had not been endocytosed, were removed by washing. These cells act as a model system for cells contaminated with incorporated nanoplastics. As “treatment”, the cells were exposed to PETase@caps for 1 d. After this, the cells were washed and the RB fluorescence originating from the cells was measured with a microplate reader, see Figure 4. The same procedure without the addition of PETase@caps was used as a control.

**Figure 4:**
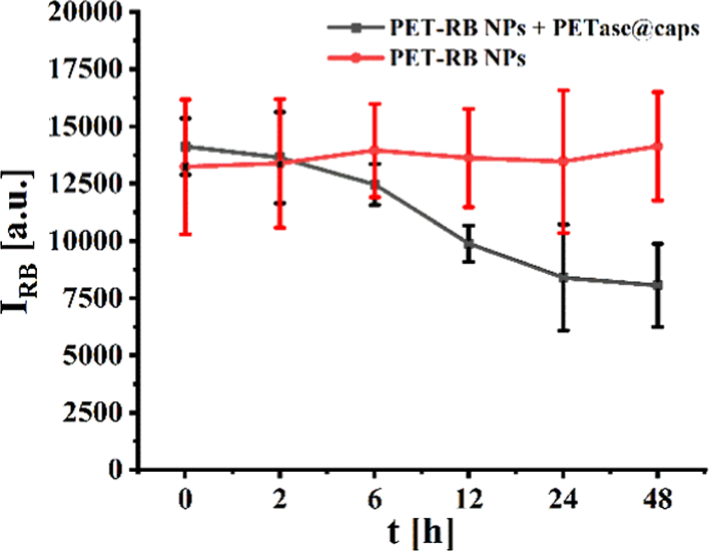
RB fluorescence I_RB_ as recorded from cells which had been prior exposed for 1 day to PET-RB NPs and optionally then for the time *t* with PETase@caps. The data points correspond to the mean values and standard deviations of *n* = 20 readouts in different wells. An independent experiment according to the same procedure is shown in Figure S19.

For the control, the RB fluorescence over time remains constant. This fluorescence is due to internalized PET-RB NPs. While the NPs can be redistributed among daughter cells upon proliferation, this leaves the total amount of NPs inside cells unchanged, and thus, the fluorescence recorded of all cells within one well remains constant. According to previous work, the PET-RB NPs are too big to be efficiently exocytosed,^59–63^ which was extensively investigated by probing for exocytosed NPs or their fragments in the extracellular medium by mass spectrometry. Also, tracing of individual fluorescence NPs showed that there is size limit for efficient exocytosis (and transcytosis).^64^ Further evidence against exocytosis of nondegraded PET-RB NPs being the explanation for the intracellular loss of fluorescence originates directly from Figures 4 and 5b,c. Exocytosis of nondegraded PET-RB NPs would be independent from the presence of PETase@caps. In Figures 4 and 5c, the data show that the intracellular fluorescence of PET-RB-NPs (without PETase@caps) remains constant, thus ruling out efficient exocytosis. In addition, the data shown later in the single NP experiments in Figure 5b indicate a continuous loss of fluorescence of discrete individual PET-RB NPs upon the presence of PETase@caps. In contrast, in case of exocytosis, the intracellular fluorescence of one discrete, individual PET-RB NP should vanish completely in case this particular NP is exocytosed.

**Figure 5:**
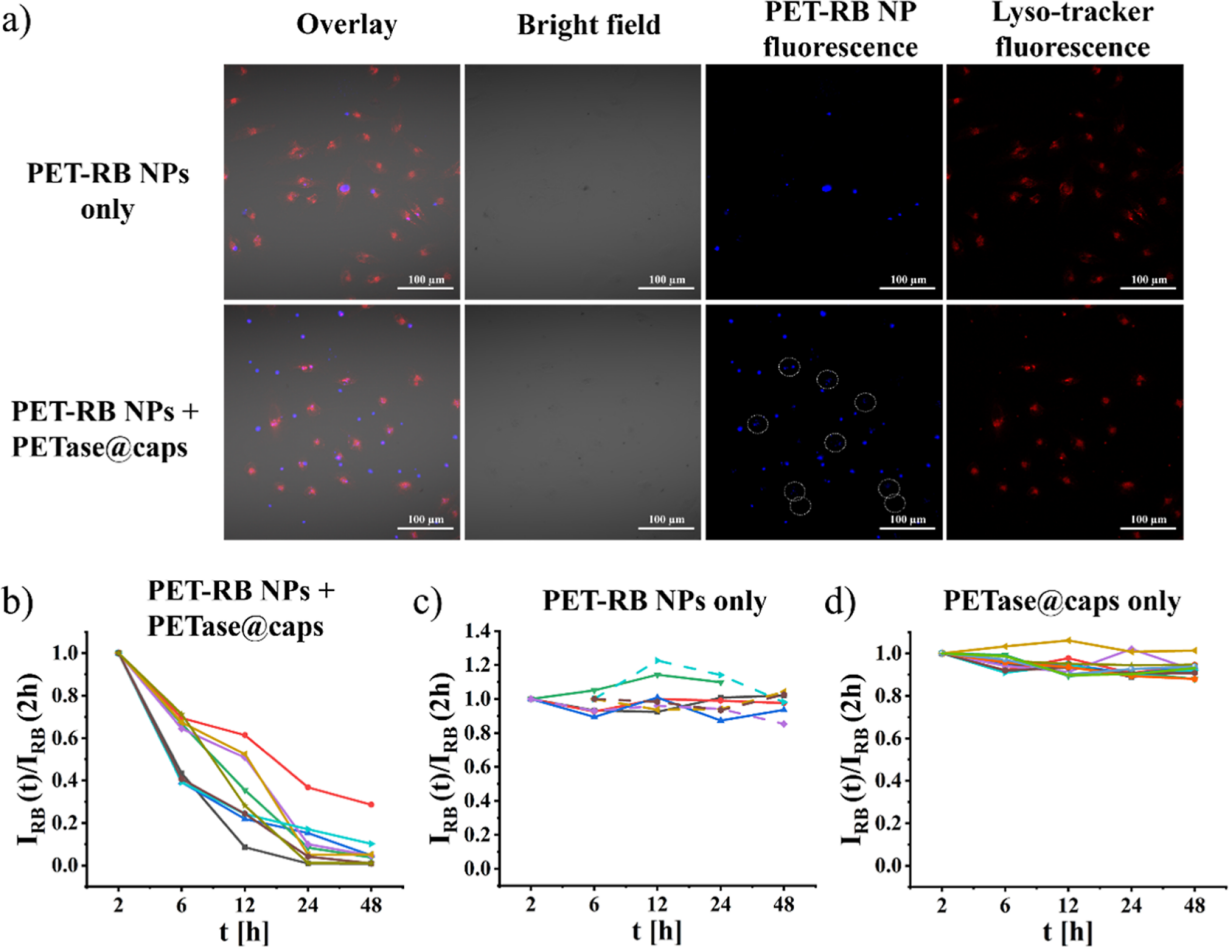
(a) Images of cells which had been incubated with PET-RB NPs and optionally also with PETase@caps, after *t* = 24 h. The lysosomes of cells were stained with LysoTracker deep red. In the case of coincubation of PET-RB NPs with PETase@caps, the circles in the images indicate intracellular PET-RB NPs. The scale bars refer to 100 μm. (b–d). Fluorescence decay of intracellular PET-RB NPs over time. In Figure 5c, the solid and dashed lines represent PET-RB NPs inside and outside of cells, respectively. The data from which Figures (b,c,d) have been composed are shown in Figures S22–S52. Note that in the control Figure 5d, no PET-RB NPs were present, and thus the intensity recorded here refers to the background.

On the other hand, colocalization of PETase@caps and the PET-RB NPs would lead to degradation of the PET-RB NPs. Fragment of the PET-RB NPs may be sufficiently small to be exocytosed. The fluorescence from exocytosed PET-RB NP fragments was washed away before the fluorescence measurement. The temporal decline of the intracellular PET-RB NP fluorescence thus suggests (partial) degradation of the PET-RB NPs and (partial) removal of the fragments by exocytosis. As noninternalized PET-RB NPs had been removed from the extracellular medium before addition of PETase@caps to the cells, this suggested that the degradation of PET-RB NPs has happened intracellularly and not extracellularly. We note that the suggested exocytosis of enzymatically degraded PET-RB NP fragments is only indirectly demonstrated via reduction of the intracellular PET-RB NP fluorescence. There is strong evidence by control measurements that this is not due to a mere photophysical effect such as bleaching, as will be discussed in detail in the following section about single-cell measurements. On the other hand, a direct proof would involve the direct detection of the degradation products in the extracellular medium. In the case of the internalized PET-RB NPs, we unsuccessfully attempted to detect with mass spectrometry (SYNAPT XS from Waters, equipped with q-TOF) two different degradation products in the extracellular medium terephthalic acid (TPA) and bis­(2-hydroxyethyl) terephthalate (BHET). Test in spiked extracellular medium showed that these particular components were not trivial to detect. We were also unable to reliably detect RB fluorescence originating from exocytosed degraded PET-RB NP fragments in the extracellular medium above the background level, which is referred to as the low absolute concentrations in the total volume of the extracellular medium and background fluorescence from the medium (note, in the microplate reader data, the fluorescence is confined to the plane of the cells adherent to the culture surface, leading to a much higher local concentration).

Instead, in the next step, the intracellular degradation of PET-RB NPs was probed in single-cell experiments. For this, cells were incubated with PET-RB NPs. After 1 day of incubation, noninternalized NPs were (optionally) removed by rinsing and changing of the medium. Then, PETase@caps ((DEXS/pARG)_2_/(DEXS/PEI)_2_ shell geometry) was added. After incubation of the time *t*, the lysosomes of cells were optionally stained with LysoTracker Deep Red and then cells were imaged; see Figure 5a bottom row. As controls, either no capsules or no PET-RB NPs were added, see Figure 5a top row (for the details of the procedure, see Figures S20 and S21). In case PET-RB NPs and PETase@caps are present, the fluorescence of PET-RB NPs localized inside cells (indicated with white circles in Figure 5a bottom row) is lower than that of extracellular PET-RB NPs. The fluorescence of extracellular PET-RB NPs in the bottom row of Figure 5a is also similar to the fluorescence of the control PET-RB NPs in the top row of Figure 5a where no PETase@caps was present. This suggests, that upon colocalization of PET-RB NPs and PETase@caps inside cells, PET-RB NPs are (partly) degraded and due to exocytosis of the fragments, the PET-RB NP fluorescence goes down over time. In order to observe this in a more quantitative way, series of images recorded at different time points of incubation *t* were recorded. The fluorescence of the same individual PET-RB NPs was then determined over time. The results shown in Figure 5b–d show that the fluorescence of PET-RB NPs is only reduced over time under the presence of PETase@caps. These quantitative data again indicate intracellular degradation of PET-RB NPs by PETase@caps. The individual raw data are shown in Figures S20 and S22–S52.

As mentioned above for the case of the microplate reader data (cf. Figure 4), in principle, other pathways than degradation of the PET-RB NPs followed by exocytosis of their fragments could be possible and need to be excluded. For this, the single NP data in Figure 5 are in particular helpful. These data were made under dilute conditions where only few NPs are in each cell, where statistically each light spot should correspond to a single PET-RB NP. Thus, while the size of one single PET-RB NP of *d*_c_ = 138 ± 19 nm is below the optical resolution limit, single NPs are tracked in the graphs of Figure 5b–d. In the interpretation of the microplate reader data (Figure 4), it has already been argued that NP efflux via exocytosis of nondegraded PET-RB NPs cannot be the major pathway of intracellular RB fluorescence due to the large size of the NPs. Photobleaching can be excluded from the control data shown in Figure 5c, where there are no enzymes present, and the fluorescence of the PET-RB NPs remains constant over time. We note that for each data series, only 5 time points (i.e., 5 fluorescence images) were recorded and that due to limited excitation, no photobleaching can be expected. There might be reduction in fluorescence due to changes in the state of agglomeration of the NPs. Fluorescence quenching could occur upon agglomeration of PET-RB NPs. However, here, single NPs are tracked and no time-dependent agglomeration (which would infer the merging of different fluorescence spots) was observed. In case the PET-RB NPs were initially agglomerated, deagglomeration could lead to fluorescence reduction due to reduction in aggregation-induced emission. Again, single NP tracking largely excludes this possibility.

Taking the data shown in Figures 4 and 5 together suggests that the presence of PETase@caps in the same cell as PET-RB NPs leads to degradation of the PET-RB NPs, which in principle proves the idea that internalized plastic NPs can be cleared by endocytosed PETase@caps. From the data, it is however not possible to conclude that after a certain exposure time, all PET-RB NPs have been degraded. Figure 4 shows that after 2 days of incubation, there is still fluorescence originating from PET-RB NPs in the cells (data recorded on many cells). Figure 5b suggests that fluorescence of PET-RB NPs goes down over time at the single NP level. After 2 days of incubation, some PET-RB NPs cannot be observed anymore, whereas some other PET-RB NPs still show fluorescence. Even in the case the fluorescence of individual intracellular PET-RB NPs can no longer be recorded, this does not prove that these PET-RB NPs have been fully degraded and their fragments exocytosed. In principle, partly degraded fragments might be spread and fall below the limit of fluorescence detection. In principle, there could be quantitative degradation after longer incubation times. This, however, could not be experimentally addressed in this study due to technical limits, such as the tracing of individual capsules over larger incubation times.

There are issues that need to be solved beyond this proof-of-concept study. First, the degradation products of PET-RB NPs involve PET-dimer (M­(HET)_2_), methyl bis­(2-hydroxyethyl terephthalate), and PET-trimer (M­(HET)_3_), methyl tris­(2-hydroxyethyl terephthalate), which are toxic compounds as well as the terephthalic acid per se.^65^ For reducing the toxicity of plastic NPs, an enzymatic degradation reaction degrading the nontoxic products to simple metabolites would be preferable. Fragments also need to be small enough to be exocytosed. This involves also the question of specificity, i.e., whether different plastic NPs can be specifically degraded in cells by different enzymes. Furthermore, also the intracellular colocalizations of PET-RB NPs and PETase@caps are not fully unraveled yet. This includes also the question for how long the enzymes inside the cells would remain functional.

In addition, data shown here are qualitative but not quantitative. For applications, dose–response behavior would be favorable, i.e., the degree of degradation should be related to the amount of internalized enzyme-loaded capsules. This, however, is technically challenging. At the current state of enzyme-loaded capsules, there will be a significant variation of the amount of functional enzymes per capsules. At a single capsule level, the encapsulation efficiency will vary, as the size of the capsules does. In addition, part of the encapsulated enzymes might be deactivated by adsorption to the capsule walls and may also not be released after degradation of the capsules. There will be also a variation in the temporal response, similar to the PEI-mediated release of genetic material.^66^

There is also the question of the temporal stability of the intracellular PETase. After degradation of the capsules, due to the presence of PEI, part of the PETase will reside in the cytosol (cf. Figure 3). Cells possess mechanisms to clear the cytosol from introduced substances, for example, by autophagocytosis. It is therefore not expected that the PETase could be retained inside cells for extended periods (>several days). With the here shown approach, PETase-loaded cells only would be active toward degradation of PET for a limited time up to some days. Alternatively, one might consider to genetically transfect cells with PETase, which would warrant for the intracellular presence of PETase over longer times. However, this would involve the problem of colocalization with endocytosed PET NPs. PETase produced upon genetic transfection would reside in the cytosol and thus (without PEI) not encounter the PET NPs. While thus encapsulated PETase bears the problem of limited period of intracellular residence, it on the other hand allows for a defined location of delivery.

## Conclusions

This report is a proof of concept study and does not involve the demonstration of an application. Still, one could speculate about potential applications of PETase@caps as “intracellular organs”. The advantage of intracellular enzymes toward the enzymes in the free form is that the enzymes can operate under conditions and locations where free enzymes would be degraded or removed. Inside cells, the enzymes are confined and protected. One scenario would involve enzyme-loaded cells for removing environmental spills of plastic particles. This could involve the specific removal of certain plastics by using specific enzymes. Cells could be confined and collected by coloading them with magnetic nanoparticles and using magnetic field gradients.^66^ Surely, this would not be realistic at large scale spills, but there might be local scenarios for the clearance of plastics by cells in local contamination zones. Apart from the environment, also the human body might be considered as a local contamination scenario. One could think about the in vivo clearance of hazardous plastics. Enzymes could be loaded into macrophages, which are collected in the liver. Contamination of the body with plastic NPs ultimately would lead to uptake of the plastic NPs by macrophages, leading to accumulation in the liver, as reported for other NPs.^67^ Enzymatic degradation of the plastic NPs in the liver by the loaded enzymes would lead to the excretion of small residues, which after exocytosis could be cleared from the body via renal excretion. Such in vivo application obviously also would require nontoxic enzymatic degradation products, i.e., the use of different enzymes. While our proof-of-concept study motivates the principle possibility of such applications, implementation remains a task for the future and is not demonstrated here.

This leads to defining a roadmap toward the next steps. Most importantly, for continuing work, new enzyme–plastics systems should be found, leading in particular to nontoxic degradation products. Then, future work should embrace two directions. (i) Toward a better understanding, in particular for obtaining quantitative data, capsules with better defined properties would be required, involving, for example, a better homogeneity and uniformity in terms of encapsulated enzymes per capsule. Perspectives of advanced layer-by-layer assembly have been highlighted recently.^68^ It would also be of advantage to have degradation products which after exocytosis could be directly detected in the supernatant. As stated above, with the current system, mass spectrometry and fluorescence approached failed. One possible improvement could be the integration of metals (e.g., via chelators) into the plastics NPs, to detect their exocytosis via inductively coupled mass spectrometry (ICP–MS).^63^ Direct detection of extracellular degradation products would allow for quantitative degradation assays. With a new enzyme–plastics system, also more colocalization studies of intracellular enzymes and plastics NPs should be made, which finally would lead to quantitative statistical data. A detailed study about the fate and life cycle of endocytosed enzyme-loaded capsules is needed to determine the time-dependent enzymatic activity per cell. Also, better long-term viability data of enzyme-containing cells (e.g., due to the toxicity of PEI) are needed. (ii) Toward application, the next steps would include changing the batch synthesis of the capsules to synthesis routes allowing for large scale production (better ruling out batch-to-batch variations). Intravenous injection of particle-loaded cells has been reported,^69^ but there is need of more quantitative data, such as quantitatively unravelling the fate of the injected cells, long-term toxicity, etc. Work with nonmammalian animal models could be of advantage in the first steps for allowing better statistics. Potential models include insects or worms. In case of metal-containing plastics, imaging in this case could be done using X-ray fluorescence.^70,71^

## Materials and Methods

For a detailed description, we refer to the Supporting Information. The PET-RB NPs and the PETase@caps capsules were synthesized and characterized following standard procedures.^42,72^ Enzymatic activity was probed “in test tube” with a 2-hydroxyterephthalate (HOTP) assay.^73,74^ Enzymatic activity after endocytosis by HeLa cells was probed by the loss of fluorescence of endocytosed PET-RB NPs, by using either a microplate reader or a confocal microscope. Colocalization experiments between endocytosed PET-RB NPs and endocytosed BSA-FITC@caps capsules (where BSA-FITC was used as a fluorescent model protein emulating PETase) were carried out by confocal microscopy under costaining of endo-/lysosomes.

## Supplementary Material









## Notes

Detailed description of the experimental procedures, some replication data, and microscopy images for obtaining the data used for the single particle study (PDF)
Movie S1: Videos of z-stack images (MP4)
Movie S2: Videos of z-stack images (MP4)
Movie S3: Videos of z-stack images (MP4)

## References

[1] Mitrano D. M., Wick P., Nowack B. (2021). Placing nanoplastics in the context
of global plastic pollution. Nat. Nanotechnol..

[2] Nguyen L. H., Nguyen B.-S., Le D.-T., Alomar T. S., AlMasoud N., Ghotekar S., Oza R., Raizada P., Singh P., Nguyen V.-H. (2023). A concept for the
biotechnological minimizing of emerging
plastics, micro-and nano-plastics pollutants from the environment:
A review. Environ. Res..

[3] Houssini K., Li J., Tan Q. (2025). Complexities of the global plastics supply chain revealed
in a trade-linked material flow analysis. Commun.
Earth Environ..

[4] Dokl M., Copot A., Krajnc D., Fan Y. V., Vujanović A., Aviso K. B., Tan R. R., Kravanja Z., Čuček L. (2024). Global projections
of plastic use, end-of-life fate and potential changes in consumption,
reduction, recycling and replacement with bioplastics to 2050. Sustain. Prod. Consum..

[5] Prata J. C., da Costa J. P., Lopes I., Andrady A. L., Duarte A. C., Rocha-Santos T. (2021). A One Health
perspective of the impacts of microplastics
on animal, human and environmental health. Sci.
Total Environ..

[6] Vethaak A. D., Legler J. (2021). Microplastics and human
health. Science.

[7] Winiarska E., Jutel M., Zemelka-Wiacek M. (2024). The potential
impact of nano-and
microplastics on human health: Understanding human health risks. Environ. Res..

[8] Li Y., Tao L., Wang Q., Wang F., Li G., Song M. (2023). Potential
Health Impact of Microplastics: A Review of Environmental Distribution,
Human Exposure, and Toxic Effects. Environ Health.

[9] Xia H., Wang F., Song M. (2025). Spotlight
Nanotoxicology: The Root
of Toxicity of Nanoplastics. Environ Health.

[10] Yan X., Chen H., Jia C., Zhang J., Huang M., Wang S., Guo X., Yue T., Chen L., Zhou Q., Qu G., Zhu H., Jiang G., Yan B. (2025). Nanoplastics Toxicity Is a Subset
of Nanotoxicology, Not a Separate
Field. Environ Health.

[11] Marcellus K. A., Prescott D., Scur M., Ross N., Gill S. S. (2025). Exposure
of Polystyrene Nano- and Microplastics in Increasingly Complex In
Vitro Intestinal Cell Models. Nanomaterials.

[12] Wang M., Fang H.-T., Tan Q.-G., Ji R., Miao A.-J. (2025). Size-Dependent
Toxicity of Polystyrene Nanoplastics to Tetrahymena thermophila: A
Toxicokinetic–Toxicodynamic Assessment. Environ. Sci. Technol..

[13] Shi R., Liu W., Lian Y., Wang X., Men S., Zeb A., Wang Q., Wang J., Li J., Zheng Z., Zhou Q., Tang J., Sun Y., Wang F., Xing B. (2024). Toxicity Mechanisms of Nanoplastics on Crop Growth, Interference
of Phyllosphere Microbes, and Evidence for Foliar Penetration and
Translocation. Environ. Sci. Technol..

[14] Zhou Y., Zhou X.-X., Jiang H., Liu W., Chen F., Gardea-Torresdey J. L., Yan B. (2024). In Vitro Toxicity and
Modeling Reveal
Nanoplastic Effects on Marine Bivalves. ACS
Nano.

[15] Ma, Q. ; Lei, J. ; Pang, Y. ; Shen, Y. ; Zhang, T. Neurotoxicity of Micro- and Nanoplastics: A Comprehensive Review of Central Nervous System Impacts; Environment & Health, 2025. 10.1021/envhealth.5c00087 PMC12813711 41562032

[16] Zhao Q., Fang Z., Wang P., Qian Z., Yang Y., Ran L., Zheng J., Tang Y., Cui X., Li Y.-Y., Zhang Z., Jiang H. (2025). Polylactic Acid Micro/Nanoplastic
Exposure Induces Male Reproductive Toxicity by Disrupting Spermatogenesis
and Mitochondrial Dysfunction in Mice. ACS Nano.

[17] Pikuda O., Xu E. G., Berk D., Tufenkji N. (2019). Toxicity Assessments
of Micro- and Nanoplastics Can Be Confounded by Preservatives in Commercial
Formulations. Environ. Sci. Technol. Lett..

[18] Shaoyong W., Sun L., Gan Y., Jin H., Wang W., Yin L., Wang Y., Jin M. (2024). Sight of Aged
Microplastics Adsorbing
Heavy Metal Exacerbated Intestinal Injury: A Mechanistic Study of
Autophagy-Mediated Toxicity Response. ACS Nano.

[19] Zhang Z., Dong X., Wan W., Guo H., Sun R., Feng H., Wang M., Wang Z., Jin H., Sun J., Xia Q., Zhao Q., Shen D., Gao Z., Liu Y. (2024). Unraveling Intracellular Protein Corona Components
of Nanoplastics
via Photocatalytic Protein Proximity Labeling. Anal. Chem..

[20] Li X., He E., Xia B., Liu Y., Zhang P., Cao X., Zhao L., Xu X., Qiu H. (2021). Protein corona-induced
aggregation of differently sized nanoplastics: impacts of protein
type and concentration. Environ. Sci.: Nano.

[21] Xiao S., Wang J., Digiacomo L., Amici A., De Lorenzi V., Pugliese L. A., Cardarelli F., Cerrato A., Laganà A., Cui L., Papi M., Caracciolo G., Marchini C., Pozzi D. (2024). Protein corona
alleviates adverse biological effects of nanoplastics in breast cancer
cells. Nanoscale.

[22] Dawson A. L., Bose U., Ni D., Nelis J. L. D. (2024). Unravelling protein
corona formation on pristine and leached microplastics. Microplastics and Nanoplastics.

[23] Shieh P., Zhang W., Husted K. E., Kristufek S. L., Xiong B., Lundberg D. J., Lem J., Veysset D., Sun Y., Nelson K. A. (2020). Cleavable
comonomers enable degradable,
recyclable thermoset plastics. Nature.

[24] Wimberger L., Ng G., Boyer C. (2024). Light-driven
polymer recycling to monomers and small
molecules. Nat. Commun..

[25] Choi S. Y., Lee Y., Yu H. E., Cho I. J., Kang M., Lee S. Y. (2023). Sustainable
production and degradation of plastics using microbes. Nature Microbiology.

[26] Mohanan N., Montazer Z., Sharma P. K., Levin D. B. (2020). Microbial
and enzymatic
degradation of synthetic plastics. Front. Microbiol..

[27] Spínola-Amilibia M., Illanes-Vicioso R., Ruiz-López E., Colomer-Vidal P., Rodriguez-Ventura F., Peces Pérez R., Arias C. F., Torroba T., Solà M., Arias-Palomo E. (2023). Plastic degradation
by insect hexamerins: Near-atomic resolution structures of the polyethylene-degrading
proteins from the wax worm saliva. Sci. Adv..

[28] Yoshida S., Hiraga K., Takehana T., Taniguchi I., Yamaji H., Maeda Y., Toyohara K., Miyamoto K., Kimura Y., Oda K. (2016). A bacterium that degrades
and assimilates
poly (ethylene terephthalate). Science.

[29] Islam Z. F., Cherepanov P. V., Xu W., Hayden H. L., Colombi E., Lin Z., Mazaheri O., Caruso F., Chen D., Hu H.-W. (2025). Native
polymer degradation capacity of microorganisms in agricultural soils. Science of The Total Environment.

[30] Tournier V., Duquesne S., Guillamot F., Cramail H., Taton D., Marty A., André I. (2023). Enzymes’ power for plastics
degradation. Chem. Rev..

[31] Chow J., Perez-Garcia P., Dierkes R., Streit W. R. (2023). Microbial enzymes
will offer limited solutions to the global plastic pollution crisis. Microb Biotechnol.

[32] Han S.-W., Choi J., Ryu K.-Y. (2024). Recent progress and future directions
of the research on nanoplastic-induced neurotoxicity. Neural Regeneration Research.

[33] Liu Y.-Y., Liu J., Wu H., Zhang Q., Tang X.-R., Li D., Li C.-S., Liu Y., Cao A., Wang H., Endocytosis D. (2022). Endocytosis, Distribution, and Exocytosis
of Polystyrene
Nanoparticles in Human Lung Cells. Nanomaterials.

[34] Canton I., Battaglia G. (2012). Endocytosis at the nanoscale. Chem. Soc. Rev..

[35] Rennick J. J., Johnston A. P., Parton R. G. (2021). Key principles and methods for studying
the endocytosis of biological and nanoparticle therapeutics. Nat. Nanotechnol..

[36] Nazarenus M., Abasolo I., García-Aranda N., Voccoli V., Rejman J., Cecchini M., Schwartz S., RiveraGil P., Parak W. J. (2015). Polymer Capsules
as a Theranostic
Tool for a Universal In Vitro Screening Assay—The Case of Lysosomal
Storage Diseases. Particle & Particle Systems
Characterization.

[37] Rivera_Gil P., Nazarenus M., Ashraf S., Parak W. J. (2012). pH sensitive capsules
as intracellular optical reporters for monitoring lysosomal pH changes
upon stimulation. Small.

[38] Zimmermann W., Billig S. (2010). Enzymes for the biofunctionalization
of poly (ethylene
terephthalate). Biofunctionalization of Polymers
and their Applications.

[39] Rejman J., Nazarenus M., Aberasturi D. J. d., Said A. H., Feliu N., Parak W. J. (2016). Some thoughts
about the intracellular location of nanoparticles
and the resulting consequences. J. Colloid Interface
Sci..

[40] Lee K., Jung I., Odom T. W. (2022). Delivery
Order of Nanoconstructs
Affects Intracellular Trafficking by Endosomes. J. Am. Chem. Soc..

[41] Buchholz, P. C. F. ; Feuerriegel, G. ; Zhang, H. ; Perez-Garcia, P. ; Nover, L.-L. ; Chow, J. ; Streit, W. R. ; Pleiss, J. Plastics degradation by hydrolytic enzymes: The Plastics-Active Enzymes Database - PAZy. https://pazy.eu/doku.php?id=start (accessed 1 15, 2025). 10.1002/prot.26325 35175626

[42] Johnson L.
M., Mecham J. B., Krovi S. A., Moreno Caffaro M. M., Aravamudhan S., Kovach A. L., Fennell T. R., Mortensen N. P. (2021). Fabrication
of polyethylene terephthalate (PET) nanoparticles with fluorescent
tracers for studies in mammalian cells. Nanoscale
Advances.

[43] Zhang H., Dierkes R. F., Perez-Garcia P., Costanzi E., Dittrich J., Cea P. A., Gurschke M., Applegate V., Partus K., Schmeisser C. (2024). The metagenome-derived
esterase PET40
is highly promiscuous and hydrolyses polyethylene terephthalate (PET). FEBS J..

[44] Jones A. T., Gumbleton M., Duncan R. (2003). Understanding endocytic pathways
and intracellular trafficking: a prerequisite for effective design
of advanced drug delivery systems. Advanced
drug delivery reviews.

[45] Sousa
de Almeida M., Susnik E., Drasler B., Taladriz-Blanco P., Petri-Fink A., Rothen-Rutishauser B. (2021). Understanding nanoparticle endocytosis
to improve targeting strategies in nanomedicine. Chem. Soc. Rev..

[46] De
Koker S., De Cock L. J., Rivera_Gil P., Parak W. J., Velty R. A., Vervaet C., Remon J. P., Grooten J., De Geest B. G. (2011). Polymeric multilayer capsules delivering
biotherapeutics. Adv. Drug Delivery Rev..

[47] Zhu D., Roy S., Liu Z., Weller H., Parak W., Feliu N. (2019). Remotely controlled
opening of delivery vehicles and release of cargo by external triggers. Adv. Drug Delivery Rev..

[48] Caruso F., Caruso R. A., Möhwald H. (1998). Nanoengineering
of Inorganic and
Hybrid Hollow Spheres by Colloidal Templating. Science.

[49] Decher G. (1997). Fuzzy nanoassemblies:
Toward Layered Polymeric Multicomposites. Science.

[50] Rivera-Gil P., De Koker S., De Geest B. G., Parak W. J. (2009). Intracellular processing
of proteins mediated by biodegradable polyelectrolyte capsules. Nano Lett..

[51] Roy S., Zhu D., Parak W. J., Feliu N. (2020). Lysosomal Proton Buffering of Poly­(ethylenimine)
Measured In Situ by Fluorescent pH-Sensor Microcapsules. ACS Nano.

[52] De
Cock L. J., De Koker S., De Geest B. G., Grooten J., Vervaet C., Remon J. P., Sukhorukov G. B., Antipina M. N. (2010). Polymeric Multilayer Capsules in Drug Delivery. Angew. Chem., Int. Ed..

[53] Thottacherry J. J., Sathe M., Prabhakara C., Mayor S. (2019). Spoiled for Choice:
Diverse Endocytic Pathways Function at the Cell Surface. Annu. Rev. Cell Dev. Biol..

[54] Kreft O., Javier A. M., Sukhorukov G. B., Parak W. J. (2007). Polymer microcapsules
as mobile local pH-sensors. Journal Of Materials
Chemistry.

[55] Kastl L., Sasse D., Wulf V., Hartmann R., Mircheski J., Ranke C., Carregal-Romero S., Martínez-López J. A., Fernández-Chacón R., Parak W. J., Elsasser H. P., Rivera_Gil P. (2013). Multiple internalization
pathways of polyelectrolyte
multilayer capsules into mammalian cells. ACS
Nano.

[56] Kirchner C., Javier A. M., Susha A. S., Rogach A. L., Kreft O., Sukhorukov G. B., Parak W. J. (2005). Cytotoxicity of nanoparticle-loaded
polymer capsules. Talanta.

[57] Zyuzin M. V., Díez P., Goldsmith M., Carregal-Romero S., Teodosio C., Rejman J., Feliu N., Escudero A., Almendral M. J. s., Linne U., Peer D., Fuentes M., Parak W. J. (2017). Comprehensive and Systematic Analysis
of the Immunocompatibility
of Polyelectrolyte Capsules. Bioconjugate Chem..

[58] Ganas C., Weiß A., Nazarenus M., Rösler S., Kissel T., Rivera_Gil P., Parak W. J. (2014). Biodegradable capsules
as non-viral vectors for in vitro delivery of PEI/siRNA polyplexes
for efficient gene silencing. J. Controlled
Release.

[59] Liu Z., Escudero A., Carrillo-Carrion C., Chakraborty I., Zhu D., Gallego M., Parak W. J., Feliu N. (2020). Biodegradation of Bi-Labeled
Polymer-Coated Rare-Earth Nanoparticles in Adherent Cell Cultures. Chem. Mater..

[60] Liu Z., Zimpel A., Lächelt U., Pozzi M., Gonzalez M. G., Chakraborty I., Wuttke S., Feliu N., Parak W. J. (2023). Uptake
and Intracellular Fate of Fluorophore Labeled Metal–Organic-Framework
(MOF) Nanoparticles. Environ Health.

[61] Sun X., Gamal M., Nold P., Said A., Chakraborty I., Pelaz B., Schmied F., von Pückler K., Figiel J., Zhao Y., Brendel C., Hassan M., Parak W. J., Feliu N. (2019). Tracking stem cells and macrophages
with gold and iron oxide nanoparticles – The choice of the
best suited particles. Appl. Mater. Today.

[62] Kang Y., Nack L. M., Liu Y., Qi B., Huang Y., Liu Z., Chakraborty I., Schulz F., Ahmed A. A. A., Clavo
Poveda M., Hafizi F., Roy S., Mutas M., Holzapfel M., Sanchez-Cano C., Wegner K. D., Feliu N., Parak W. J. (2022). Quantitative considerations about the size dependence
of cellular entry and excretion of colloidal nanoparticles for different
cell types. ChemTexts.

[63] Feng M., Werner S., Qi B., Li J., Schulz F., Parak W. J. (2025). Quantification of optimizing cellular
uptake and excretion
of nanoparticles by aggregation and de-aggregation mediated size changes. Nano Today.

[64] Kuhn D. A., Hartmann R., Fytianos K., Petri-Fink A., Rothen-Rutishauser B., Parak W. J. (2015). Cellular uptake
and cell-to-cell
transfer of polyelectrolyte microcapsules within a triple co-culture
system representing parts of the respiratory tract. Sci. Technol. Adv. Mater..

[65] Djapovic M., Milivojevic D., Ilic-Tomic T., Lješević M., Nikolaivits E., Topakas E., Maslak V., Nikodinovic-Runic J. (2021). Synthesis
and characterization of polyethylene terephthalate (PET) precursors
and potential degradation products: Toxicity study and application
in discovery of novel PETases. Chemosphere.

[66] Ochs M., Carregal Romero S., Rejman J., Braeckmans K., De Smedt S. C., Parak W. J. (2013). Light-addressable
capsules as caged
compound matrix for controlled in vitro release. Angew. Chem., Int. Ed..

[67] Kreyling W. G., Abdelmonem A. M., Ali Z., Alves F., Geiser M., Haberl N., Hartmann R., Hirn S., de Aberasturi D. J., Kantner K., Khadem-Saba G., Montenegro J. M., Rejman J., Rojo T., de Larramendi I. R., Ufartes R., Wenk A., Parak W. J. (2015). In vivo integrity
of polymer-coated gold nanoparticles. Nature
Nanotechnol.

[68] Zhao S., Caruso F., Dähne L., Decher G., Geest B. G. D., Fan J., Feliu N., Gogotsi Y., Hammond P. T., Hersam M. C., Khademhosseini A., Kotov N., Leporatti S., Li Y., Lisdat F., Liz-Marzán L.
M., Moya S., Mulvaney P., Rogach A. L., Roy S., Shchukin D. G., Skirtach A. G., Stevens M. M., Sukhorukov G. B., Weiss P. S., Yue Z., Zhu D., Parak W. J. (2019). The Future
of Layer-by-Layer Assembly: A Tribute to ACS Nano Associate Editor
Helmuth Möhwald. ACS Nano.

[69] Xu L., Xu M., Sun X., Feliu N., Feng L., Parak W. J., Liu S. (2023). Quantitative Comparison of Gold Nanoparticle
Delivery via the Enhanced
Permeation and Retention (EPR) Effect and Mesenchymal Stem Cell (MSC)-Based
Targeting. ACS Nano.

[70] Zhu D., Brückner D., Sosniok M., Skiba M., Feliu N., Gallego M., Liu Y., Schulz F., Falkenberg G., Parak W. J., Sanchez-Cano C. (2025). Size-dependent penetration depth
of colloidal nanoparticles into cell spheroids. Adv. Drug Delivery Rev..

[71] Staufer T., Kopatz V., Pradel A., Brodie T., Kuhrwahl R., Stroka D., Wallner J., Kenner L., Pichler V., Grüner F., Mitrano D. M. (2025). Biodistribution of nanoplastics in
mice: advancing analytical techniques using metal-doped plastics. Commun. Biol..

[72] Brkovic N., Zhang L., Peters J. N., Kleine-Doepke S., Parak W. J., Zhu D. (2020). Quantitative Assessment
of Endosomal
Escape of Various Endocytosed Polymer-Encapsulated Molecular Cargos
upon Photothermal Heating. SMALL.

[73] Chaves M. R., Lima M. L., Malafatti-Picca L., De Angelis D. A., Castro A. M. d., Valoni E. ´., Marsaioli A. J. (2018). A practical
fluorescence-based screening protocol for polyethylene terephthalate
degrading microorganisms. J. Braz. Chem. Soc..

[74] Pfaff, L. ; Breite, D. ; Badenhorst, C. P. ; Bornscheuer, U. T. ; Wei, R. Fluorimetric high-throughput screening method for polyester hydrolase activity using polyethylene terephthalate nanoparticles. Methods Enzymol. 2021, 648, 253–270 10.1016/bs.mie.2020.11.003. 33579406

