# Supplementary material for: Enzyme-Loaded Microcapsules as Intracellular Organelles for the Degradation of Nanoplastics by Cells
Source: ACS Nano. 2025 Dec 16;20(5):4106–15. doi: 10.1021/acsnano.5c15213 (PMC12895564; doi:10.1021/acsnano.5c15213)
Supplement: Supplementary file 1 [file nn5c15213_si_001.pdf]

Supporting Information

Enzyme-loaded microcapsules as intracellular organelles for the degradation of nanoplastics by cells

Xin Liu[1], Jennifer Chow[2], Wenbo Wang[1], Robert Dierkes[2], Neus Feliu[3], Florian Schulz[1], Wolfgang R. Streit[2*], Wolfgang J. Parak[1*]

[1]Fachbereich Physik, Center for Hybrid Nanostructures (CHyN), Universität Hamburg, 22761 Hamburg, Germany
[2]Department of Microbiology and Biotechnology, Universität Hamburg, 22609 Hamburg, Germany
[3]Fraunhofer Center for Applied Nanotechnology (IAP-CAN), 20146 Hamburg, Germany
*corresponding authors: wolfgang.streit@uni-hamburg.de, wolfgang.parak@uni-hamburg.de

1) Synthesis of nano- and microparticles
2) Characterization of nano- and microparticles
3) Degradation properties ("in test tube")
4) Basic cell culture procedures
5) Colocalization experiments
6) Intracellular degradation (multiple cells)
7) Intracellular degradation (single cells)
8) References

1) Synthesis of nano- and microparticles

1.1) Reagents
1.2) Synthesis of polyethylene terephthalate nanoparticles (PET NPs)
1.3) Synthesis of polyelectrolyte microparticles (caps)

1.1) Reagents

Trisodium citrate dihydrate (#3580.4), hydrochloric acid (HCl, #4625.1) and sodium hydroxide (NaOH, #6771.1) were purchased from Carl Roth (Germany). Polyethylene terephthalate filament (PET, # ES30-FB-000110) was purchased from Goodfellow (Germany). rhodamine B (RB, #83689), hexafluoroisopropanol (HFIP, #105228), bovine serum albumin (BSA, #A2153), branched poly(ethyleneimine) (PEI, $M_w \approx 25$ kDa, #408727), poly-L-arginine hydrochloride (pARG, $M_w \approx 15$-70 kDa, #P7762), dextran sulfate sodium salt (DEXS, $M_w \approx 10$ kDa, #51227), calcium chloride dihydrate ($CaCl_2 \cdot 2H_2O$, #223506), sodium carbonate (Na_2CO_3, #S7795), sodium phosphate dibasic (Na_2HPO_4, #S3264), potassium phosphate monobasic (KH_2PO_4, #P5655), ethylenediamine-tetra-acetic acid disodium salt dihydrate (EDTA disodium salt, #E5134) and iron(II) sulfate heptahydrate ($FeSO_4 \cdot 7H_2O$, #F7002) were purchased from Sigma-Aldrich (Germany). Hydrogen peroxide (H_2O_2, #23622) was purchased from Avantor (Germany). Ultrapure double distilled water (ddH2O, MilliQ) with a resistivity greater than 18.2 MΩ cm^{-1} was used for all experiments.

1.2) Synthesis of polyethylene terephthalate nanoparticles (PET NPs)

According to a published protocol [1], PET solution was prepared by adding 0.58 g of PET filament into 35 mL of hexafluoroisopropanol (HFIP). The solution was mixed thoroughly by gentle stirring at room temperature (RT) for 1 h. 10 mL of PET solution was added dropwise at 1 mL min^{-1} using a syringe pump (LEGATO® 111 SYRINGE PUMP, KD Scientific, USA) with a 10 mL glass syringe (FORTUNA Optima®, Poulten & Graf GmbH, Germany) into 75 mL of MilliQ water at RT, resulting in precipitation of PET NPs. The resulting mixtures were then placed into a round bottom flask and mildly heated (55 °C) in a rotary evaporator under vacuum to remove residual HFIP. Upon reduction of the volume in the round bottom flask (~30 mL), 75 mL of MilliQ water was added, and the solution was concentrated by rotary evaporation for a second time. To remove residual HFIP, the suspension of PET NPs was subjected to centrifugation at 3000 rpm for 5 min at room temperature. Subsequently, the supernatant was removed, and the PET NPs were resuspended in 5 mL of a BSA solution (0.5 mg/mL, pH 8.2). This process was repeated three times, with the supernatant discarded and fresh water added each time. Finally, the PET NPs were resuspended in 5 mL of a BSA solution (0.5 mg/mL, pH 8.2) and stored at 4° C until further use. PET NPs containing rhodamine B (PET-RB) were formulated using the same approach with the following modification: An aliquot of 1 mL of RB solution (0.05 mg/mL) was added to the PET solution prior to precipitation into MilliQ water.

The molar mass M_w(PET NPs) of the PET NPs is estimated from the volume V_c of one PET NP which is assumed as sphere with diameter $d_c \approx 92.33$ nm (see Figure S3), the density of PET $\rho_{PET} = 1.38$ g/cm^3 [2] as $M_w(\text{PET NPs}) = \rho_{PET} \cdot V_c \cdot N_A = \rho_{PET} \cdot (4/3) \cdot \pi \cdot (d_c/2)^3 \cdot N_A = \rho_{PET} \cdot (\pi/6) \cdot (d_c)^3 \cdot N_A = 1.38 \cdot 10^3$ g·m⁻

$^3 \cdot(\pi/6) \cdot(92.33\cdot10^{-9}$ m$)^3\cdot 6.02\cdot10^{23}$ mol$^{-1} \approx 3.4\cdot10^6$ g/mol, using Avogadro's number $N_A = 6.02\cdot10^{23}$ mol^{-1}.

1.3) Synthesis of polyelectrolyte microparticles (caps)

In a typical synthesis following a published protocol [3, 4], first micrometer-sized $CaCO_3$ template cores were fabricated. 615 µL of 0.33 M Na_2CO_3 solution was mixed with 400 µL of 5 mg/mL molecular cargo (PETase or BSA-FITC) in a 20 mL glass vial. Thorough mixing by vigorous stirring at 900 rpm was followed by the addition of 615 µL of 0.33 M $CaCl_2\cdot 2H_2O$ solution. The vial was kept at RT under magnetic stirring for 30 s, and then left standing for 3 min to stop $CaCO_3$ growth. The $CaCO_3$ cores were collected by centrifugation at 3000 rpm for 30 s and then washed with water 3 times by centrifugation at 3000 rpm for 30 s at room temperature, discarding the supernatant, and adding fresh water. Polyelectrolyte capsules were then synthesized by layer-by-layer (lbl) assembly of oppositely charged polymers around the template cores, see Figure S1 [5]. For this, the $CaCO_3$ cores were resuspended in 1 mL of negatively charged DEXS (2 mg/mL, 0.1 M NaCl, pH 6.5), sonicated for 2 min, kept under continuous shaking for 10 min, and washed with water three times by centrifugation at 3000 rpm for 30 s at room temperature, discarding the supernatant, and adding fresh water. Afterwards, 1 mL of positively charged pARG (2 mg/mL, 0.1 M NaCl, pH 6.5) was added to the precipitate after washing, the particles were resuspended, and a similar procedure was followed as described for the DEXS shell generation. With the same steps, additional layers of DEXS and pARG layers were formed, resulting in 4 (DEXS/pARG) bilayers. The built-up of layers was monitored by zeta potential measurements (Figure S2), indicating the formation of each new layer by a reversal in the sign of the potential [6]. Subsequently, the polymer-coated $CaCO_3$ particles were incubated overnight in 2 mL of EDTA (0.1 M, pH 6.5) at 4 ℃ to dissolve the $CaCO_3$ cores. The hollow capsules (i.e. the polyelectrolyte shells) were collected by centrifugation at 1800 rpm for 10 min and then washed with water three times to remove excess EDTA, by centrifugation at 1800 rpm for 10 min at room temperature, discarding the supernatant, and adding fresh water. Finally, the capsules with $(DEXS/pARG)_4$ shell geometry were resuspended in 1 mL of water and stored at 4 °C until further use. Alternatively, in order to provide proton buffering capability to the capsules, the last 2 bilayers were made by DEXS and positively charged PEI (2 mg/mL, 0.1 M NaCl, pH 6.5), leading to a final shell geometry of $(DEXS/pARG)_2/(DEXS/PEI)_2$. For both geometries the PETase or BSA-FITC remained inside the capsule cavities upon dissolution of the template cores with EDTA.

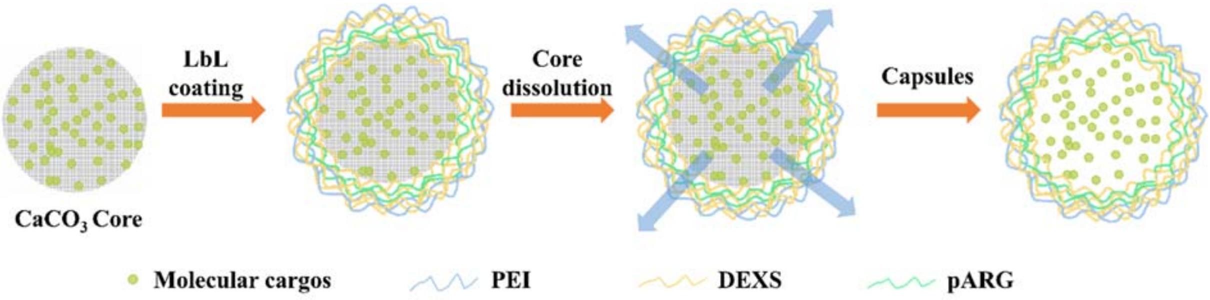

Figure S1. Schematic illustration of the synthesis of a multilayer polyelectrolyte capsules. The molecular cargo is either BSA-FITC or PETase. The final shell geometry of the here depicted capsule is $(DEXS/pARG)_2/(DEXS/PEI)_2$.

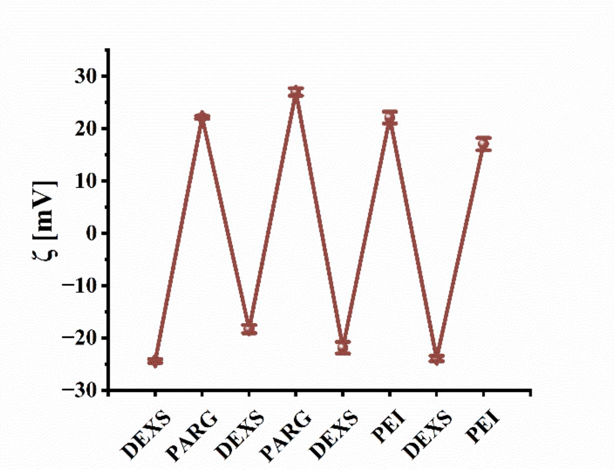

Figure S2. Zeta potential ζ of polyelectrolyte layers around $CaCO_3$ cores with the final sequence $(DEXS/pARG)_2/(DEXS/PEI)_2$, as measured in MilliQ water with laser Doppler anemometry (LDA; Zetasizer Particle Analyzer ZEN3600, Malvern) after the addition of each individual layer.

2) Characterization of nano- and microparticles

2.1) Morphology characterization
2.2) Dynamic light scattering
2.3) Fourier transformed infrared (FTIR) characterization
2.4) Characterization of optical properties
2.5) Stability of properties in different media
2.6) Estimation of the amount of PETase per capsule

2.1) Morphology characterization

The morphology of the PET NPs was determined with a transmission electron microscope (TEM, JEM 1011, JEOL) on dried samples. A representative image is shown in Figure 1a. From a series of such images (see Figure S2) and the respective distribution of the core diameter the mean PET NP diameter was determined to be $d_c \approx 92$ nm The capsules were imaged with optical bright-field microscopy (see Figure 1b) and fluorescence microscopy (LSM880 system, Zeiss; the continuous wave laser was generated by a two-photon generator (Ti:Sa Laser Mai Tai, Spectra Physics)). In Figure S4 images of BSA-FITC encapsulated with $(DEXS/pARG)_2/(DEXS/PEI)_2$ (i.e. BSA-FITC@caps) is shown. From the images of the bright-field channel the mean capsule diameter was determined to be $d_c \approx 3.3$ µm.

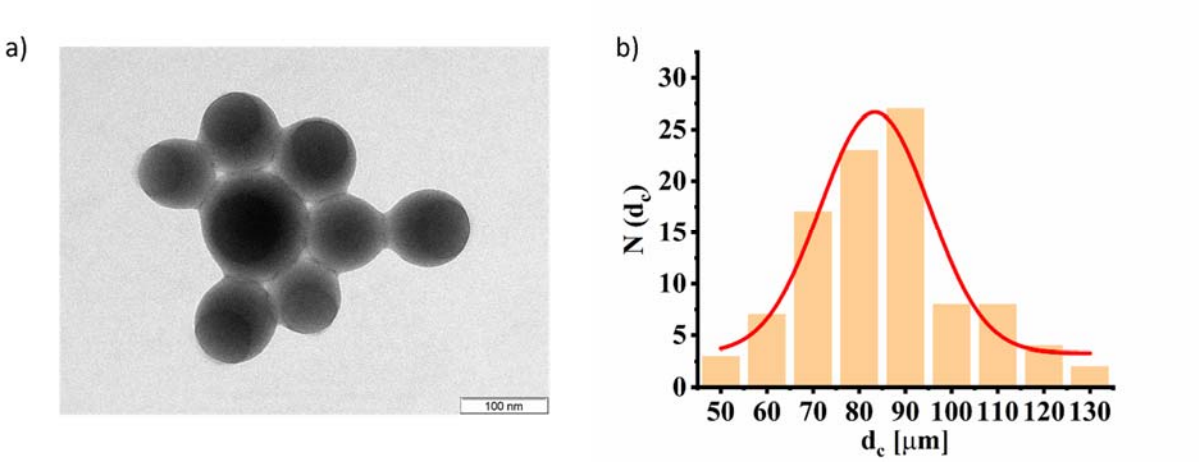

Figure S3. TEM images of PET NPs and the corresponding distribution $N(d_c)$ in which the number of counts for NPs with a diameter d_c is given. The scale bar corresponds to 100 nm. The mean diameter as derived from the size distribution is $d_c \approx 92.33$ nm.

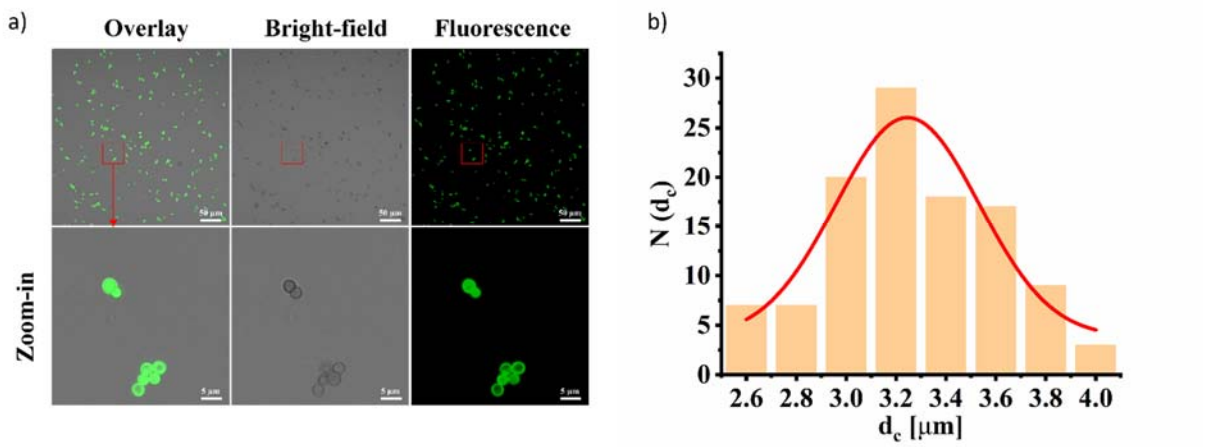

Figure S4. Optical microscopy images (bright-field and FITC fluorescence) of BSA-FITC@caps ($(DEXS/pARG)_2/(DEXS/PEI)_2$ shell geometry). The lower row shows zoom-in into the red indicated boxes in the upper row. The scale bares are 50 µm and 5 µm for the images in the upper and lower row, respectively. Corresponding size distribution showing the number of counts $N(d_c)$ of capsules with a diameter d_c. From the histogram the mean capsule diameter was determined to be $d_c \approx 3.3$ µm.

2.2) Dynamic light scattering

Dynamic light scattering (DLS) analysis for obtaining the hydrodynamic diameter d_h of the particles in MilliQ water was conducted using a Malvern Zetasizer Particle Analyzer (ZEN3600, Malvern). From the intensity-weighted size distributions $I(d_h)$ the mean hydrodynamic diameters of PET-RB NPs and BSA-FITC@caps ($(DEXS/pARG)_2/(DEXS/PEI)_2$ shell geometry) were determined to be $d_h = 256.38 \pm 9.67$ nm and $d_h = 2925 \pm 210.70$ nm, respectively, see Figure S5.

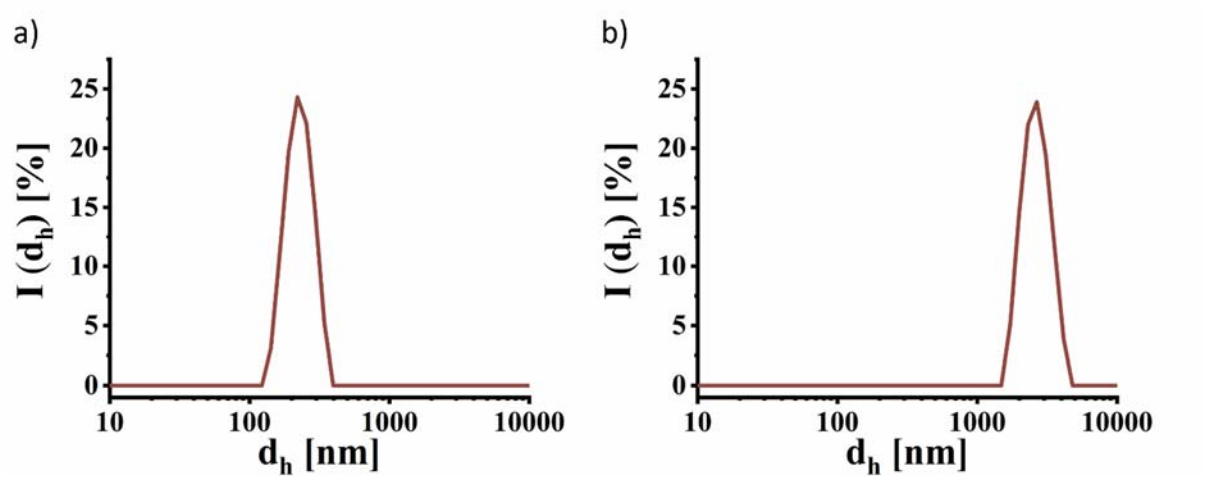

Figure S5. Intensity-weighted distribution of the hydrodynamic diameters for a) PET-RB NPs and b) BSA-FITC@caps ($(DEXS/pARG)_2/(DEXS/PEI)_2$ shell geometry) rhodamine B. The DLS profiles are the average of five DLS runs.

2.3) Fourier transformed infrared (FTIR) characterization

Fourier-transform infrared spectra (FTIR) were collected using an FTIR spectrometer (Cary 630, Agilent). The FTIR profiles of PET and PET-RB NPs were compared, see Figure S6. The FTIR profiles of both types of NPs showed the characteristic absorption bands of PET bulk polymer at 1715 cm^{-1} (C=O stretching), 1578 cm^{-1} (stretching of C=C in ring), 1505 cm^{-1} (in-plane bending of C–H in ring; stretching of C=C in ring), and 1240 cm^{-1} (C=O in-plane bending, C–C stretching, C(=O)–O stretching) [1]. Thus, the integration rhodamine kept the PET chemistry intact.

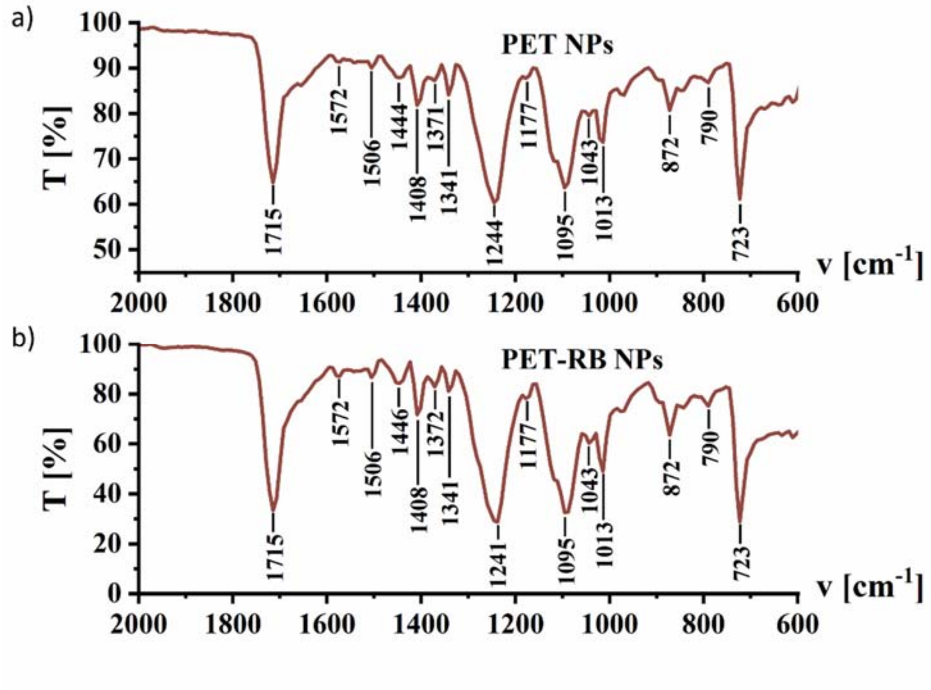

Figure S6. FTIR spectra of a) PET NPs and b) PET-RB NPs, showing the transmission T in dependence of the wavenumber v.

2.4) Characterization of optical properties

Fluorescence excitation and fluorescence emission spectra were collected in MilliQ water using a Fluorescence Spectrometer (Cary Eclipse, Agilent), see Figure S7.

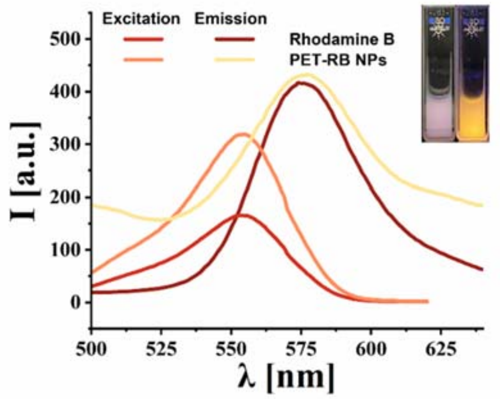

Figure S7. Excitation and emission spectra of aqueous solutions of PET-RB and PET NPs. The inset shows photographs of an PET-RB NP solution in visible light (left) and UV light (right).

2.5) Stability of properties in different media

The stability of the hydrodynamic diameter and the stability of RB fluorescence of the PET-RB NPs was probed by incubating the NPs in phosphate buffered saline (PBS), pH 7.4 or RPMI-1640 culture medium supplemented with 10% FBS and 1% P/S (see the cell culture part for details) for 120 h at 25 °C, while monitoring the hydrodynamic diameter and fluorescence intensity over time, see Figure S8. The PBS and RPMI-based media mimic tumor extracellular and cytoplasmic conditions [7, 8]. Data indicate stability of both properties (d_h, I) in the 2 different media.

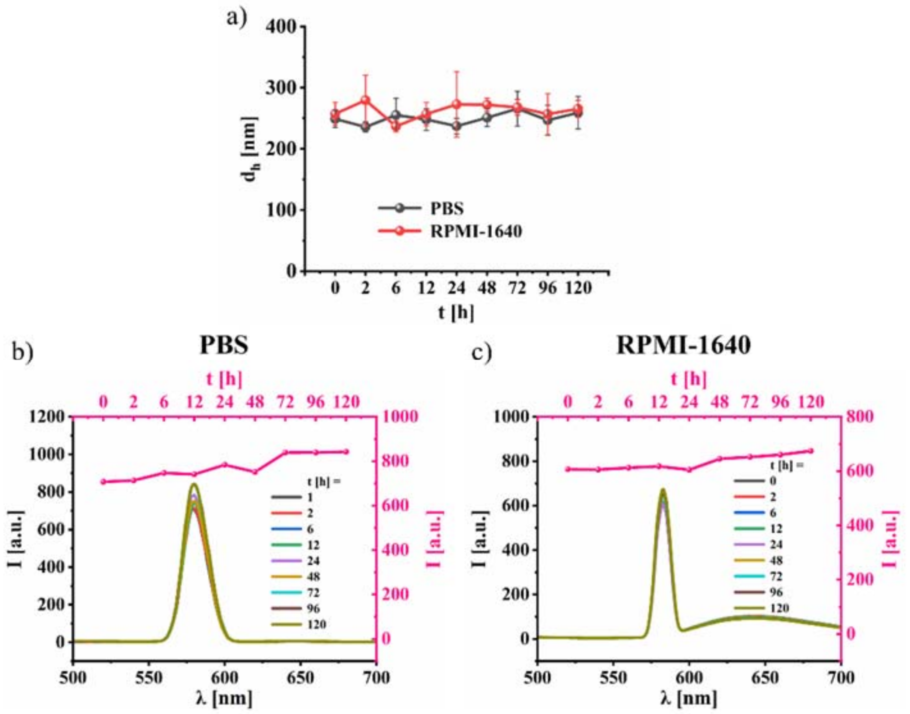

Figure S8. Time-dependent stability of PET-RB NPs, which have been incubated for the time t either in PBS (pH = 7.4) or RPMI-1640 culture medium supplemented with 10% FBS and 1% P/S. a) Hydrodynamic diameter d_h (for DLS intensity-weighted distribution) over incubation time t. b,c) Fluorescence spectra I(λ) at different times t (black axes) and fluorescence intensity versus time I(h).

2.6) Estimation of the amount of PETase per capsule

According to §1.3 for the PETase encapsulated 400 µL of 5 mg/mL PETase was used for the template core synthesis, corresponding to 0.4 mL·5 mg/mL = 1.25 mg PETase. After the core dissolution process and the encapsulation the capsules were dispersed in 1 mL water, resulting in PETase concentration of 1.25 mg/mL PETase. Note, that this is the theoretical upper limit, not considering losses due to inefficient PETase integration in the cores and losses in the washing steps. The amount of capsules in this stock solution was measured to be around 10^8 mL^{-1}. We can thus estimate the upper limit for the amount of PETase per capsule as 1.25 mg·mL^{-1}/10^8 mL^{-1} = $1.25 \cdot 10^{-8}$ mg = 12.5 pg.

It needs to be noted that this "upper limit" may be much higher as the real amount of proteins per capsules. Thus, a direct quantification would have been advantageous. There are several potential routes towards such quantification. i) The protein content in the supernatant is measured before and after encapsulation and the difference is assumed to correspond to the amount of encapsulated proteins. However, in case the encapsulation efficiency is low, this may lead to big errors. [9] ii) The amount of proteins in a solution with a known number of PETase@caps capsules could be determined by mass spectrometry.[10] However, protein quantification via mass spectrometry is often done by first enzymatically digesting the proteins, determining the content of protein fragments by mass spectrometry, and then by using a data base determining the amount of protein. Absolute quantification is not trivial and exceeded our available competence. iii) The amount of proteins can be determined with a colorimetric reaction, such as the Bradford assay.[11] Here the conceptual problem is due the encapsulations of the proteins by polymers. For the colorimetric detection a chemical reaction between reagents and the proteins are requires. The capsule walls may interfere with the diffusion of the reagents to the proteins. The capsules also scatter light, which may interfere with standard UV/vis absorption measurements. The capsules can be "opened", such as described in §3.2 in the SI for determining the enzymatic activity. However, also here the scattering of capsule fragments may interfere with absorption measurements, as the absorption of the polymer shell adds to the background. iv) In case of fluorescent of fluorescence- labelled proteins the amount of encapsulated proteins can be determined by their fluorescence in case a calibration curve which relates the fluorescence with the amount of proteins has been recorded before.[12]

The most promising approach to quantify the amount of encapsulated enzymes per capsule is using mass spectrometry, which however has not worked out in this study. This approach however will be attempted in future studies with an improved enzyme/nanoplastics system.

3) Degradation properties ("in test tube")

3.1) Degradation of PET by PETase
3.2) Degradation of PET by encapsulated PETase

3.1) Degradation of PET by PETase

Following published work [13, 14], the enzymatic hydrolysis of PET NPs was monitored by 2-hydroxyterephthalate (HOTP, λ_{ex} = 328 nm and λ_{em}= 421 nm), a fluorophore produced in situ after radical hydroxylation of terephthalic acid (TPA) by the Fenton reaction. 50 µL of PET NP (C_{NP} = 7 mg/mL) solution was added as the substrate into a microcentrifuge tube with 1 ml phosphate buffer (PB, Na_2HPO_4 - KH_2PO_4, 1/15 M, pH 8), followed by 25 µL of PETase (2 mg/mL). The mixture solution was incubated at room temperature for 48 h in a plate shaker (200 rpm, orbital shaker KS 250B, IKA). Enzymatic degradation of the PET NPs leads to the formation of TPA and ethyleneglycol (EG), see Figure S9. The solution was centrifuged at 3000 rpm for 5 min in order to precipitate the remaining PET NPs. 30 µL of the supernatant containing the formed TPA was added per well into 96-well black microliter plates, followed by the addition of 100 µL PB solution (1/15 M, pH 8), 30 µL of H_2O_2 (2% v/v), 20 µL of EDTA (3 mm), and 20 µL of $FeSO_4{\cdot}7H_2O$ (3 mM) per well (i.e. a total volume of 200 µL per well). The above solution was incubated for 25 min at room temperature to trigger HOTP formation, see Figure S9. The reaction was stopped by adding 50 µL of 2 M H_2SO_4 solution and the fluorescence (cf. Figure S10) was analyzed on a FLUOstar Omega microplate reader (BMG LABTECH, Germany) with a 460 nm emission filter using the Omega 4.01 R2 software.

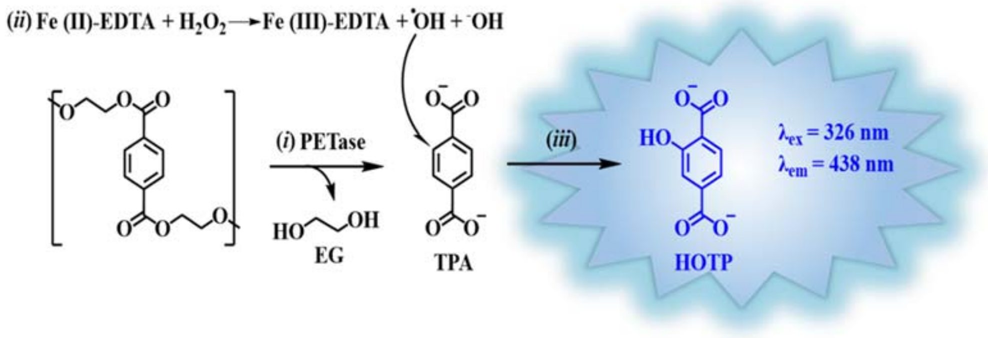

Figure S9. (A) Fluorimetric detection of terephthalate (TPA) as the PET NPs. (i) Enzymatic hydrolysis of PET yields TPA and EG as monomeric products. (ii) The formation of hydroxyl radicals mediated by the Fe(II)-EDTA complex. (iii) The hydroxylation of terephthalate to fluorescent 2-HOTP in the presence of hydroxyl radicals. The excitation and emission maxima of 2-HOTP are 326 nm and 438 nm, respectively.

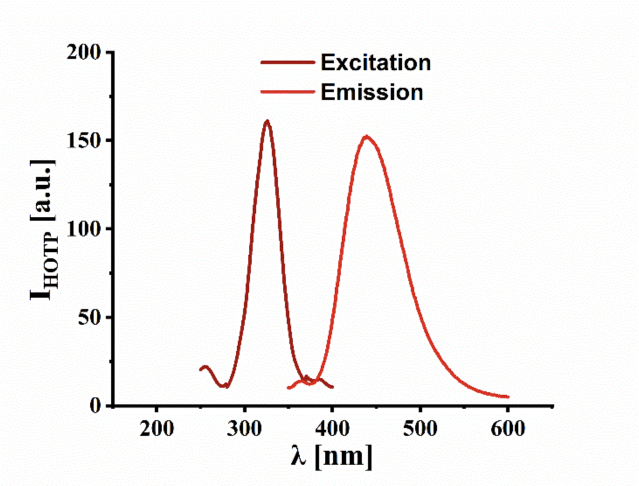

Figure S10. Excitation and emission spectra of HOTP.

The assay was performed for PET NPs an PET-RB NPs in the presence of PETase and as control without PETase. The results of the fluorescence read-out are presented in Figure S11 (and in different presentation in Figure 2a). The show, that after 12 h there is a saturation in the HOTP fluorescence, i.e. no further increase of the degradation products of PET. Without PETase there is on production of HOTP.

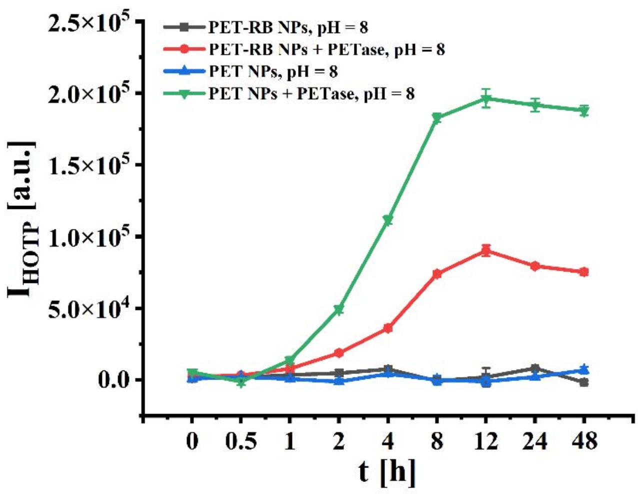

Figure S11. HOTP fluorescence I_{HOTP} of PET-RB NPs and PET NPs incubated with or without PETase at pH = 8. This is the same data as shown in Figure 2a.

For details about the kinetics of this enzymatic degradation assay we refer to the original work [13, 14]. The HOTP assay was only used in the following to probe for pH dependence (Figure S12) and the role of encapsulation (Figure S13). For experiments with cells the degradation was determined as loss of intracellular RB fluorescence (see §6 and §7), and not with the HOTP assay. Therefore, limitations of this assay as described in the following were not fully investigated, as they were not relevant for the assays involving cells.

There are intrinsic limitations to the HOTP assay. Detection of HOTP is made in the supernatant, after having pelleted residual PET NPs. Thus, strictly speaking, saturation in HOTP fluorescence (as in Figure S11 after 8 - 12 h) only indicates that there is no longer degradation of the PET NPs, but it does not

directly proof that all the PET NPs have been fully degraded. Hypothetically it also could be that the PET NPs can degrade on their initial surface and not in their volume, which would result in HOTP fluorescence saturation after the surface shell has been degraded while the volume of the PET NPs remains intact. This scenario however does not seem likely. Alternatively upon degradation their might be an inactivation of the PETase, which also would lead to a saturation of HOTP fluorescence, while not all PET NPs have been degraded. Also this scenario does not seem likely. Still, the assay shown in Figure S11 does not proof that PETase can fully degrade the PET NPs.

Also the concentration dependence of PETase on the degradation was not explored. The conditions in Figure S11 were chosen that the PETase concentration is high enough to warrant to degrade the PET NPs to a point that after around half a day there is no further production of degradation product (HOTP). As after encapsulation of PETase the precise PETase concentration is not known such concentration dependence would also be hard to apply for the here presented encapsulated PETase (see the discussion after Figure S13).

In order to probe for the pH-dependence of the PET degradation by PETase the above described experiment was repeated at different pH values. For this 50 µL of PET NP (7 mg/mL) solution and 25 µL of PETase (2 mg/mL) was mixed with 1 mL PB solution of different pH and the analysis was carried out as described above. PB solutions were mixed from two stock solutions, Na_2HPO_4 (1/15 M) and KH_2PO_4 (1/15 M) in different volume ratios as follows:

- pH ≈ 4: volume ration 0:10; i.e. only KH_2PO_4; as the initial value of KH_2PO_4 (1/15 M) is 4.5 hydrochloric acid was added to adjust the pH to 4
- pH ≈ 5 (here: pH = 4.92): volume ratio 0.1:9.9
- pH ≈ 6 (here: pH = 5.91): volume ratio 1.0:9.0
- pH ≈ 7 (here: pH = 6.98): volume ratio 6.0:4.0
- pH ≈ 8 (here: pH = 8.0): volume ratio 9.5:0.5

Results are shown in Figure S12 and indicate that at pH = 4 there is basically no enzymatic activity, i.e. degradation of PET.

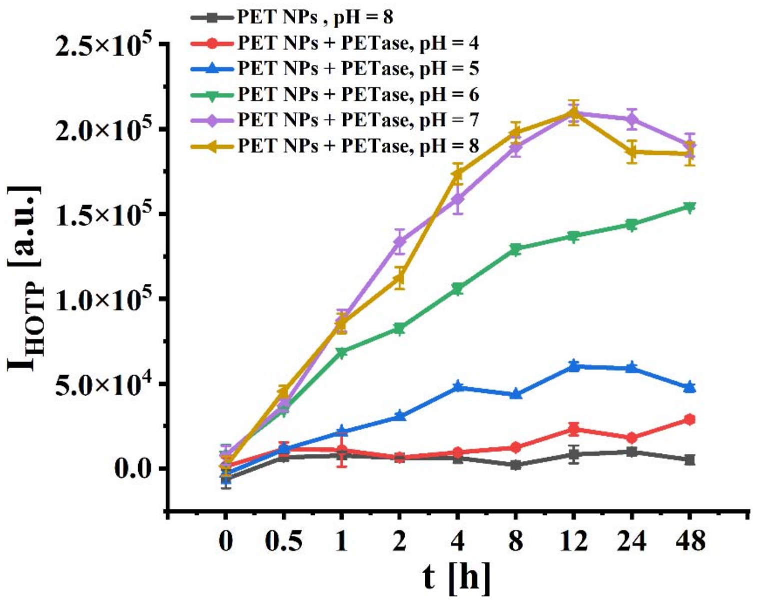

Figure S12. HOTP fluorescence I_{HOTP} of PET NPs incubated with or without PETase at different pH values. This is the same data as shown in Figure 2b.

Comparison of Figure S11 and Figure S12 allows for a control of reproducibility. The green curve in Figure S11 and the brown curve in Figure S12 show data recorded for the same condition (PET NPs + PETase, pH = 8), but carried out in independent experimental series. The data of both recordings are compatible.

3.2) Degradation of PET by encapsulated PETase

The same assay as described in §3.1 was also carried out for encapsulated PETase (with $(DEXS/pARG)_4$ and $(DEXS/pARG)_2/(DEXS/PEI)_2$ capsule shell geometry). In order to allow access of the PET NPs to the encapsulated PETase the capsules had to be opened. Prior to the assay, the capsules were subjected to sonication (SONOREX DIGITEC DT 102H; Bandelin, Germany; power:120-480 W, frequency: 35 kHz) in a cold water bath for 10 min to facilitate their disruption and the release of the enzyme. The the degradation assay was carried out as described in §3.1. Results are shown in Figure 2c and Figure S13 (same data as in Figure 2d).

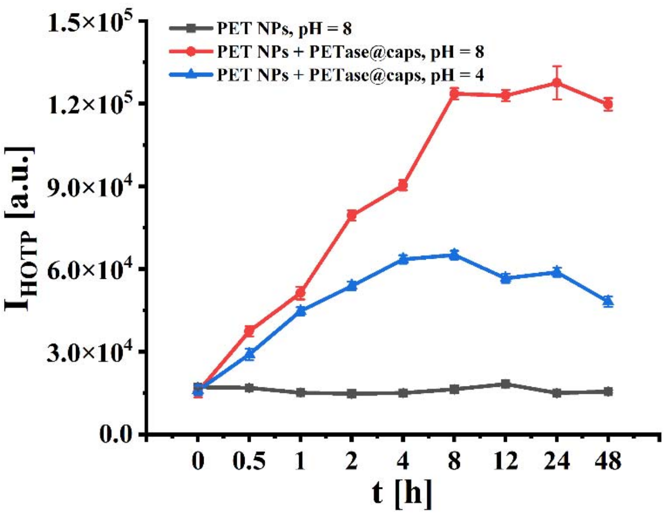

Figure S13. HOTP fluorescence I_{HOTP} of PET NPs incubated with or without encapsulated PETase ($(DEXS/pARG)_2/(DEXS/PEI)_2$ shell geometry) at different pH values. This is the same data as shown in Figure 2d.

We note that the results shown in Figure S12 and Figure S13 only can be compared with restrictions. For a fair comparison the amount of enzyme in the solution of encapsulated PETase in Figure S13 should correspond to the amount of free PETase (25 μL of PETase (2 mg/mL), corresponding to 0.025 mL·2mg/mL = 50 μg) in Figure S12. However, the enzyme concentration in case of the encapsulated enzyme can only be estimated. According to §1.3 for the PETase encapsulated 400 μL of 5 mg/mL PETase was used for the template core synthesis, corresponding to 0.4 mL·5 mg/mL = 1.25 mg PETase. After the core dissolution process and the encapsulation the capsules were dispersed in 1 mL water, resulting in PETase concentration of 1.25 mg/mL PETase (see also the similar discussion in §2.6). Note, that this is the theoretical upper limit, not condidering losses due to inefficient PETase integration in

the cores and losses in the washing steps. The amount of capsules in this stock solution was measured to be around 10^8 mL^{-1}. Instead of 25 μL of PETase (2 mg/mL), corresponding to 50 μg PETase in the case of the free PETase (see §3.1) for the encapsulated PETase ($(DEXS/pARG)_2/(DEXS/PEI)_2$ shell geometry) 100 μL of 1.25 mg/mL encapsulated PETase were added, corresponding to 1.25 mg/mL·0.1 mL = 125 μg. Thus, for the data shown in Figure S13 around the double amount of PETase is present than for the data shown in Figure S12, in case the losses in encapsulation of PETase are neglected, which however is not realistic. Thus the data of both figures need to be compared with care.

4) Basic cell culture procedures

4.1) Cell culture techniques
4.2) Cell viability assays

4.1) Cell culture techniques

Human cervical carcinoma cells (HeLa cells) were cultured in Dulbecco's Modified Eagles Medium (DMEM, #11965092, ThermoFisher Scientific) with 4.5 g/L glucose supplemented with 10% fetal bovine serum (FBS, Biochrom, Germany, #S0615), 1% penicillin/streptomycin (P/S, #15070063, Thermo Fisher Scientific) at 5% CO_2 and 37 °C. The cell viability experiments (§4.2) and the degradation experiments (§6, §7) were carried out in this serum-supplemented medium. In the colocalization experiments (§5) serum-free culture medium was used for the incubation with LysoTracker.

4.2) Cell viability assays

Cell viability assays of PET-RB NPs and PETase@caps ($(DEXS/pARG)_2/(DEXS/PEI)_2$ shell geometry) were assessed in terms of metabolic activity as measured via a resazurin reduction assay [14]. HeLa cells were seeded in black 96-well plates (Corning® 3603, USA, 0.32 cm^2 seeding area per well) at an initial density of 5,000 cells per well in 100 µL medium per well at 37 °C and 5% CO_2 in an incubator. After overnight incubation, the medium was replaced with medium with either PET-RB NPs at concentration C_{NP} or PETase@caps with $N_{caps/cell}$ capsules added per cell. As control cells without added PE-RB NPs or PETase@caps were used. After 24 h or 48 h of exposure, the cells in each well were washed three times with PBS. Then, 200 µL sterile filtered resazurin solution (25 µg/mL in medium) was added to the corresponding wells and allowed to react at 37 ^{o}C, 5% CO_2 for 3 h. After incubation, the fluorescence due to the reduction of resazurin upon metabolic activity (excitation λ_{ex} = 544 nm, emission λ_{em} = 590 nm, cut-off at 570 nm) was measured on a FLUOstar Omega microplate reader (BMG LABTECH, Germany) with Omega 4.01 R2 software. The cell viability V was calculated as fluorescence signal of the cells exposed to PET-RB NPs or PETase@caps normalized to the fluorescence signal of the control cells which had not been exposed to PET-RB NPs or PETase@caps. Results are shown in Figure S14 and Figure S15. In the investigated concentration range there was not reduction in cell viability upon the presence of PET-RB NPs. However, at high capsule exposure concentrations (which are higher than used for the following experiments) a reduction in cell viability was found [15]. Such high concentrations are artificial and would not occur in any applications scenario. At such high concentrations the cells surface is virtually covered with capsules [15]. The reduction in viability thus is not related to intrinsic toxicity of the capsules, but to the clogging of the cells with capsules, which would occur also for other intrinsically non-toxic particles. Studies using more realistic conditions show that the capsules per se (in case case not loaded with toxic components) are immunocompatible [10].

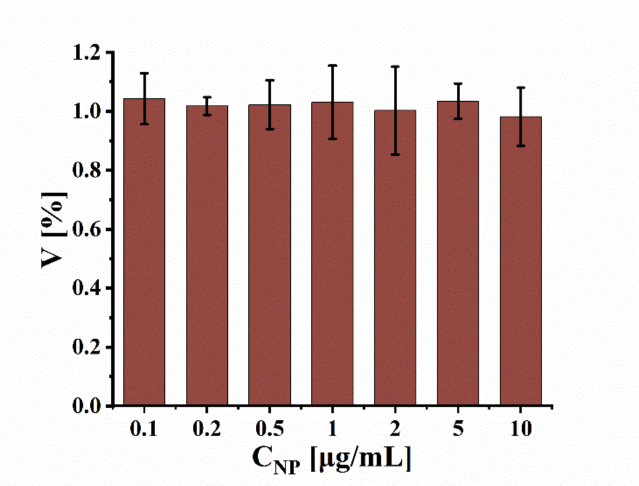

Figure S14. Cell viability V in dependence of the exposure concentration C_{NP} of PET-RB NPs (24 h exposure time). Data are shown as mean values ± standard deviations (SD); n = 5 repeats.

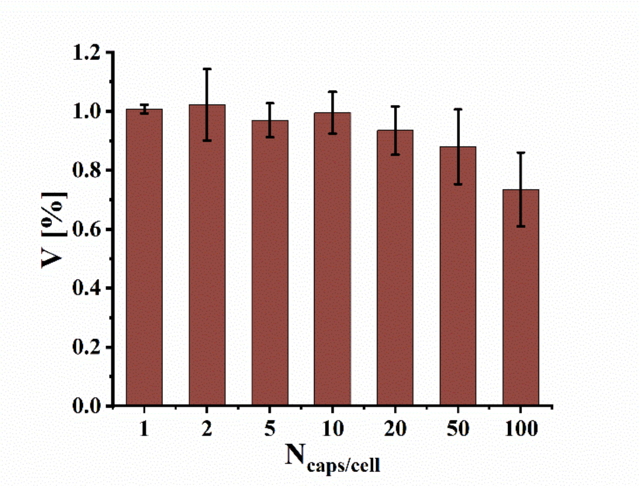

Figure S15. Cell viability V in dependence of the number of capsules N_{caps} which have been added per cell (48 h exposure time). Data are shown as mean values ± standard deviations (SD); n = 5 repeats.

5) Colocalization experiments

In order to verify whether upon delivery of PETase@caps to cells via endocytosis, and exposure of cells to PET NPs, both, PETase and PET NPs co-localize, which would be necessary for the degradation of PET by PETase. As PETase is nonfluorescent, instead of PETase@caps we used BSA-FITC@caps in order to track the intracellular localization of proteins delivered with capsules. FITC fluorescence thus indicates the location of BSA as model protein. The capsules were in ((DEXS/pARG)$_2$/(DEXS/PEI)$_2$ shell geometry. In order to visualized the PET NPs we took PET-RB NPs which could be identified by the RB fluorescence. Additionally, in some experiments the lysosomes were stained with LysoTracker™ Deep Red (Invitrogen™ L12492, Germany).

For colocalization imaging 100,000 Hela cells in 2 mL of medium supplemented with 10% FBS were seeded in per µ-Dish (#81156, ibidi; bottom area: 3.5 cm^2). The cells were kept overnight at 5% CO_2 and 37 °C. Afterward, cells were incubated with PET-RB NPs at a concentration of C_{NP} = 5 µg/mL overnight. On the third day, the cells were incubated with BSA-FITC@caps at a concentration of $N_{caps/cell}$ = 1.5 capsules per seeded cell overnight. On the fourth day, i.e. after 24 h incubation of cells with BSA-FITC@caps, the lysosomes of cells were optionally stained. For this, cells were washed three times with PBS and incubated with 1 mL of serum-free medium (this is the only serum-free incubation step) containing 50 nM LysoTracker™ Deep Red staining solution for 30 min at 37 °C and 5% CO_2 in an incubator. After this cells were washed three times with PBS and 2 mL of culture medium with 10% FBS was added. In case no lysosomal staining was carried out the medium was directly replaced with 2 mL of fresh cell culture medium supplemented with 10% FBS. Then, cells were imaged using an upright confocal laser scanning microscope (LSM880 system, Zeiss). Images were acquired by the software ZEN 2.3 (blue edition, operation software for LSM880). The following settings were used: BSA-FITC@caps fluorescence: λ_{ex} = 488 nm, λ_{em} = 517 nm; PET-RB NP fluorescence: λ_{ex} = 561 nm, λ_{em} = 577 nm; Lyso-tracker fluorescence: λ_{ex} = 633 nm, λ_{em} = 688 nm. Images are shown in Figure S16, Figure S17, Figure S18, and Figure 3.

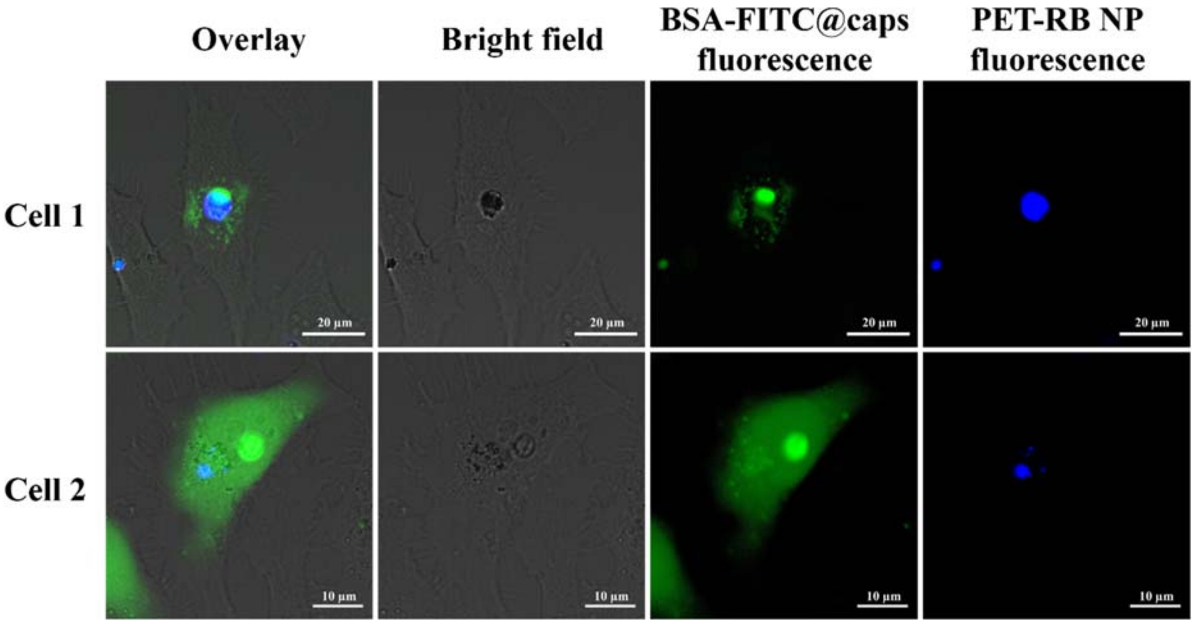

Figure S16. Fluorescence images of 2 cells which had been exposed to PET-RB NPs and BSA-FITC@caps. The image shows, that there is some release of BSA-FITC from BSA-FITC@caps and the surrounding

endosome/lysosome into the cytosol. Note that there is some overexposure in the fluorescence images in order to show also the part of the BSA-FITC that has been released to the cytosol. The scale bars correspond to 10 µm. z-scans for cell 1 and cell 2 are shown in Supplementary Movie M1 and Supplementary Movie M2, respectively.

Movie M1 and Movie M2: z-scans for cell 1 and cell 2 as shown in Figure S16. The movies are embedded in the pdf File of the Supporting Information.

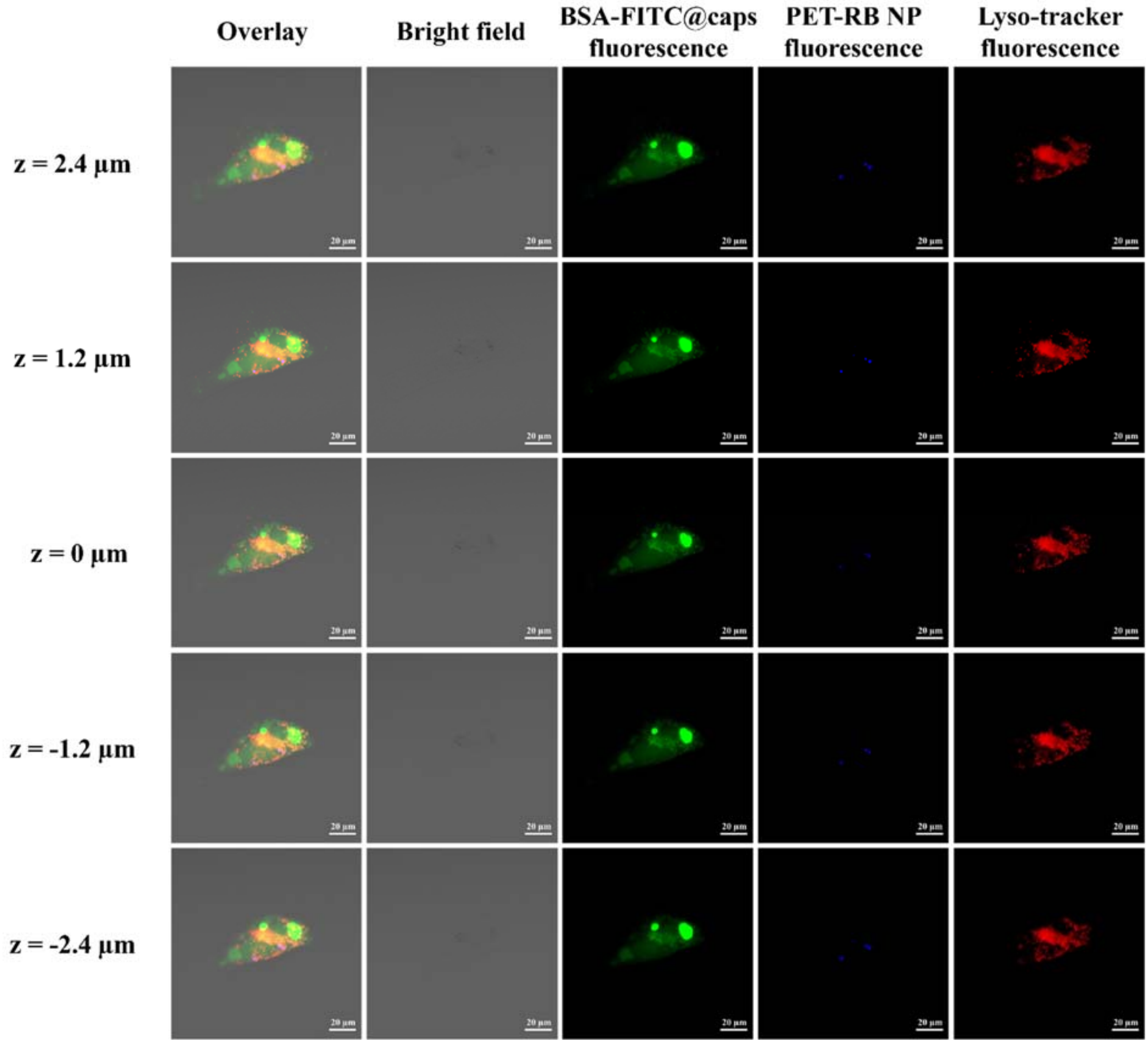

Figure S17. z-scan fluorescence images of 1 cell which had been exposed to PET-RB NPs and BSA-FITC@caps and which had been stained with LysoTracker™ Deep Red. Note that there is some overexposure in the FITC fluorescence channel in order to show also the part of the BSA-FITC that has been released to the cytosol. The images suggest co-localization of the PET-RB NPs with lysosomes. For BSA-FITC it is hard to see colocalization due to overexposure. The scale bars corresponds to 20 µm. A corresponding z-scan is shown in Supplementary Movie M3. z = 0 µm is the best focal plane; step width Δz = 1.2 µm.

Movie M3: z-scan for the cell shown in Figure S17. The movie is embedded in the pdf File of the Supporting Information.

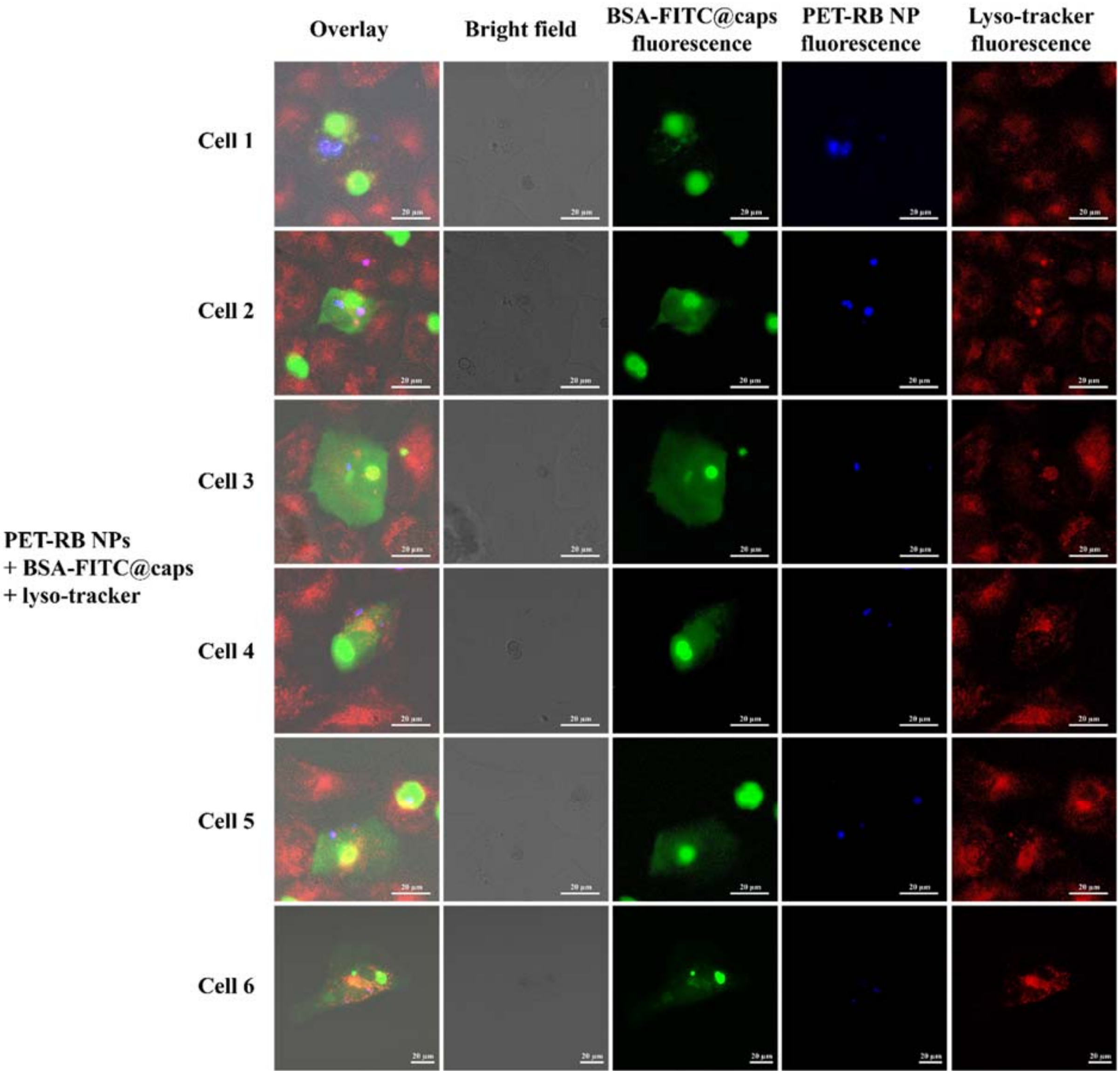

Figure S18. Fluorescence images of 6 cells which had been exposed to PET-RB NPs and BSA-FITC@caps and which had been stained with LysoTracker™ Deep Red. Note that there is some overexposure in the FITC fluorescence channel in order to show also the part of the BSA-FITC that has been released to the cytosol. In some of the cells colocalization of BSA-FITC@caps with lysosomes can be seen. The scale bars corresponds to 20 µm. These images have been taken at similar conditions as the first line of images in Figure 3.

6) Intracellular degradation (multiple cells)

To probe for PET degradation by endocytosed PETase@caps cell were first incubated with PET-RB NPs, later with PETase@caps, and then after incubation and rinsing the RB-fluorescence originating from PET-RB NP fragments still remaining in cells was determined with a fluorescence microplate reader.

In detail, HeLa cells were seeded in black 96-well plates (Corning® 3603, USA, 0.32 cm^2 seeding area per well) at an initial seeding density of 5,000 cells per well. Cells were incubated for 48 h at 37 °C and 5% CO_2 in 100 µL of serum-supplemented medium per well in an incubator. Afterwards, PET-RB NPs were added leading to a final concentration of C_{NP} = 5 µg/mL, and incubated continued overnight. On the third day, residual non-internalized PET-RB NPs were removed by aspiration, and cells were incubated in fresh medium with PETase@caps at a density of 1.5 capsules added per initially seeded cell. According to §2.6 the upper estimated limit of PETase per capsule is 12.5 pg. After incubation for the time t, cells were washed 3 times with PBS. The the RB fluorescence I_{RB} of each well was recorded with microplate reader (BMG LABTECH, Germany; λ_{ex} = 544 nm, λ_{em} = 590 nm, 10 nm bandpass filter). As control the same experiment but without addition of PETase@caps was performed. Data are shown in Figure 4 and Figure S19.

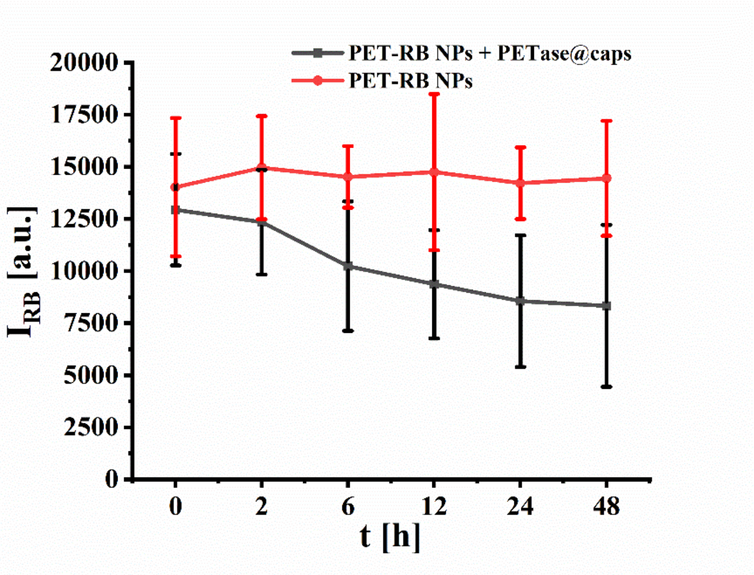

Figure S19. Fluorescence Intensity I_{RB} of PET-RB NPs in cells after co-incubation with PETase@caps ($(DEXS/pARG)_2/(DEXS/PEI)_2$ shell geometry) for the time t. No PETase@caps was used for the control. Data shown are the mean values recorded on n = 20 wells ± the respective standard deviations. Similar data as recorded in a separate experiment are presented in Figure 4.

7) Intracellular degradation (single cells)

7.1) Experimental procedure and data processing
7.2) Results of cellular exposure to PET-RB NPs and PETase@caps
7.3) Results of cellular exposure to PET-RB NPs only
7.4) Results of cellular exposure to PETase@case only

7.1) Experimental procedure and data processing

The degradation of PET-RB NPs upon the presence of PETase@caps was monitored with fluorescence microscopy at sing cell level. Incubation with PET-RB NPs only and PETase@caps only served as control.

For one experiment 100,000 Hela cells in 2 mL of culture medium supplemented with 10% FBS were seeded in a grid-500 µ-Dish (#80156, ibidi; seeding are area: 3.5 cm^2). The cells were grown overnight at 5% CO_2 and 37 °C. The next day, the cells were incubated with PET-RB NPs at a concentration of C_{NP} = 5 µg/mL at 37 °C and 5% CO_2 in an incubator. After culture overnight, on the third day, residual non-internalized PET-RB NPs were removed by aspiration and the cells were incubated in 2 mL of fresh medium with PETase@caps ($(DEXS/pARG)_2/(DEXS/PEI)_2$ shell geometry) at a density of 1.5 capsules added per initially seeded cell. Cells were then kept for the time t in culture at 37 °C and 5% CO_2 in an incubator. After incubation, the cells were washed three times with PBS and 2 mL of culture medium supplemented with 10% FBS was added. Then, the cells were directly imaged by an upright confocal laser scanning microscope (LSM880 system, Zeiss). 5 different incubation time points t (2 h, 6 h, 12 h, 24 h and 48 h) were analyzed. During imaging the temperature was controlled to be 37 °C, but there was no CO_2 control. The following settings were used: bright field, PET-RB NP fluorescence: λ_{ex} = 561 nm, λ_{em} = 577 nm. The images were analyzed by the software ZEN 2.3 (blue edition, operation software for LSM880). An example for a recorded data set is shown in Figure S20.

Other example of cells which have been exposed to first PET-RB NPs and then to PETase@caps are presented in §7.2. Data for cells exposed to PET-RB NPs only (here instead of the addition of PETase@caps only medium was added) are given in §7.3. Data for cells exposed to PETase@caps only (here C_{NP} = 0, the regions of interested (see below) were selected at random areas in the cells) are given in §7.4.

We note an inconsistency in the definition of the incubation times t. For the experiments involving PETase@caps (i.e. presence of PET-RB NPs + PETase@caps or PETase@caps only) the incubation time starts (i.e. t = 0) when PETase@caps has been added. In the case no PETase@caps has been added (i.e. PET-RB NPs only) t = 0 was defined when PET-RB NPs were added. It would have been better to define also in this case t = 0 after 1 day after addition of PET-RB NPs, which is would then have the similar starting point as in the case of PET-RB NPs + PETase@caps. Looking at the data of Figure 5c this inconsistency however has no practical importance.

Note that for the images showing single cells (Figure S20 - S52 no lysosomal staining was performed. However, for the overview images shown in Figure 5a in the main article, the lysosomes were stained before imaging. For this, after the incubation time t the cells were washed three times with PBS and were then incubated with 1 mL of serum-free medium containing 50 nM LysoTracker™ Deep Red

(Invitrogen™ L12492, Germany) staining solution for 30 min at 37 °C and 5% CO_2 in an incubator. The protocol was then continues as described above with rinsing and imaging.

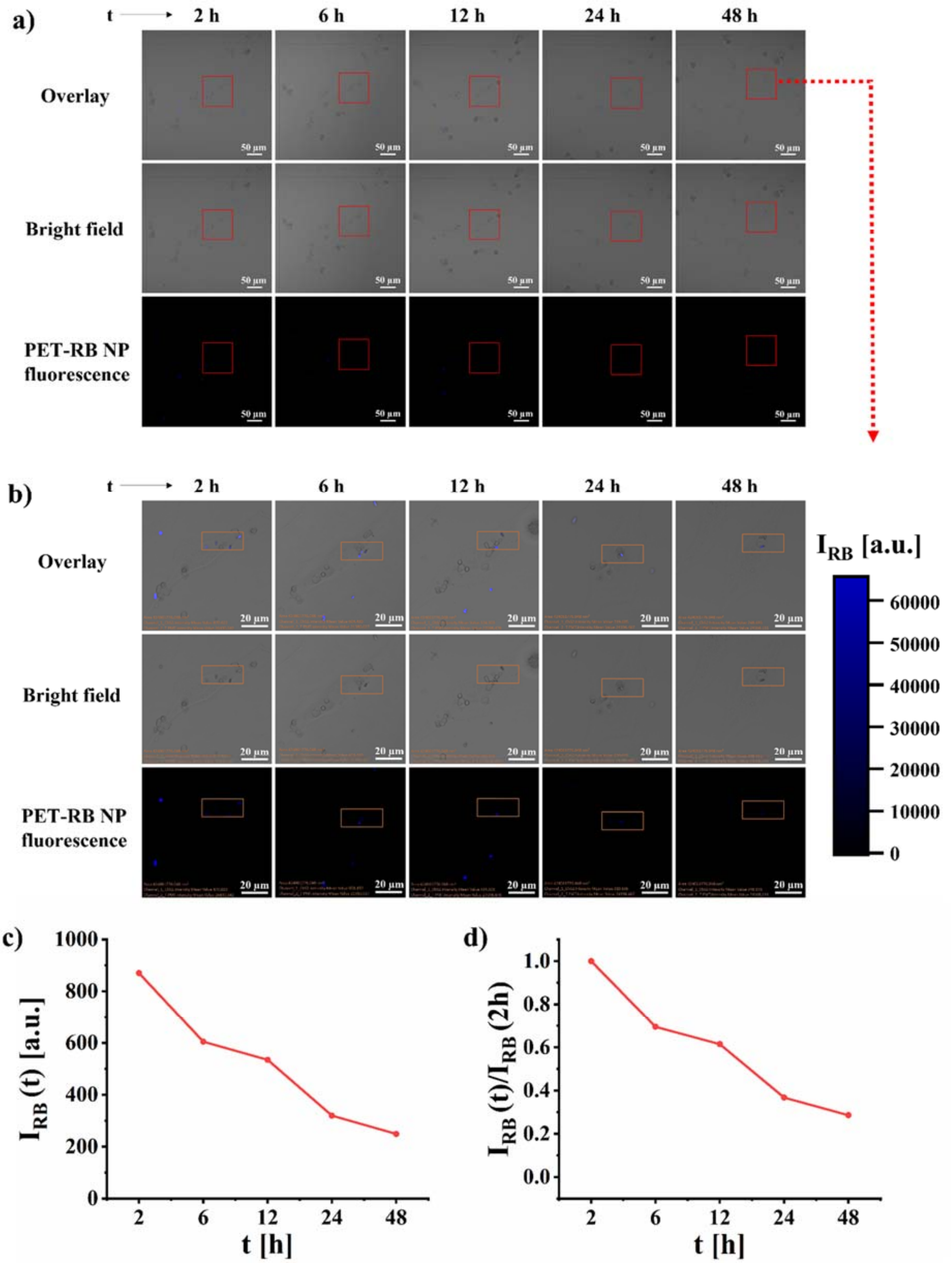

Figure S20. Example #1 of PET-RB NPs +PETase@caps. a) Cells have been incubated with PET-RB NPs and one day after also with PETase@caps. Images (bright field and RB fluorescence) were recorded at different time points t after after PETase@caps has been added. b) For the evaluation a region where

PET-RB NPs are visible in a) as indicated by the red box was magnified and is displayed in b). The scale bars correspond to 50 µm. b) Magnified areas from a). The scale bars correspond to 20 µm. An area around PET-RB NPs was selected (orange box) and this area was moved over time according to the movement of the respective cell. The RB fluorescence I_{RB} in this area was determined for the different time points. c) RB fluorescence I_{RB} of the selected area in b) plotted over time t. d) Data from c), normalized to I_{RB}(2 h).

The workflow for the data evaluation with the example of Figure S20 is presented in Figure S21. First, a "rough region of interest" (i.e. where fluorescence of intracellular PET-RB NPs is visible) was selected, see the red boxes in Figure S20a. In the "rough region of interest" a more refined region of interest was selected see the orange boxes in Figure S20b. The average intensities in the orange boxes were determined with the software ZEN 2.3 SP1 (blue edition). For this first the region of interest ROI was selected by manually delineating the area including intracellular PET-RB NPs by “Draw rectangle”, see Figure 20a. The software then calculated the average mean signal intensity in the selected rectangular orange box, see Figure 20b, here for the image at time point t = 2 h. In this example the area or the orange box was A_{ROI} = 424061776.048 nm^2. The mean fluorescence intensity in the RB channel was I_{RB} = 870.22. The mean intensity in the bright field channel (which is not used) was I_{bright_field} = 26430.542. The same procedure was then repeated for the images at different time points. In Figure 20c the image for the time point t = 6 h is shown. In order to obtain comparable results the area of the orange box (ROI) needs to be the same size in all images. As cells move the location of the ROI however can be shifted according to the position of the cell. The area or the orange box in Figure 20c was A_{ROI} = 424061776.048 nm^2. The mean fluorescence intensity in the RB channel was I_{RB} = 604.430. The mean intensity in the bright field channel (which is not used) was I_{bright_field} = 21380.027. The same procedure was then repeated for all time points of one experimental series, obtaining the I_{RB} data for all time points. For each series the time dependence of the mean fluorescence in the ROI of the RB channel was then plotted over time, see Figure S20c. The data were also normalized to the intensity value after 2 h (I_{RB}(2 h)), see Figure S20d.

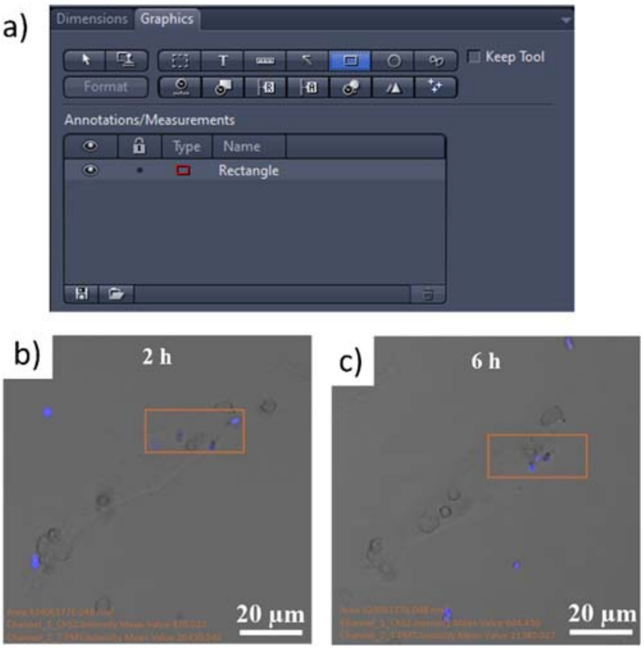

Figure S21. a) Procedure to manually select a ROI with the ZEN software. b, c) ROIs selected for the time points t = 2 h and t = 6 h (orange boxes). The area of the orange boxed (A_{ROI}), the mean fluorescence intensity in the RB channel (I_{RB}) and the mean signal intensity in the bright field channel (I_{bright_field}) were determined by the software.

The same procedure was repeated for all the different experimental series and the data are presented in §7.2 - §7.4.

7.2) Results of cellular exposure to PET-RB NPs and PETase@caps

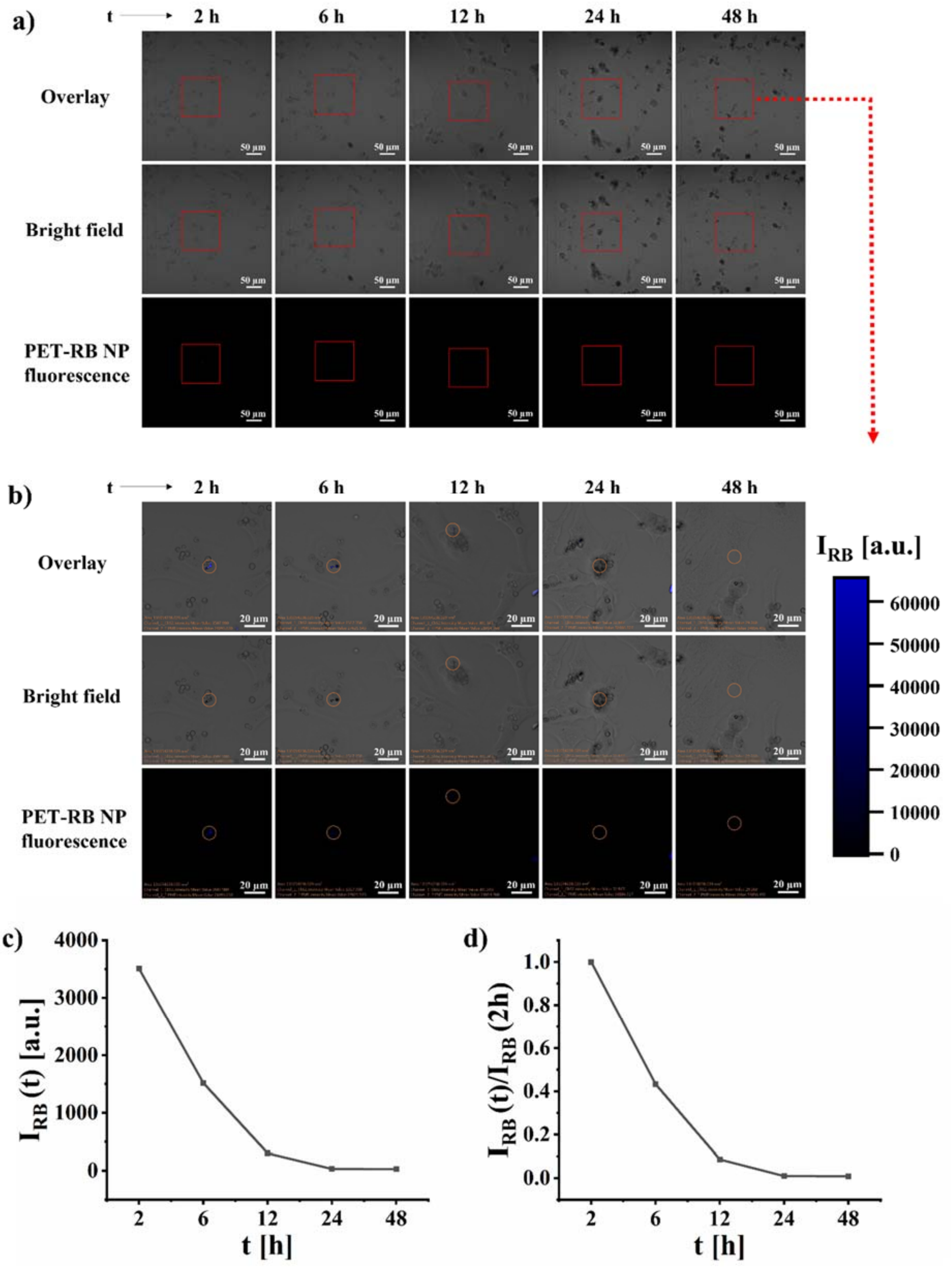

Figure S22. Example #2 of PET-RB NPs +PETase@caps. For further explanation see the legend of Figure S20.

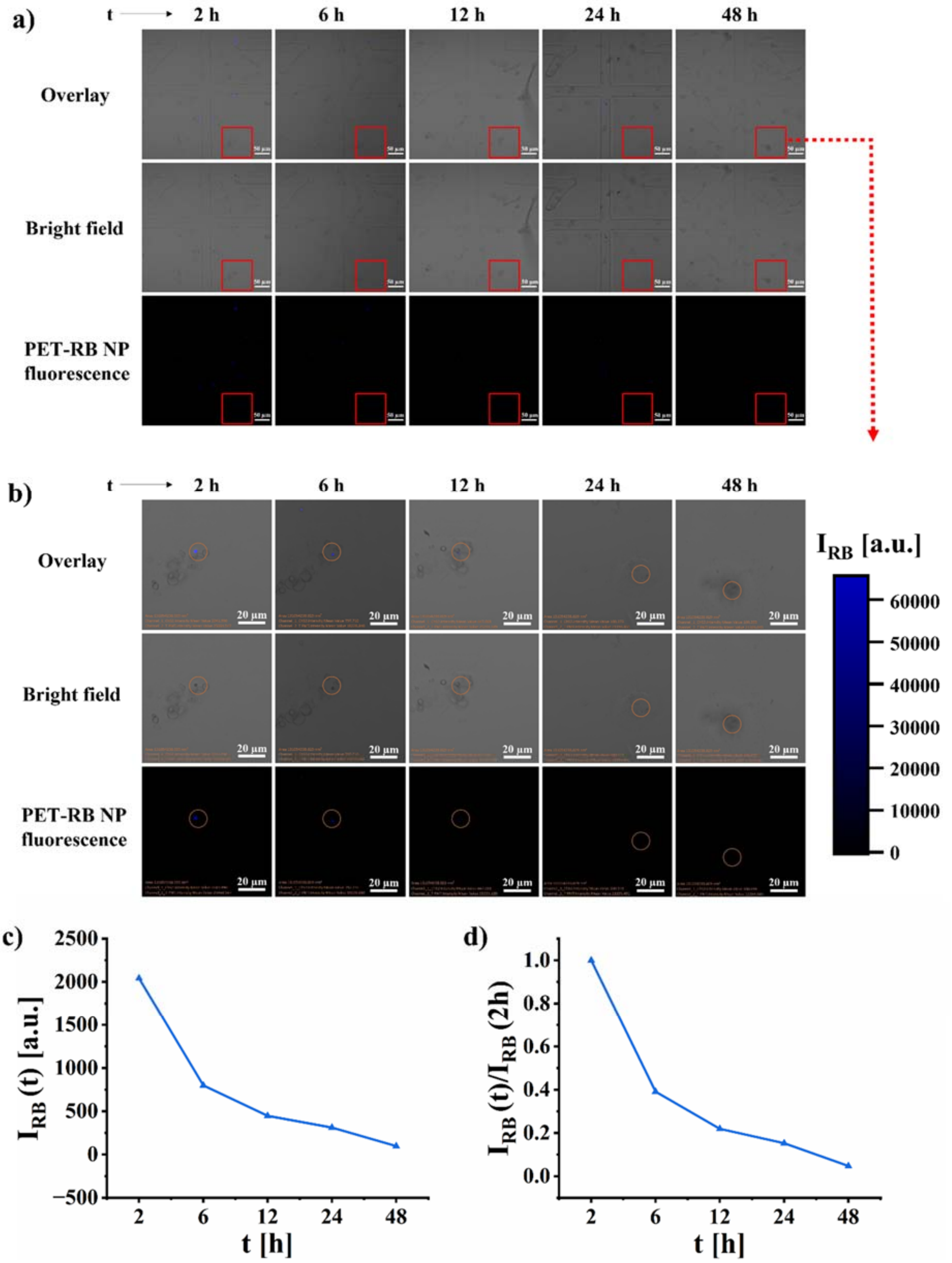

Figure S23. Example #3 of PET-RB NPs +PETase@caps. For further explanation see the legend of Figure S20.

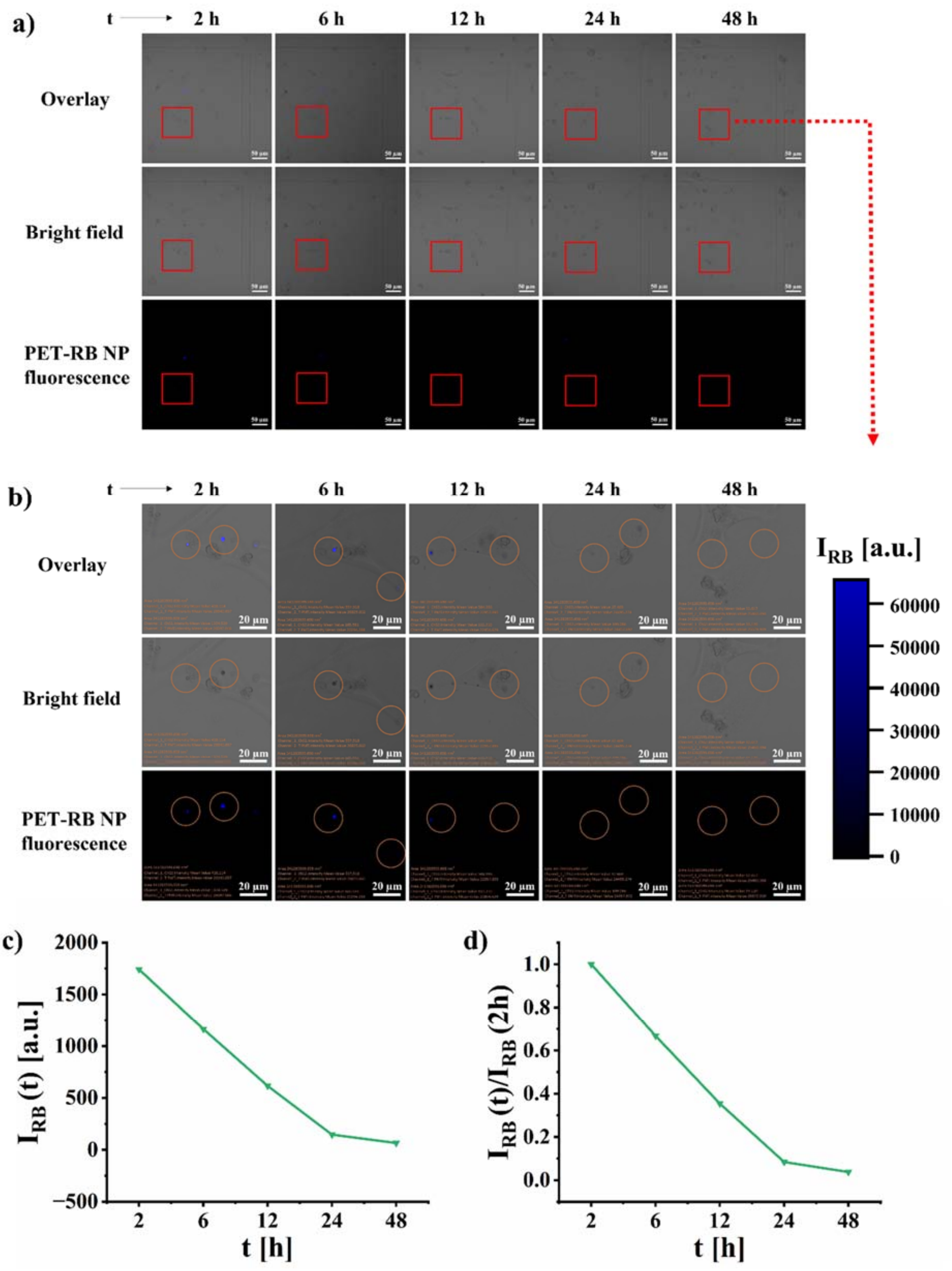

Figure S24. Example #4 of PET-RB NPs +PETase@caps. For further explanation see the legend of Figure S20.

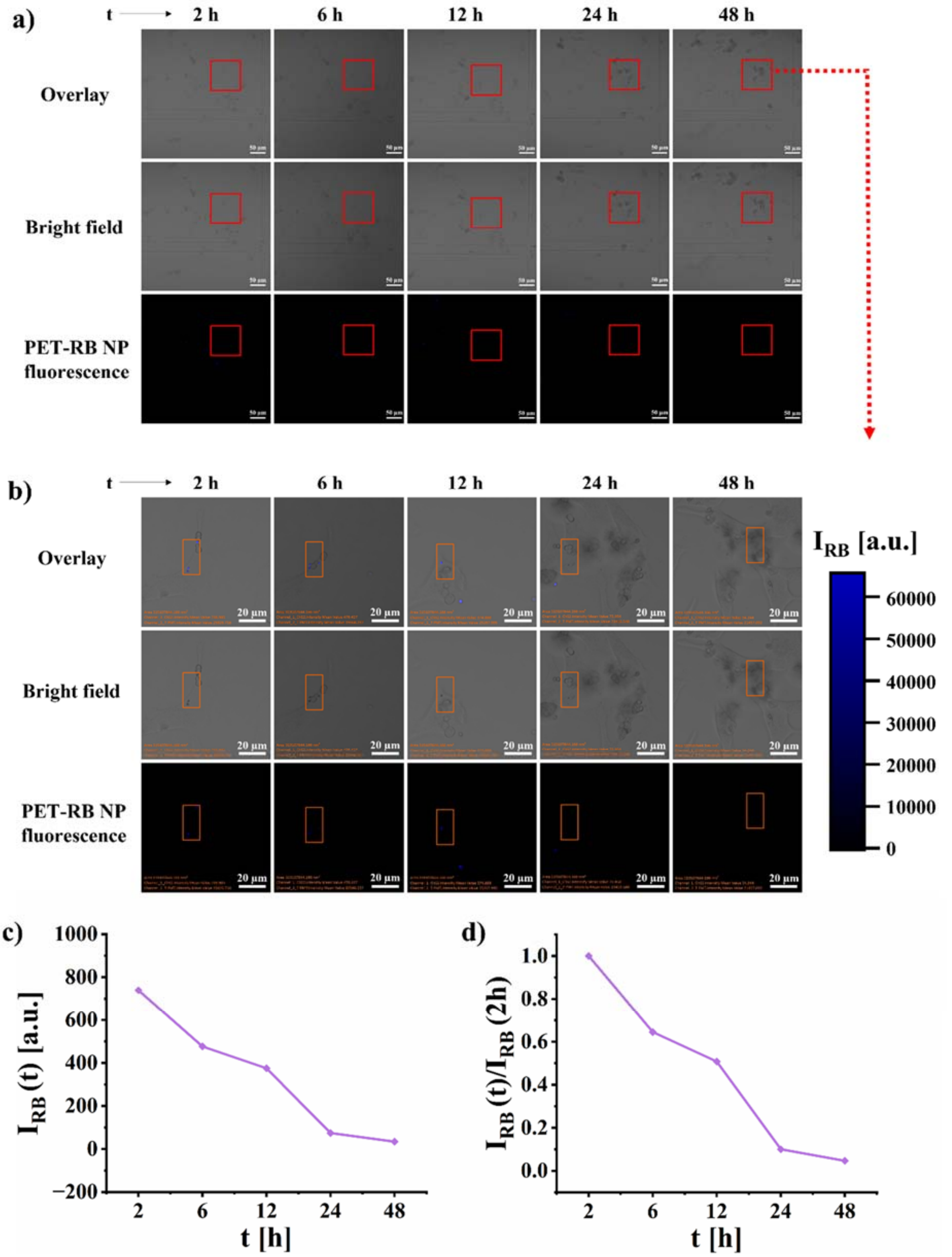

Figure S25. Example #5 of PET-RB NPs +PETase@caps. For further explanation see the legend of Figure S20.

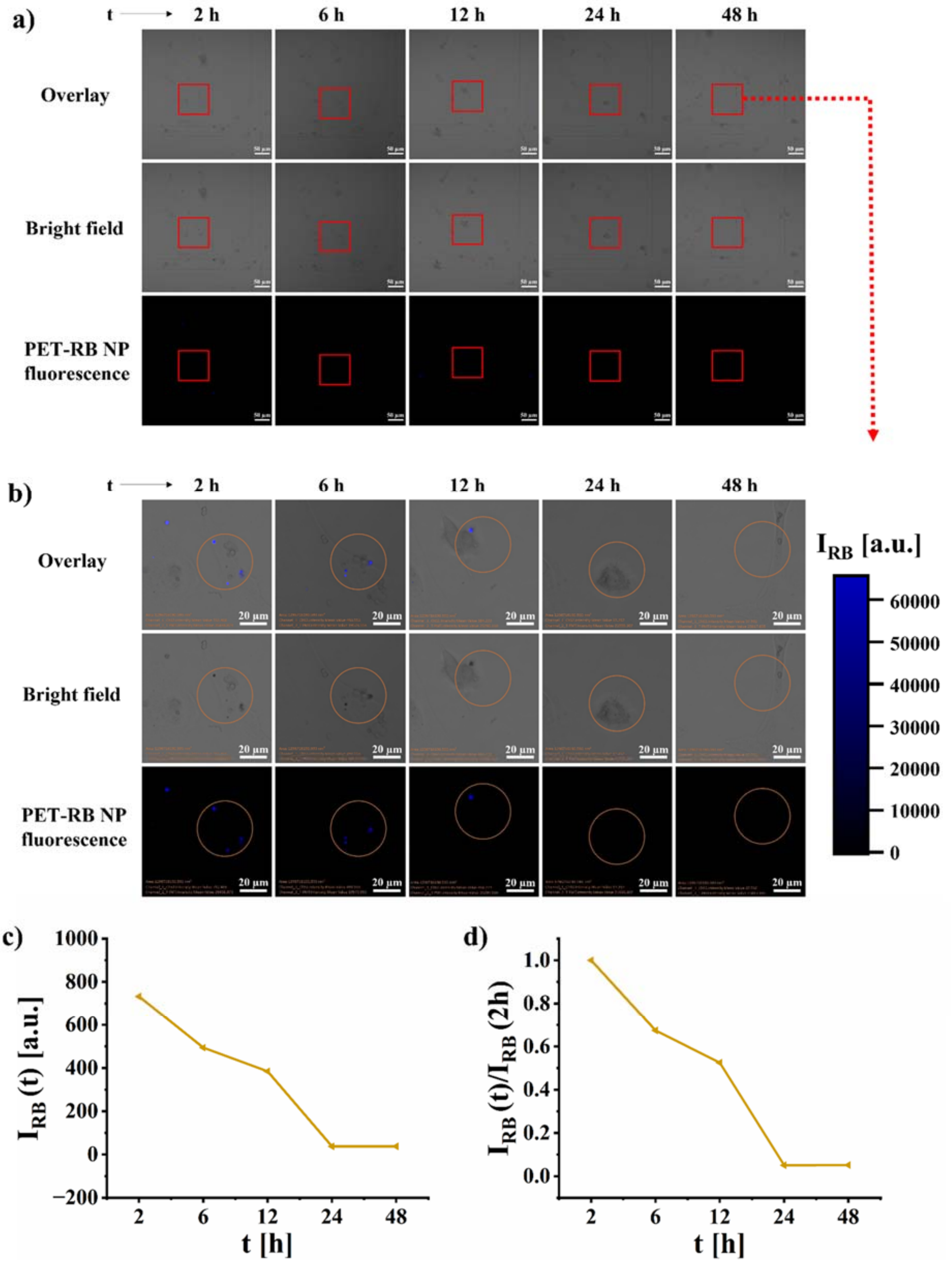

Figure S26. Example #6 of PET-RB NPs +PETase@caps. For further explanation see the legend of Figure S20.

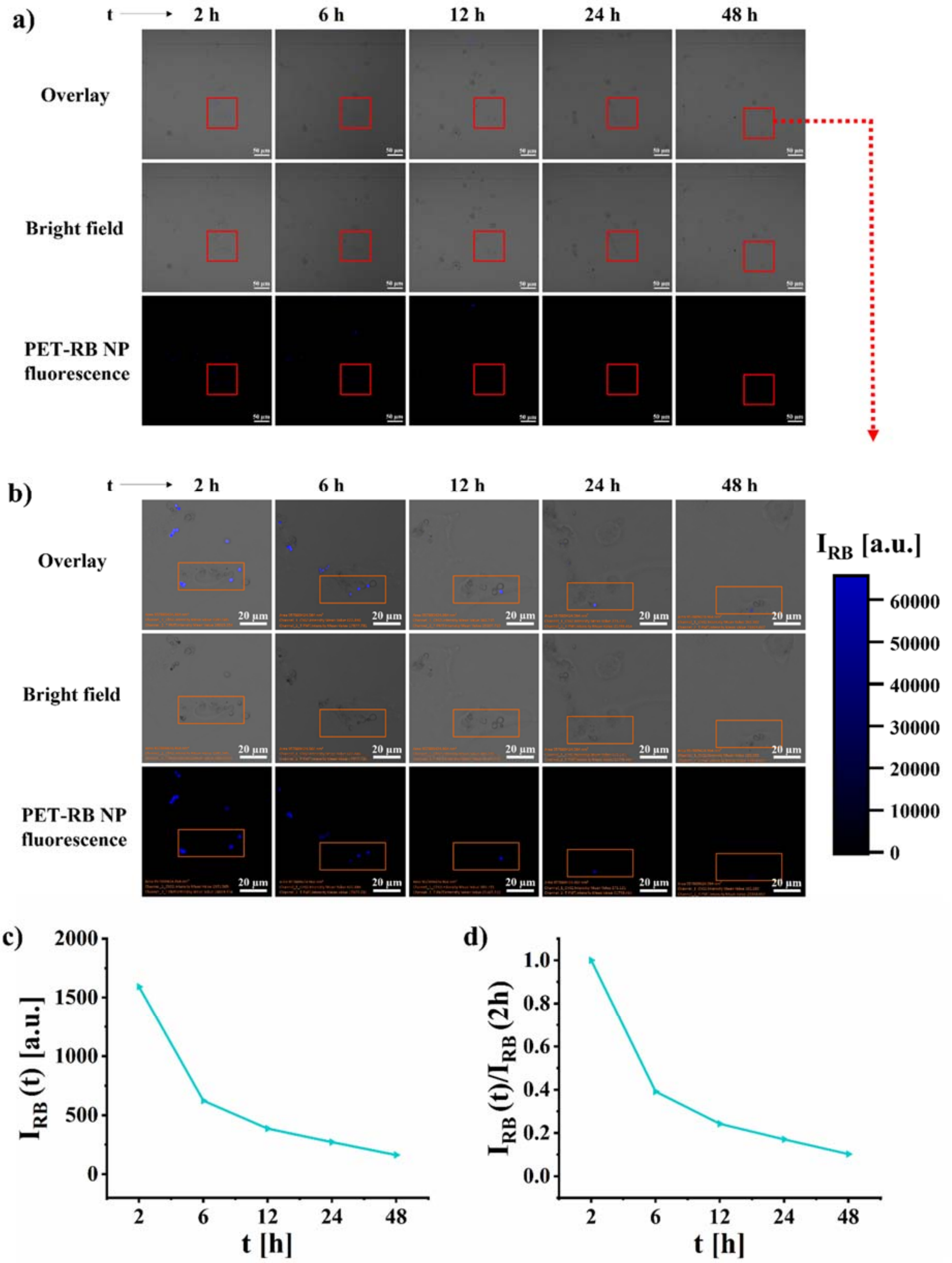

Figure S27. Example #7 of PET-RB NPs +PETase@caps. For further explanation see the legend of Figure S20.

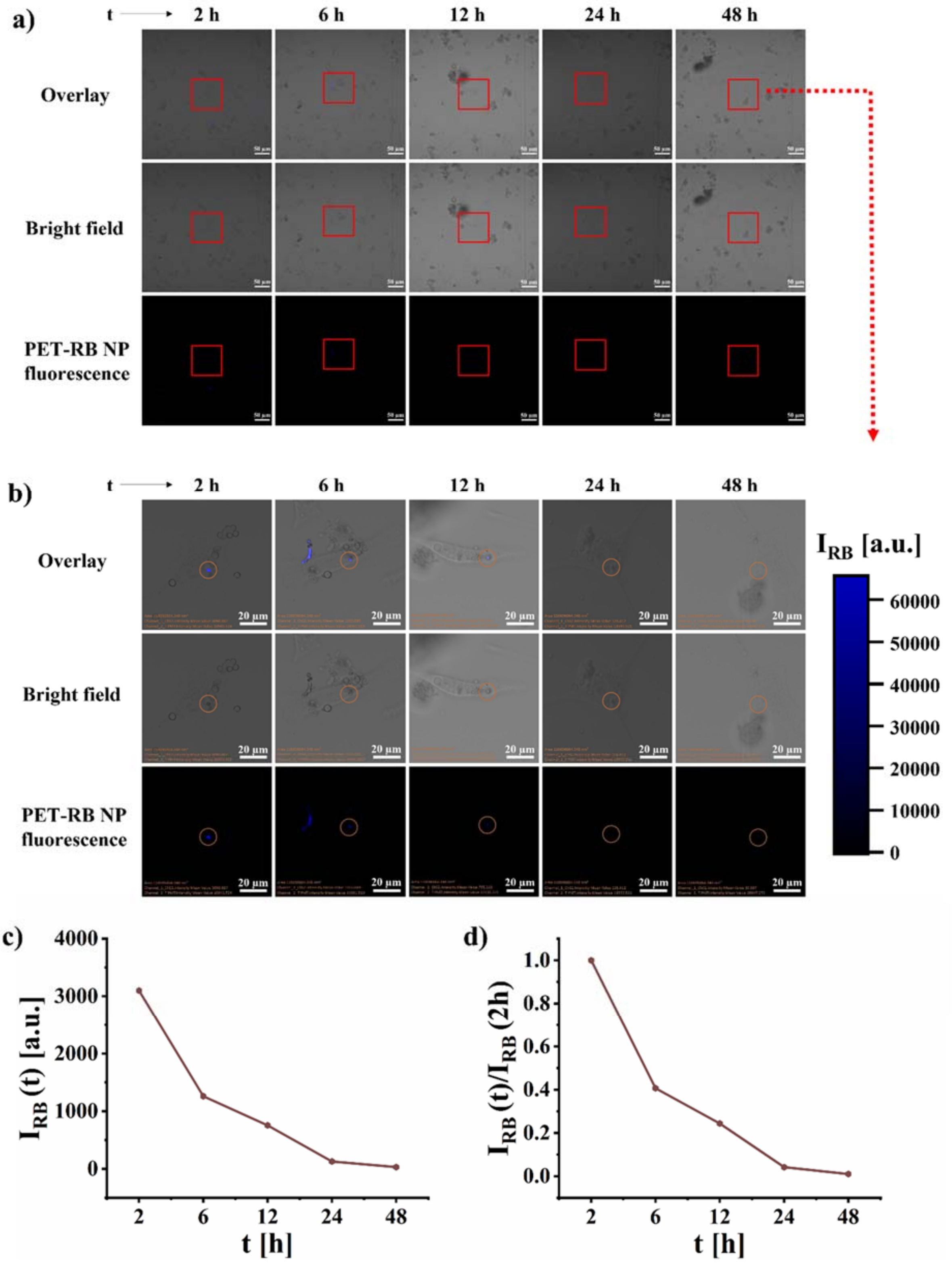

Figure S28. Example #8 of PET-RB NPs +PETase@caps. For further explanation see the legend of Figure S20.

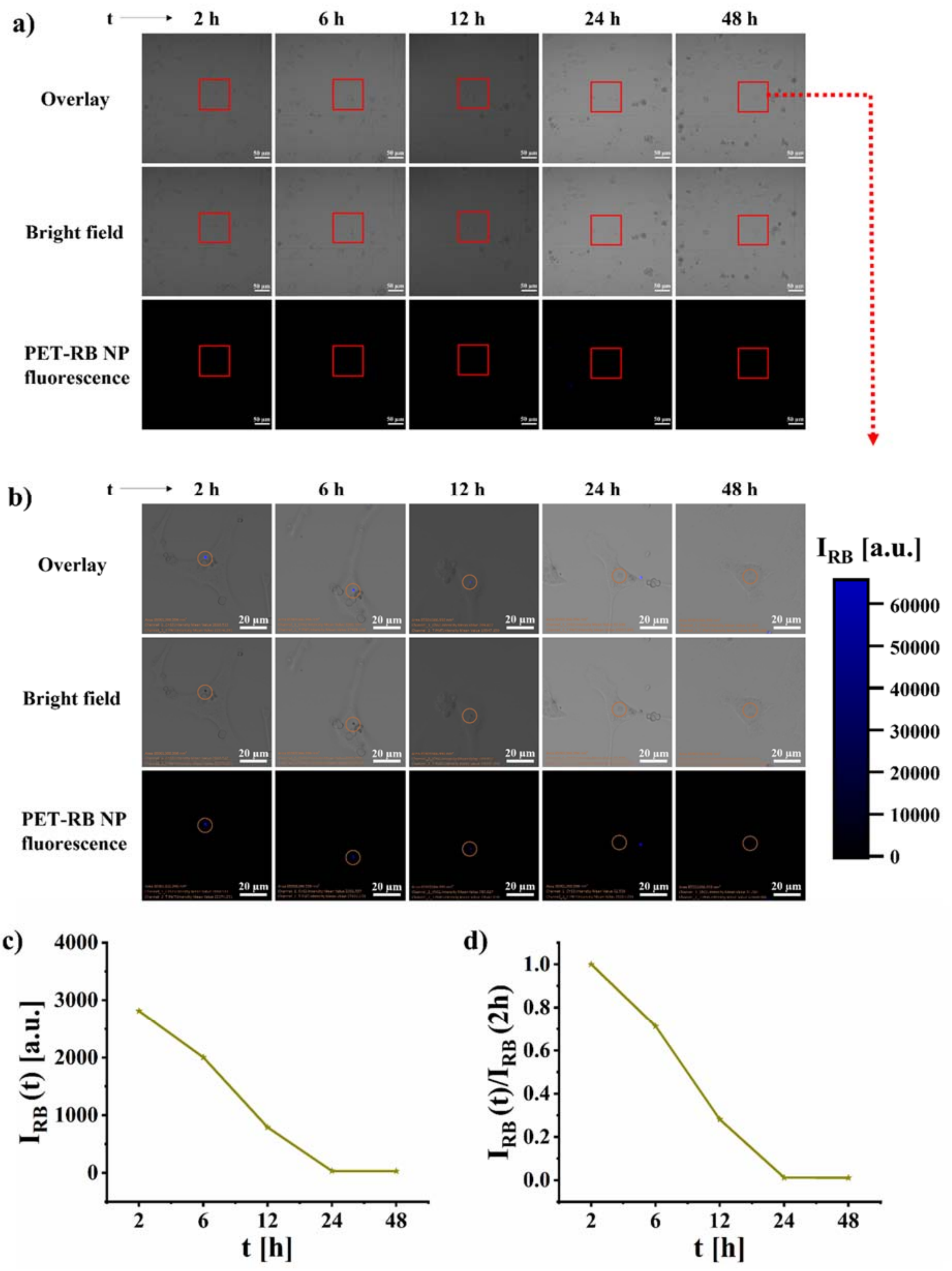

Figure S29. Example #9 of PET-RB NPs +PETase@caps. For further explanation see the legend of Figure S20.

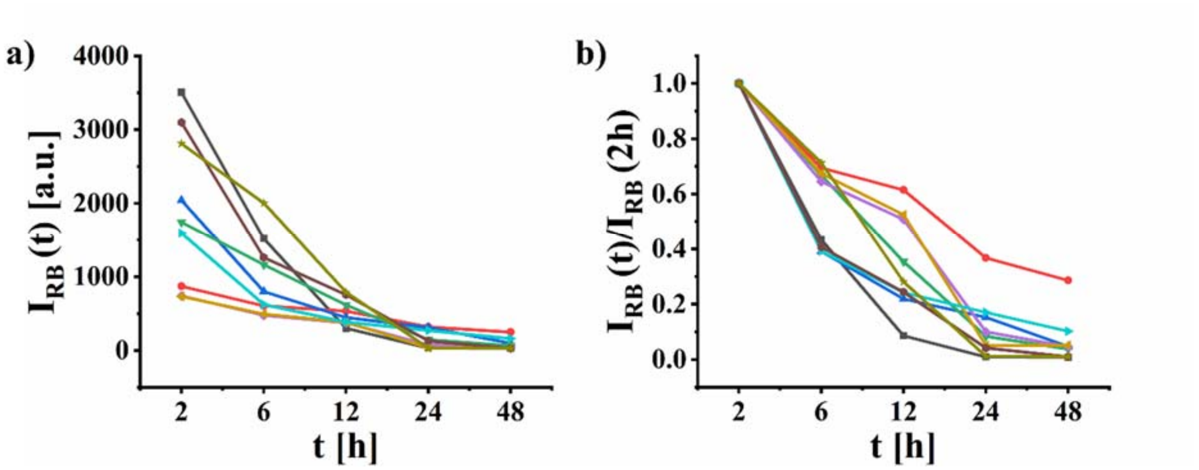

Figure S30. Compilation of all graphs shown in Figure S20c,d, and Figure S22c,d - Figure S29c,d. The colors of the curves correspond to the individual curves shown in the respective Figures. Figure b is the same as shown in Figure 5b and is shown also here, to visualize how his Figure has been composed from the data shown in Figures S20, S22 - S29.

7.3) Results of cellular exposure to PET-RB NPs only

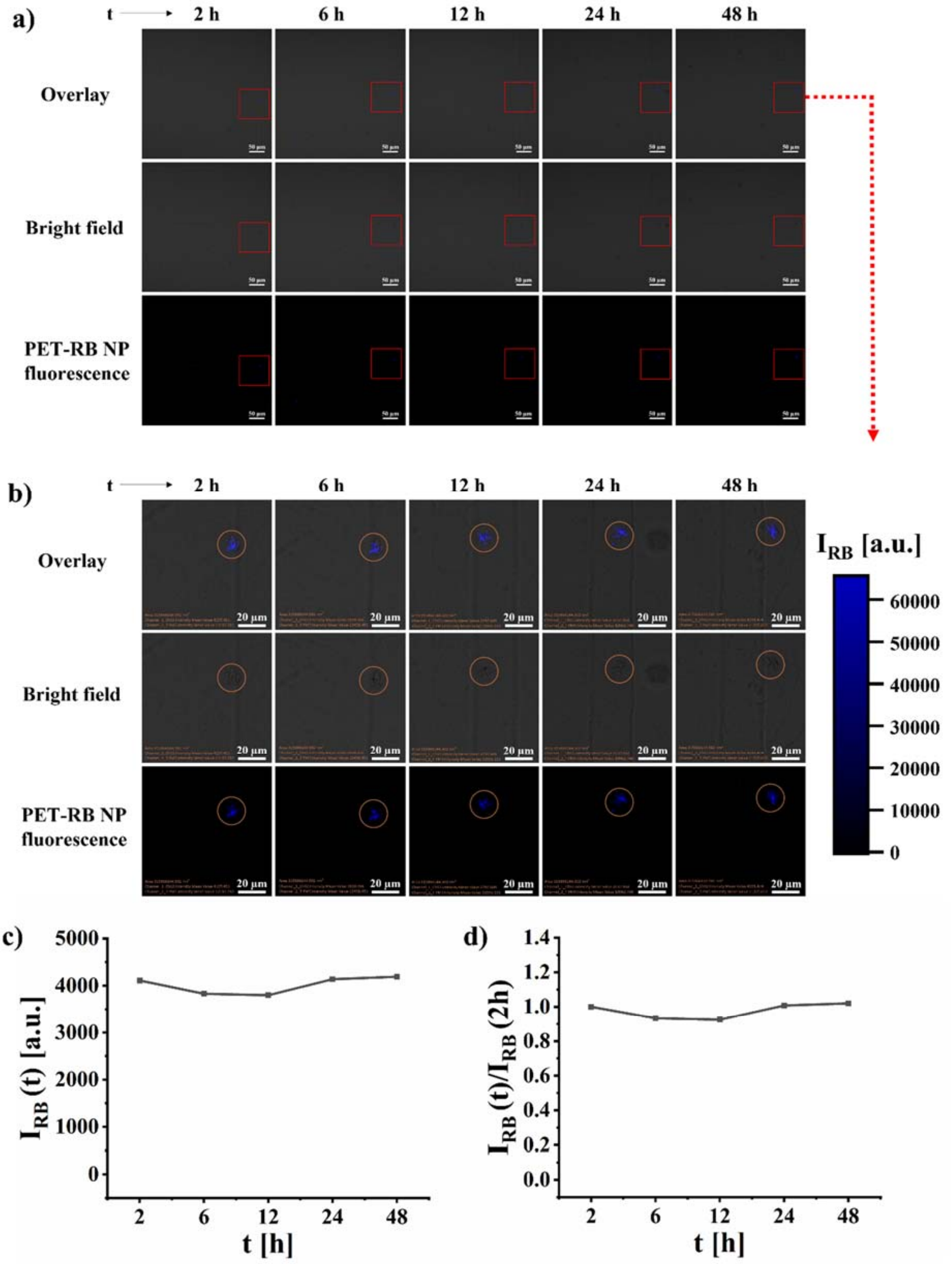

Figure S31. Example #1 of PET-RB NPs only. For further explanation see the legend of Figure S20.

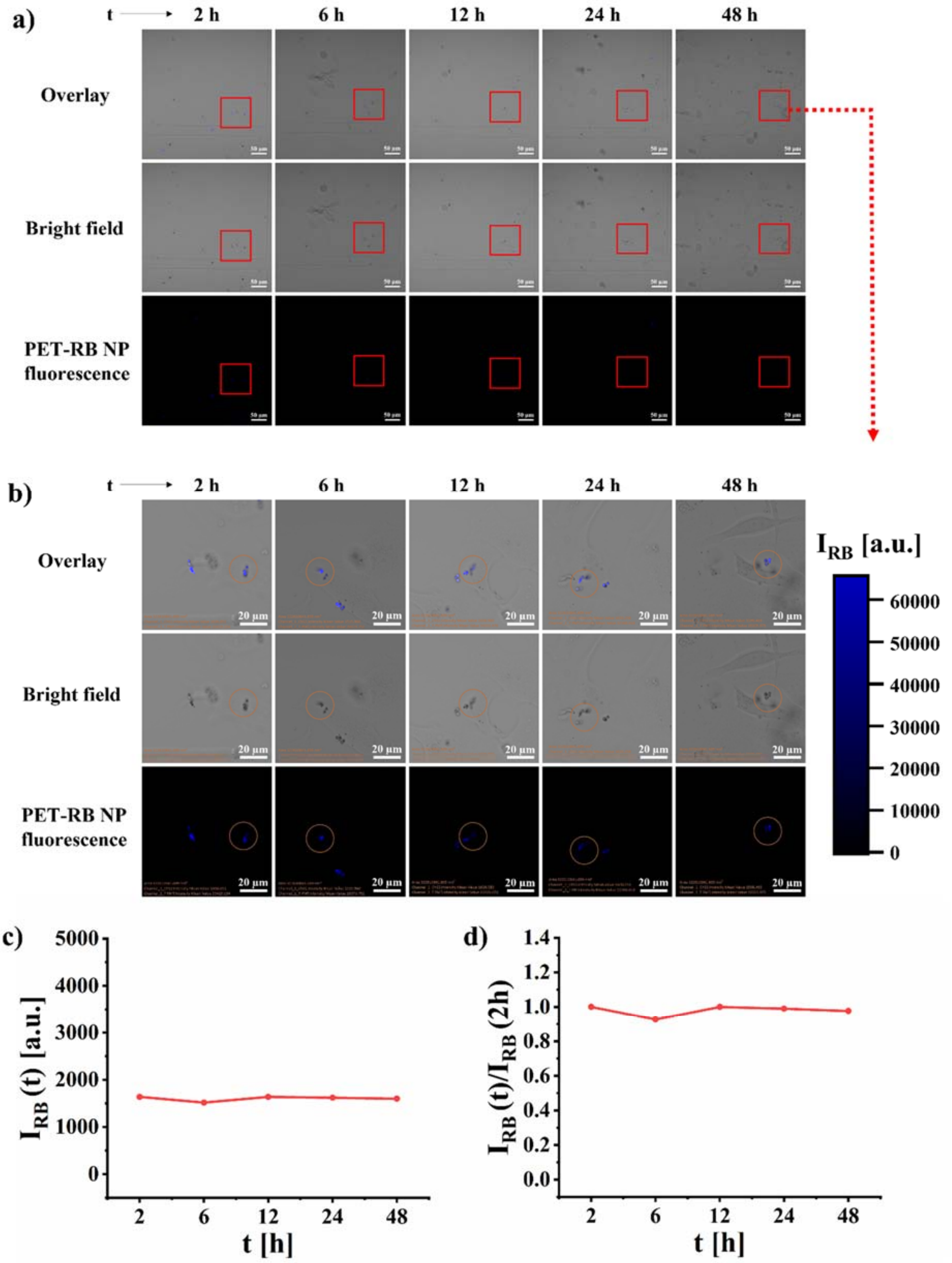

Figure S32. Example #2 of PET-RB NPs only. For further explanation see the legend of Figure S20.

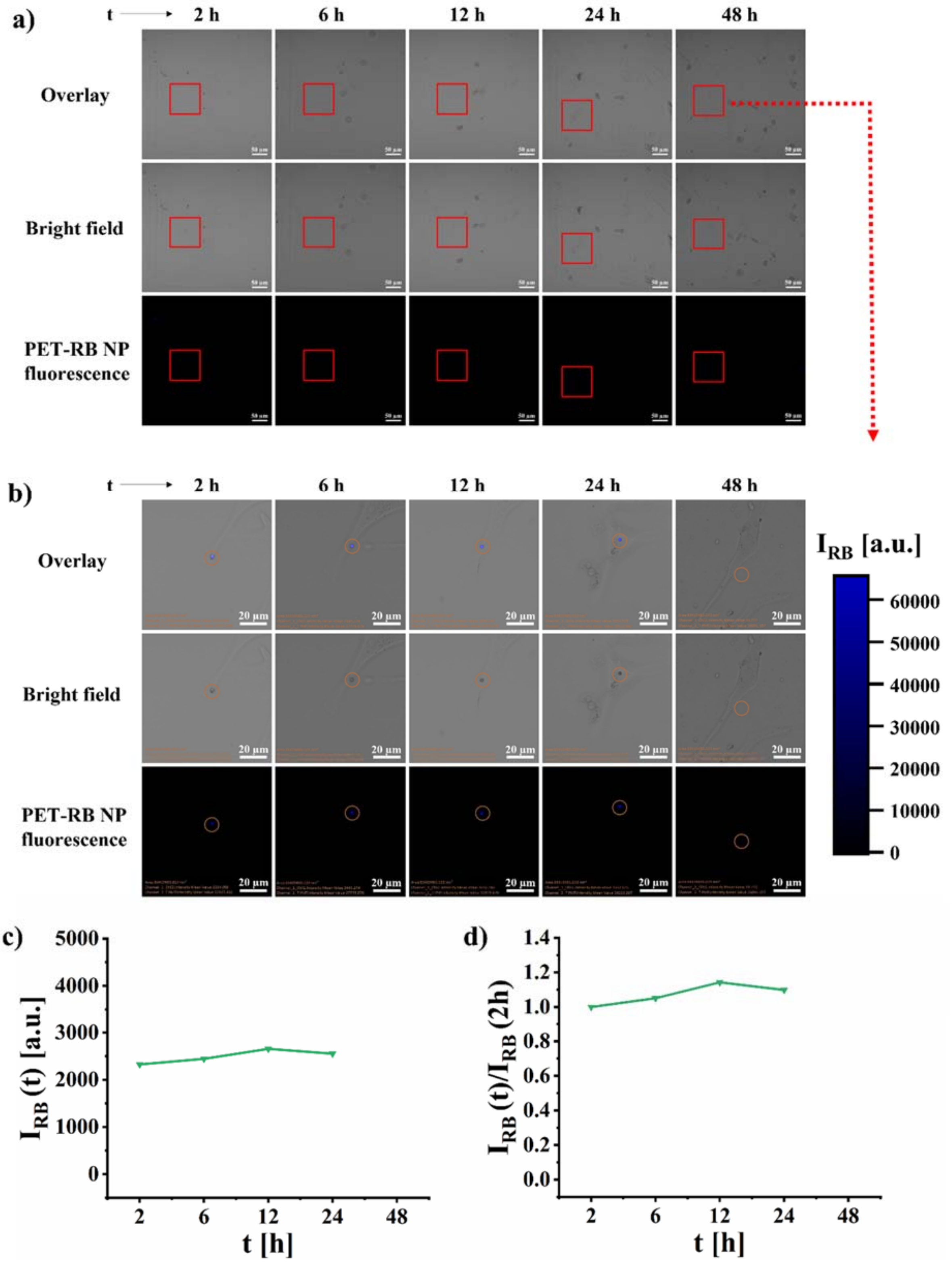

Figure S33. Example #3 of PET-RB NPs only. For further explanation see the legend of Figure S20.

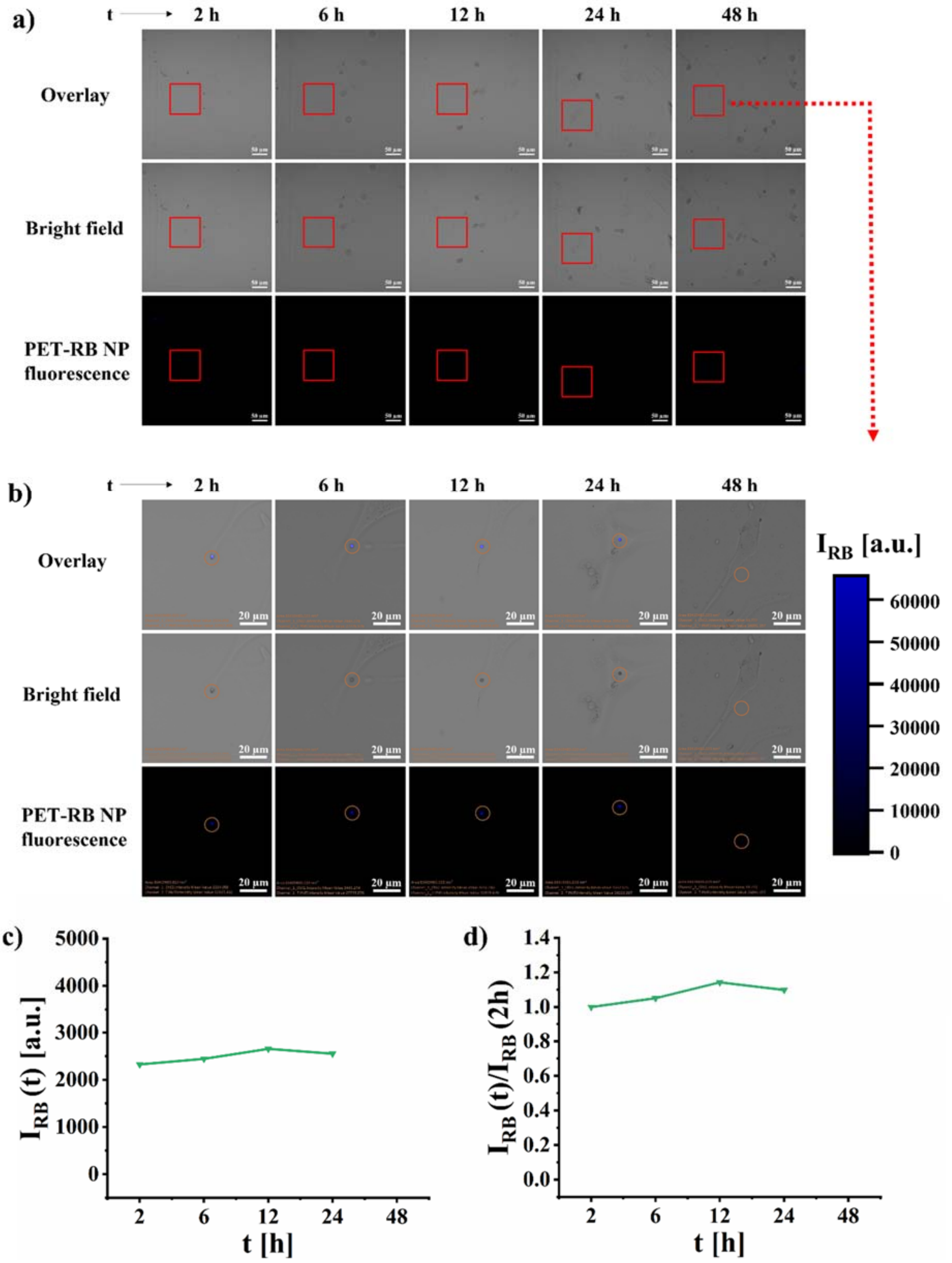

Figure S34. Example #4 of PET-RB NPs only. For further explanation see the legend of Figure S20.

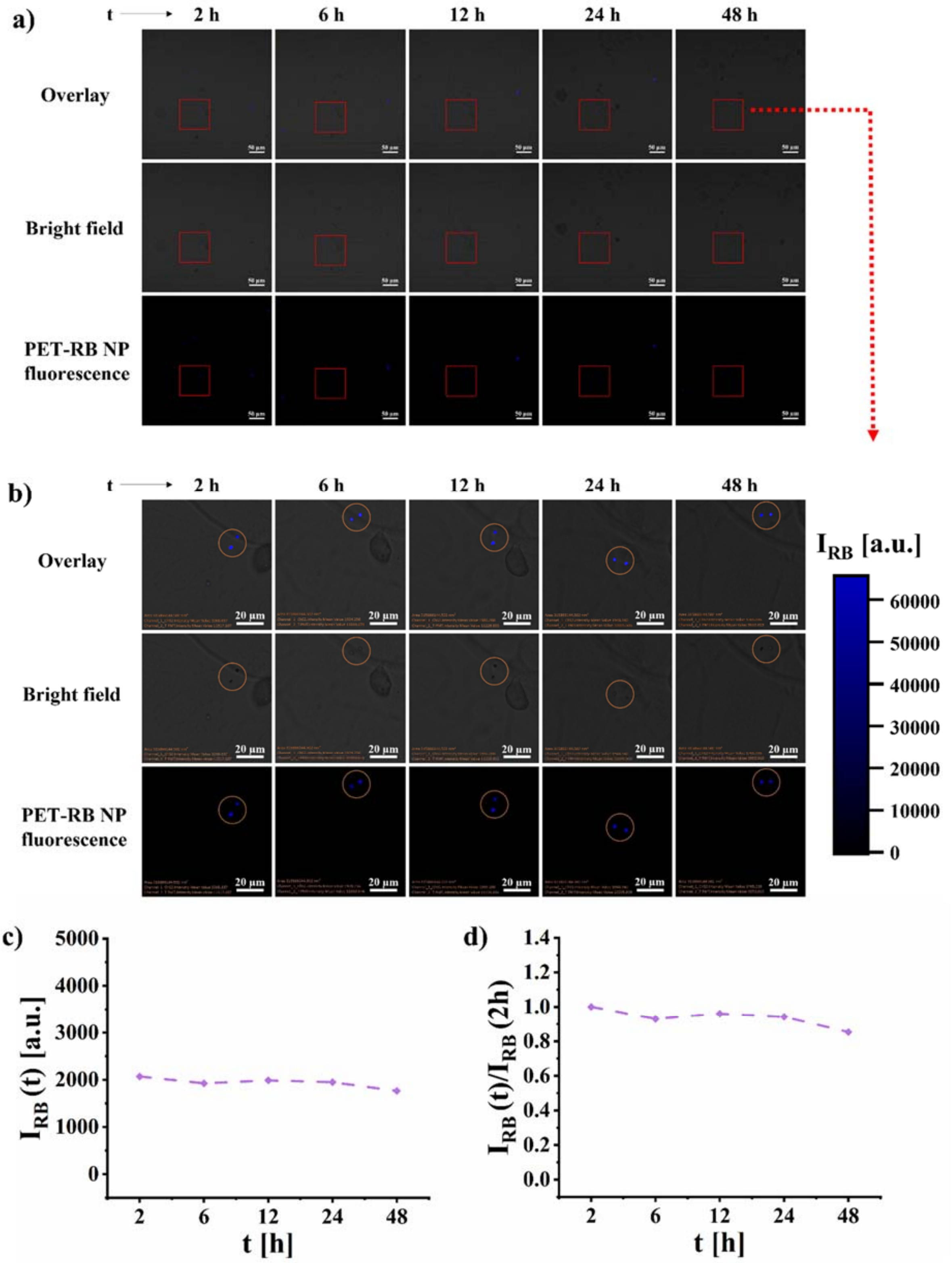

Figure S35. Example #5 of PET-RB NPs only. For further explanation see the legend of Figure S20.

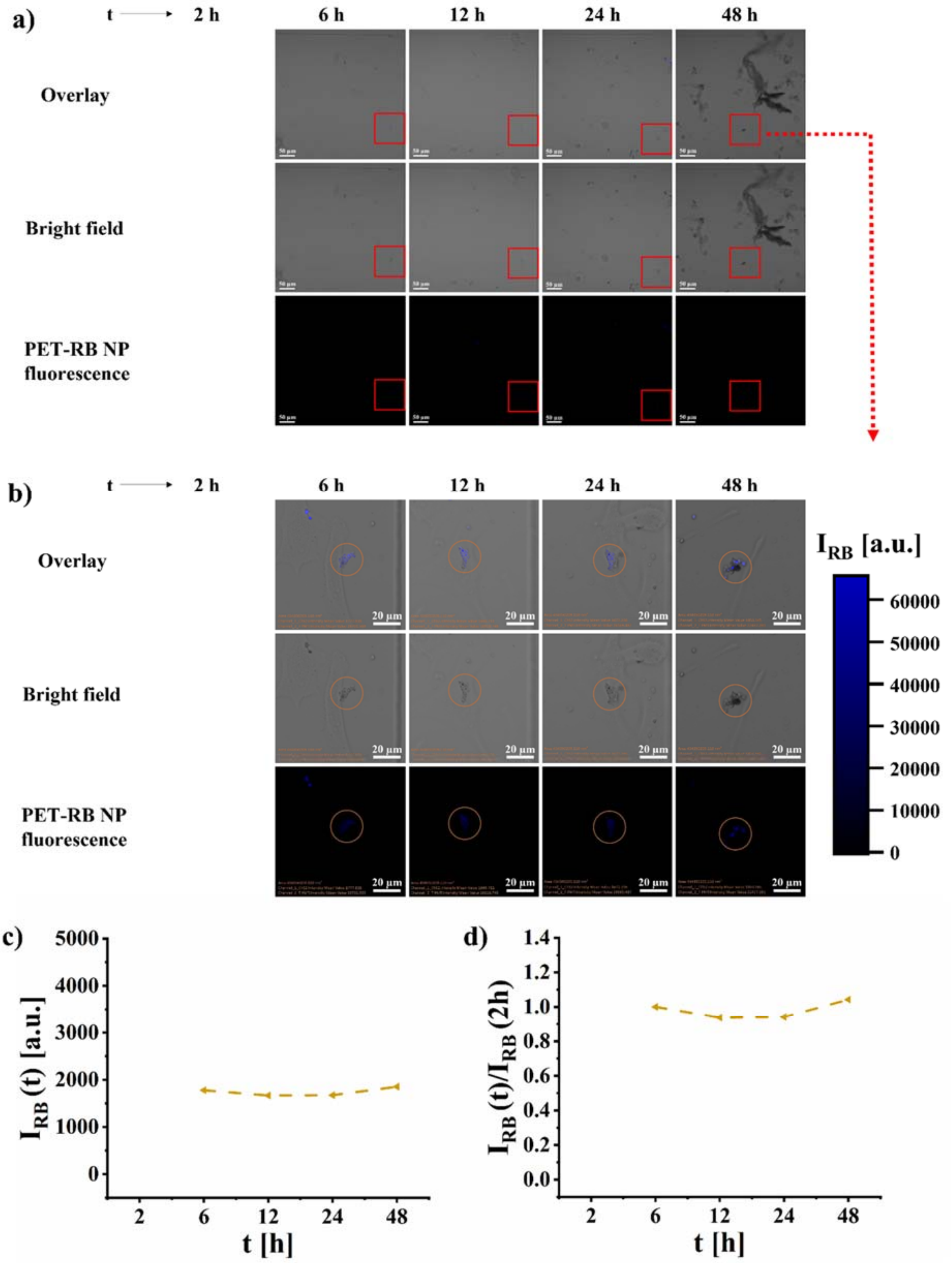

Figure S36. Example #6 of PET-RB NPs only. For further explanation see the legend of Figure S20.

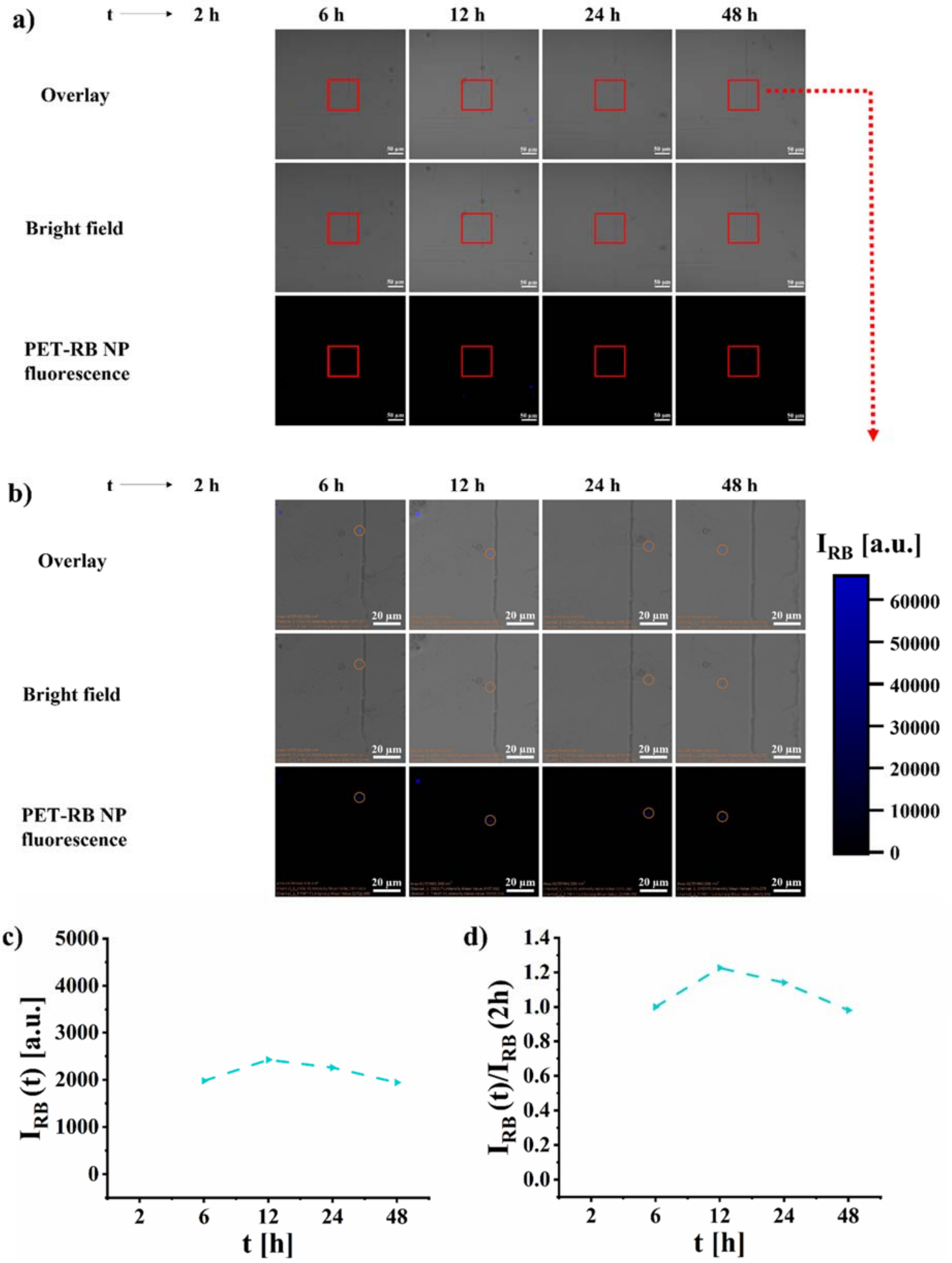

Figure S37. Example #7 of PET-RB NPs only. For further explanation see the legend of Figure S20.

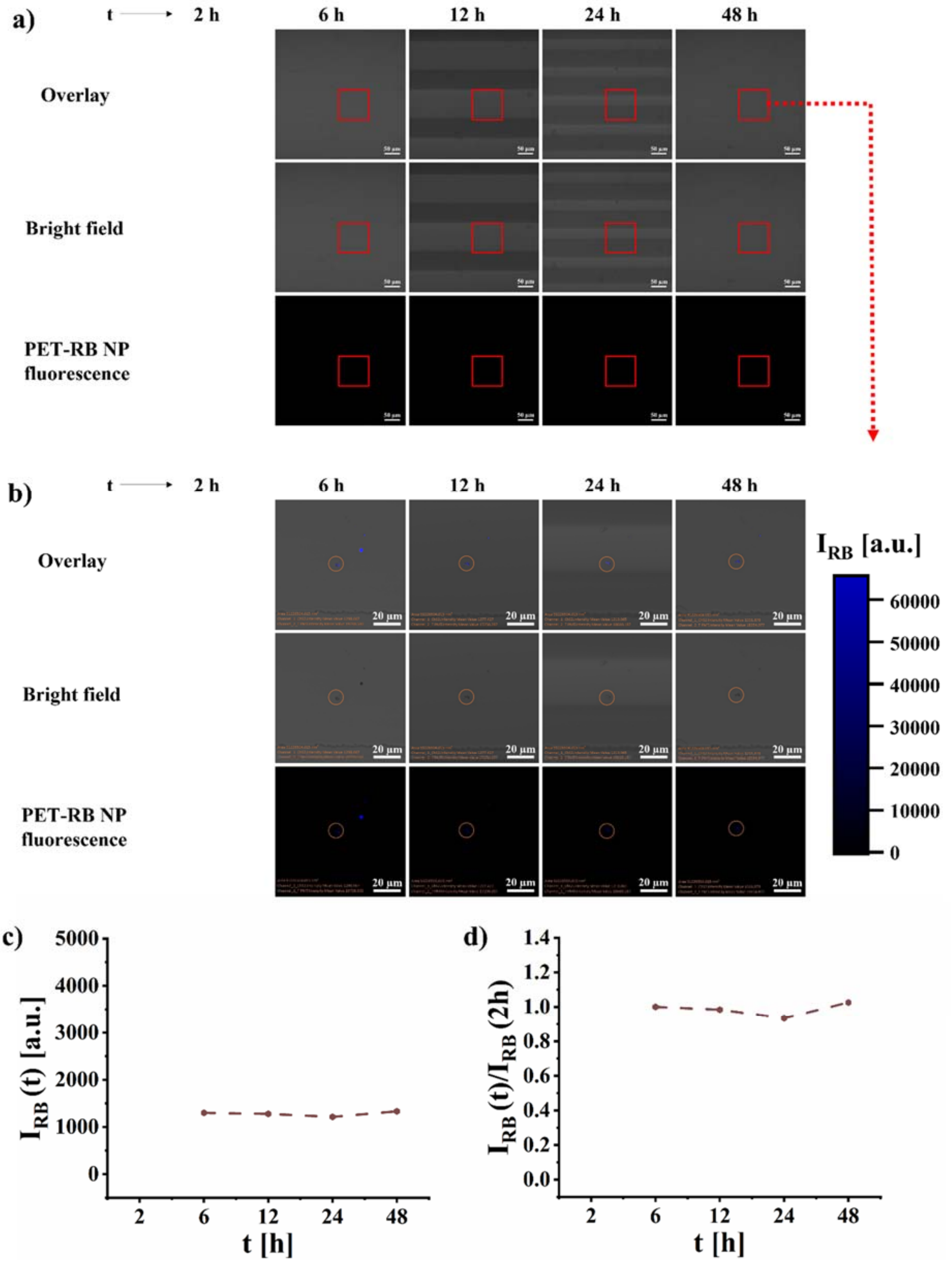

Figure S38. Example #8 of PET-RB NPs only. For further explanation see the legend of Figure S20.

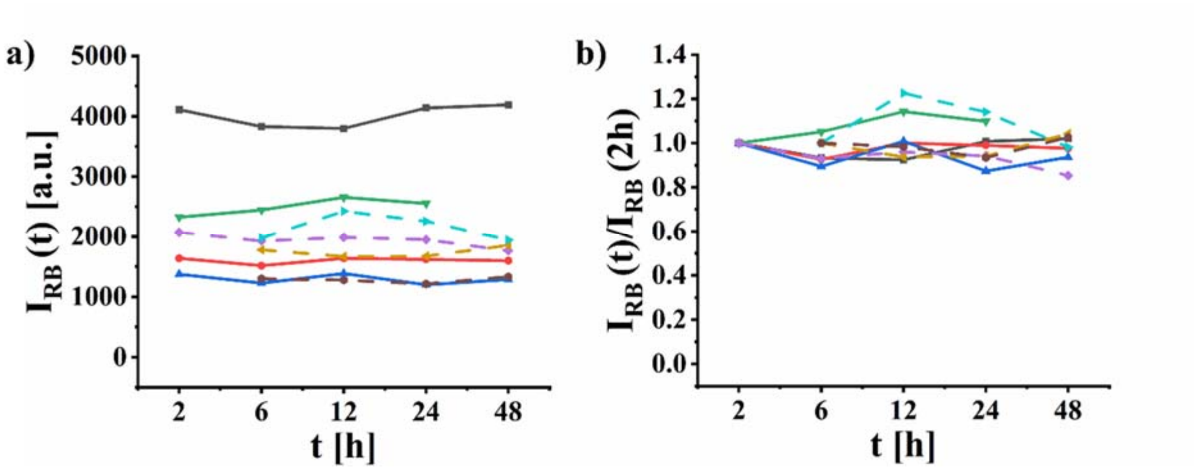

Figure S39. Compilation of all graphs shown in Figure S31c,d - Figure S38c,d. The colors of the curves correspond to the individual curves shown in the respective Figures. The solid and dashed lines represent PET-RB NPs inside and outside of cells, respectively. Figure b is the same as shown in Figure 5c and is shown also here, to visualize how his Figure has been composed from the data shown in Figures S31 - S38.

7.4) Results of cellular exposure to PETase@case only

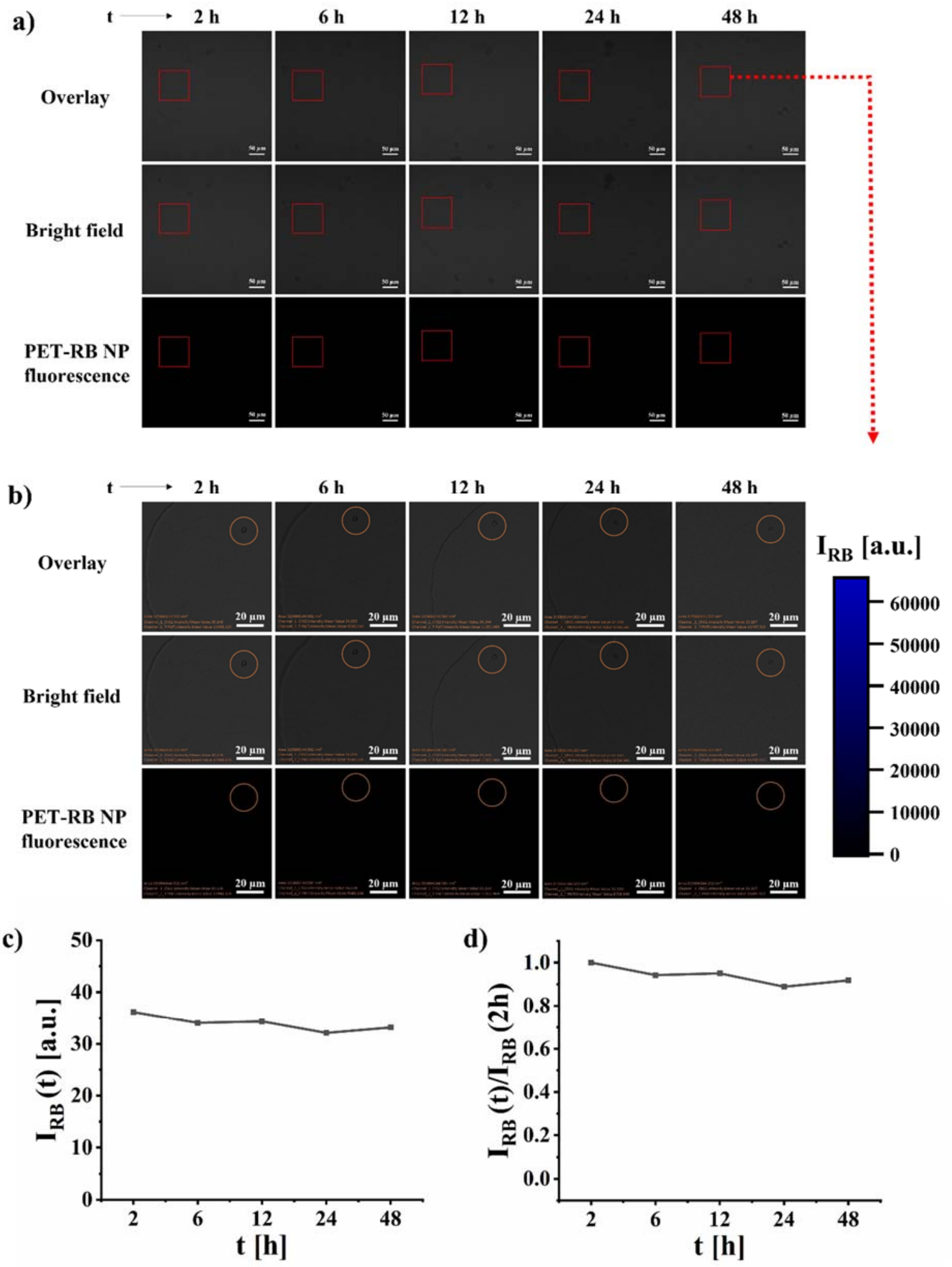

Figure S40. Example #1 of PETase@caps only. For further explanation see the legend of Figure S20.

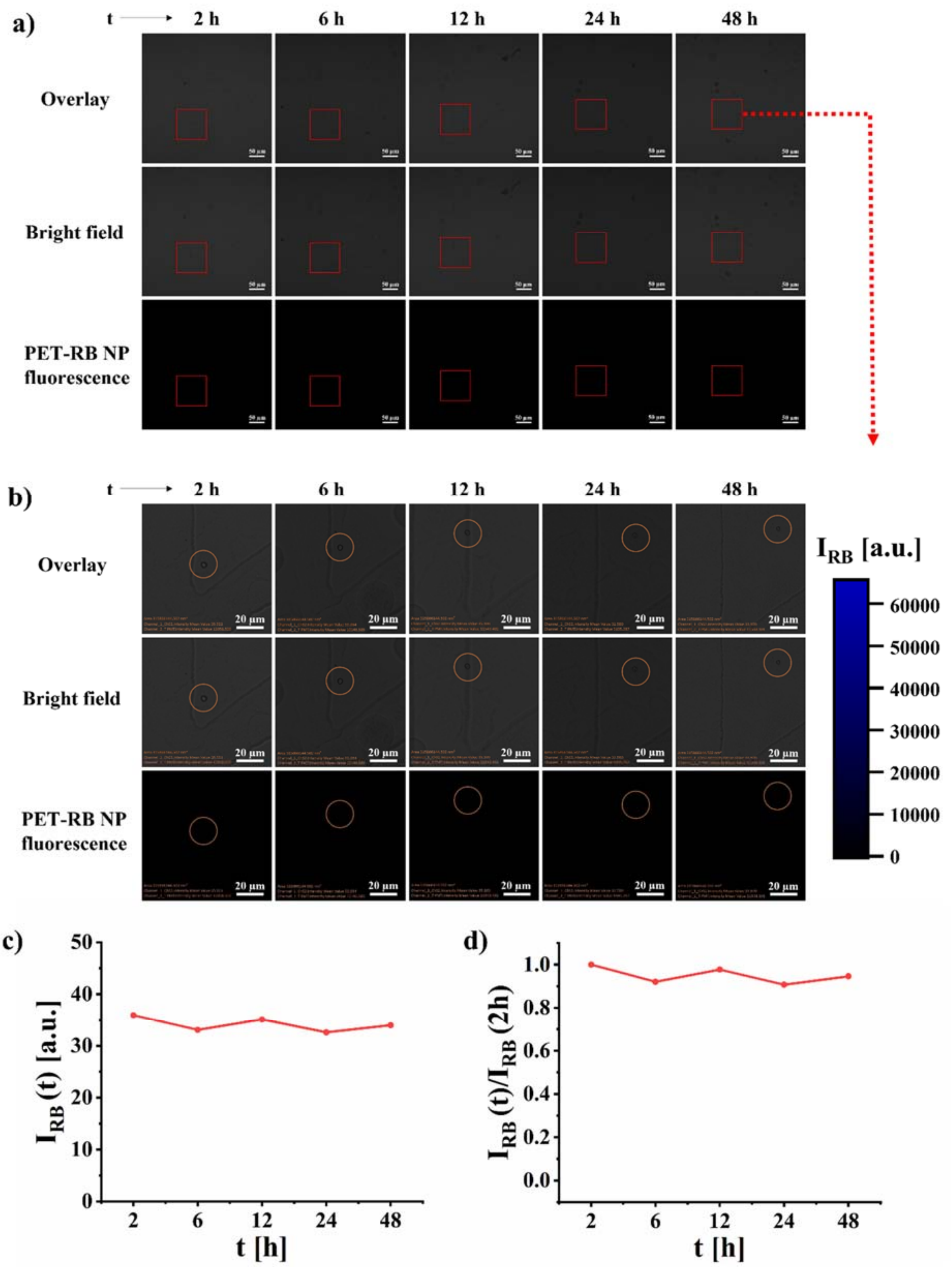

Figure S41. Example #2 of PETase@caps only. For further explanation see the legend of Figure S20.

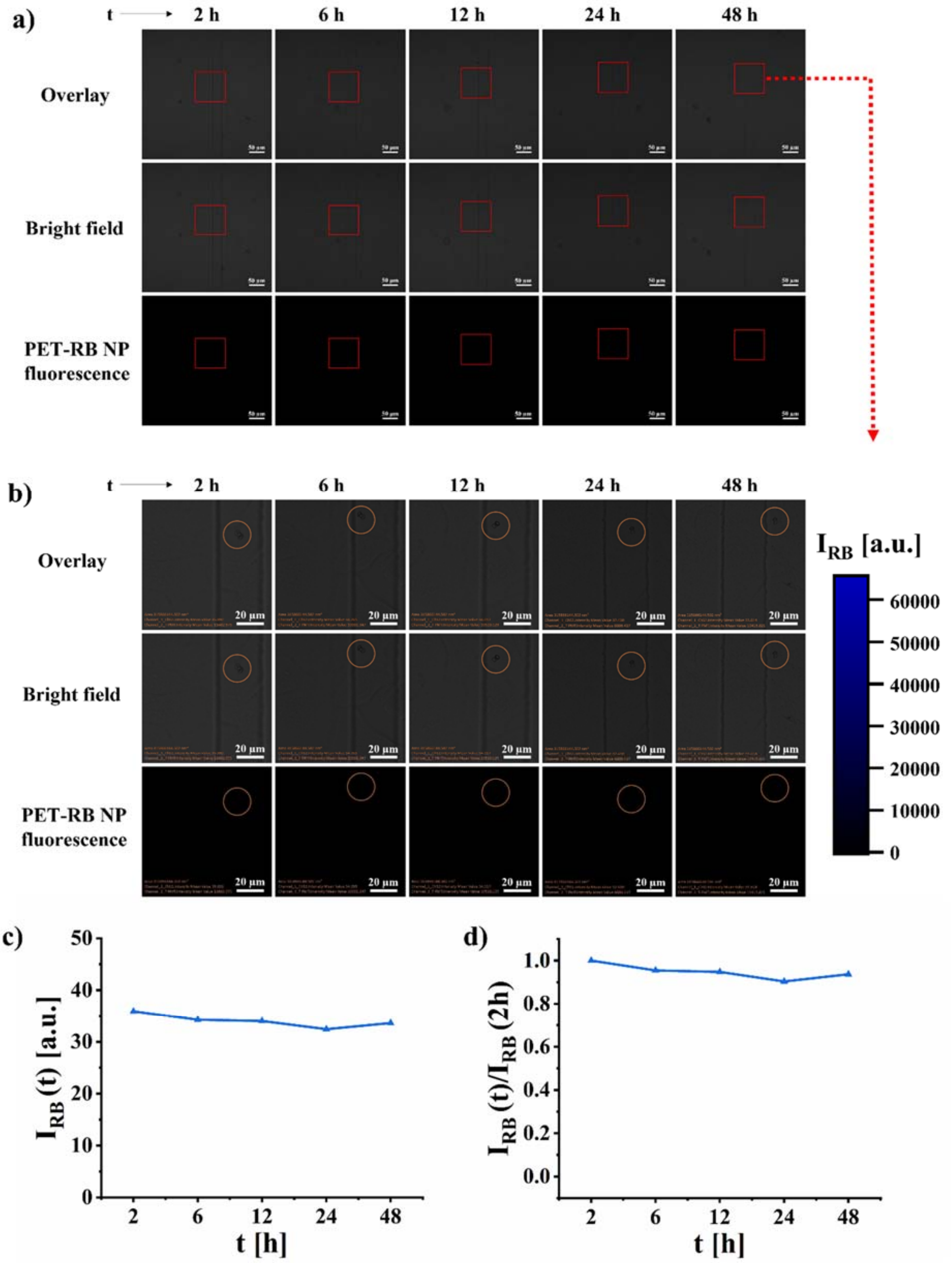

Figure S42. Example #3 of PETase@caps only. For further explanation see the legend of Figure S20.

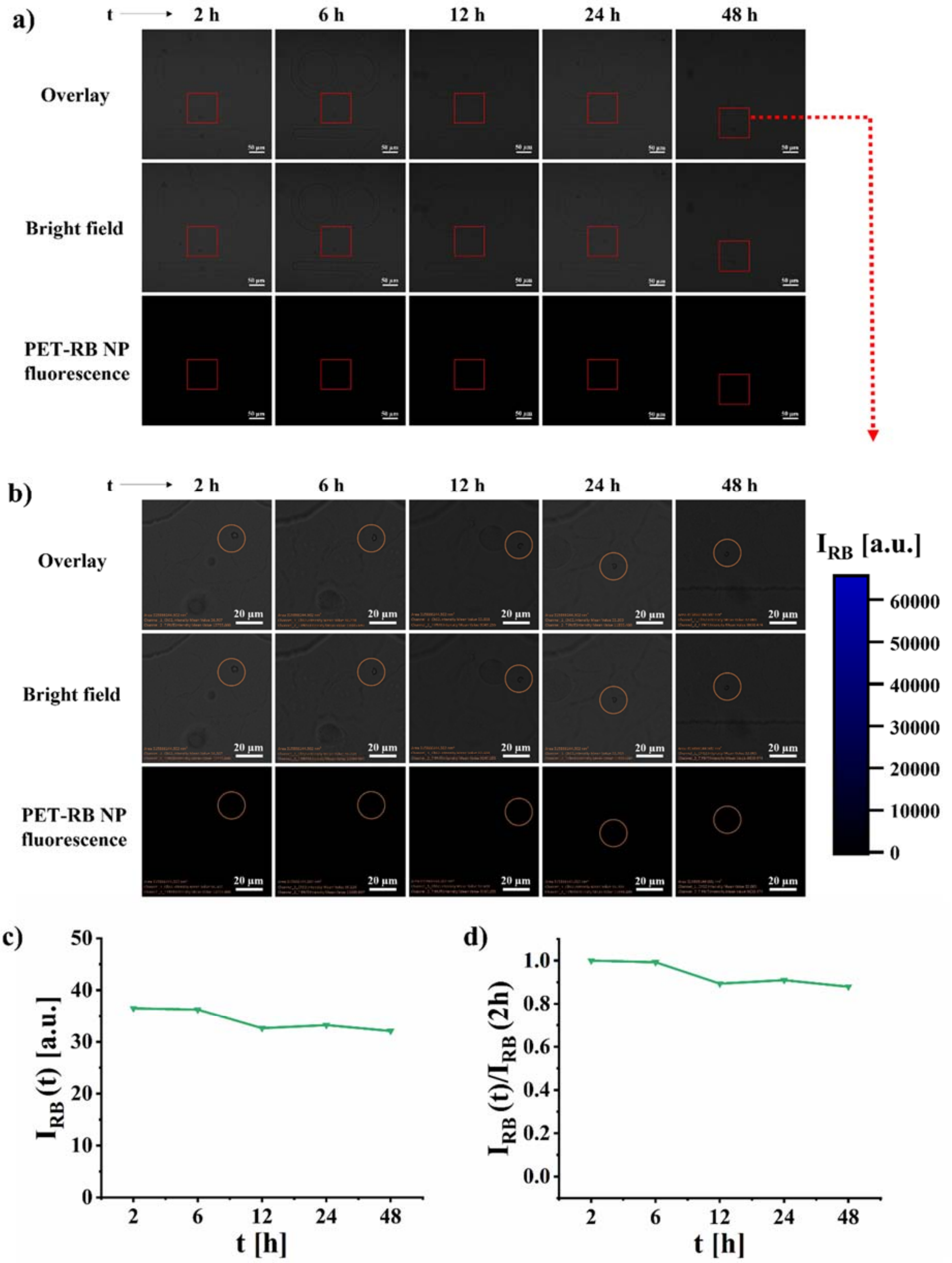

Figure S43. Example #4 of PETase@caps only. For further explanation see the legend of Figure S20.

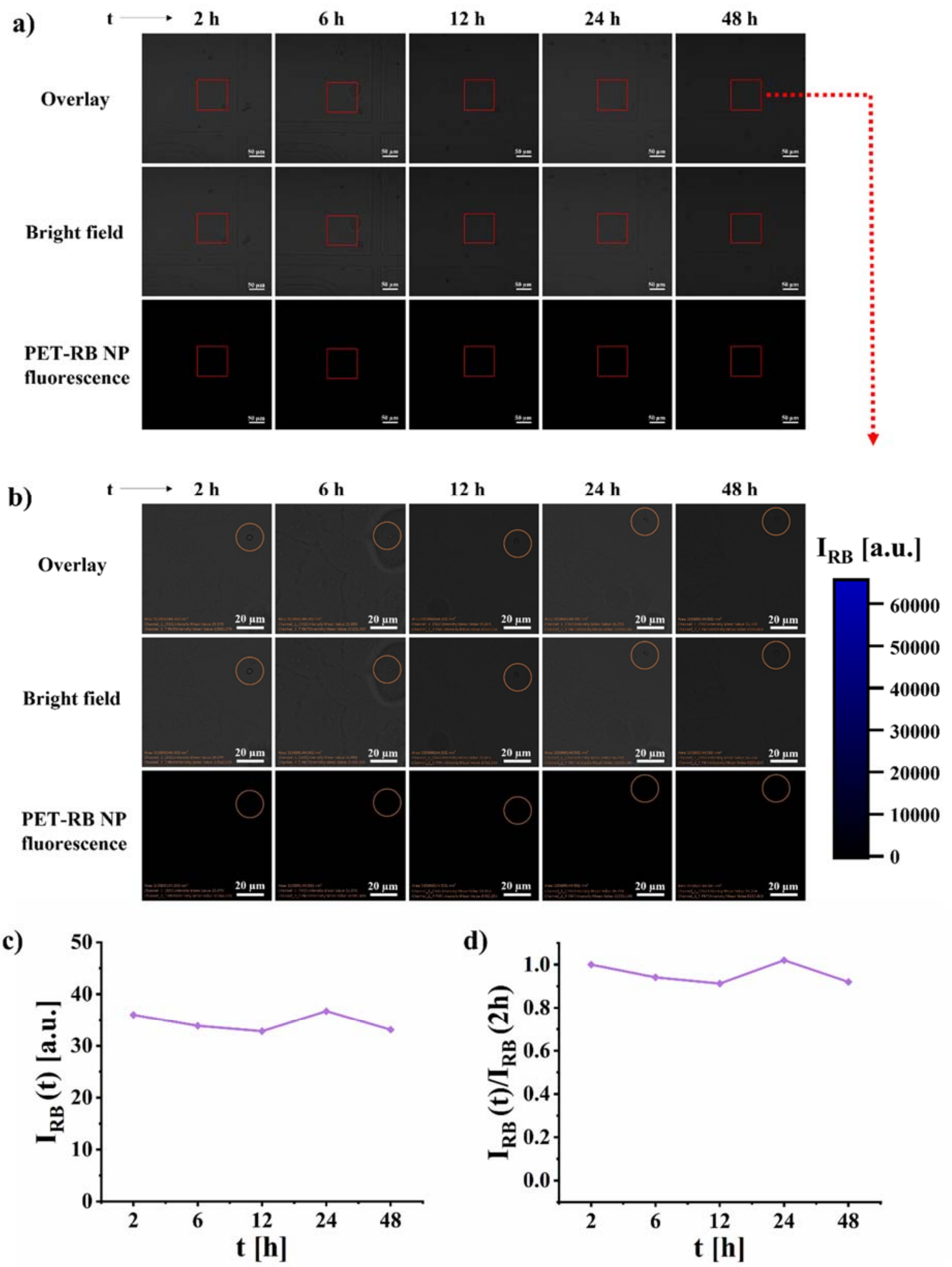

Figure S44. Example #5 of PETase@caps only. For further explanation see the legend of Figure S20.

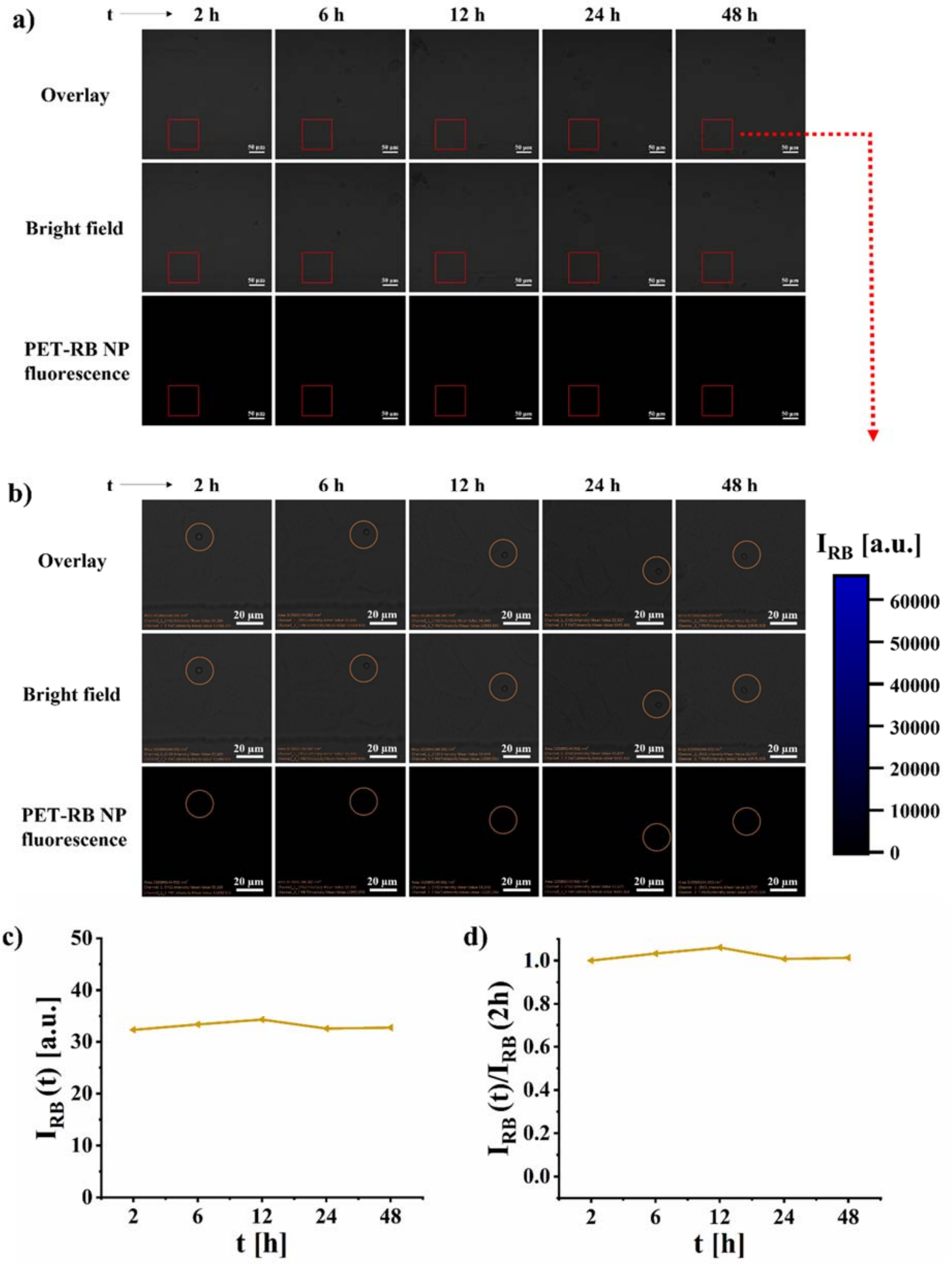

Figure S45. Example #6 of PETase@caps only. For further explanation see the legend of Figure S20.

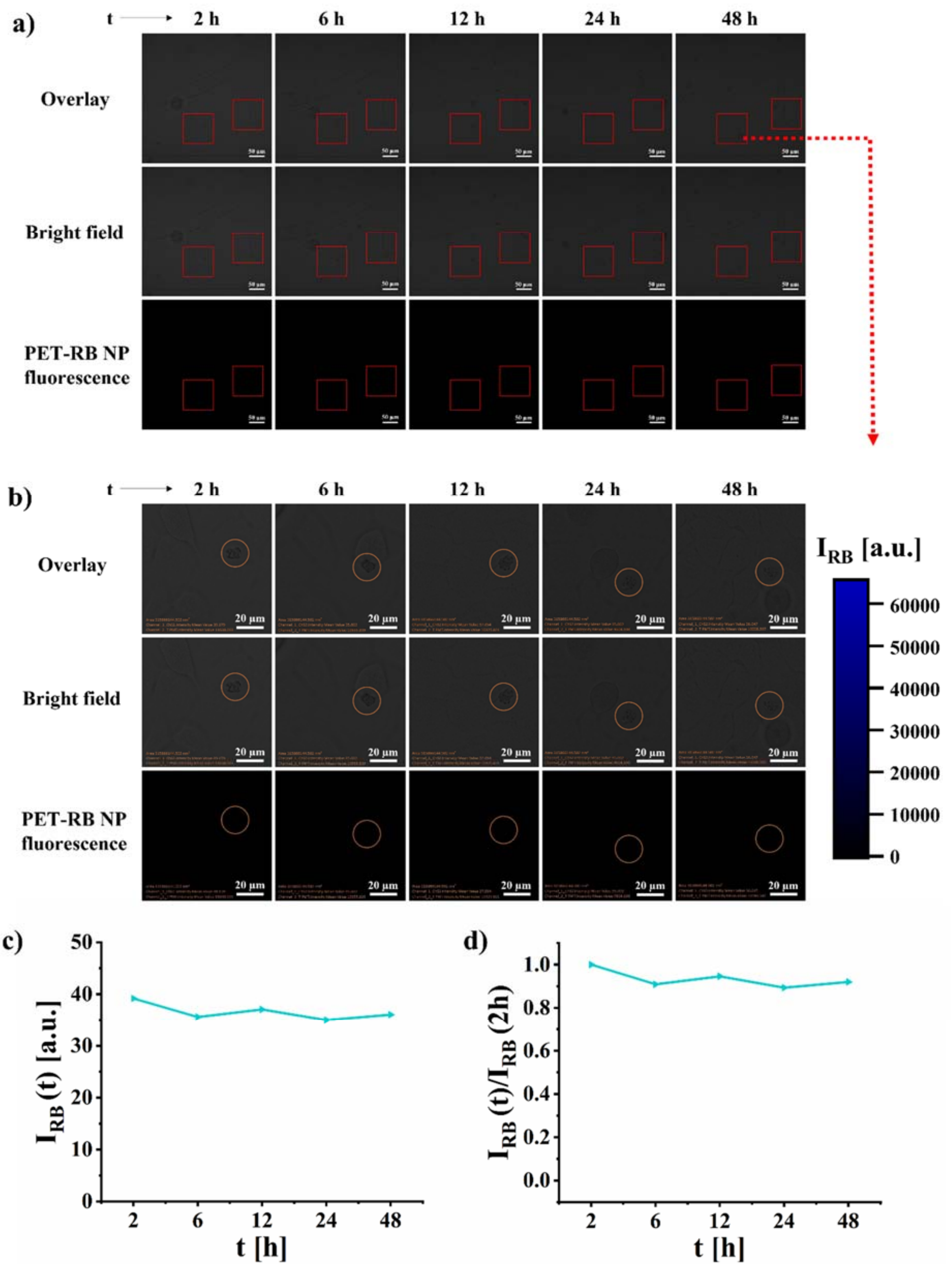

Figure S46. Example #7 of PETase@caps only. For further explanation see the legend of Figure S20.

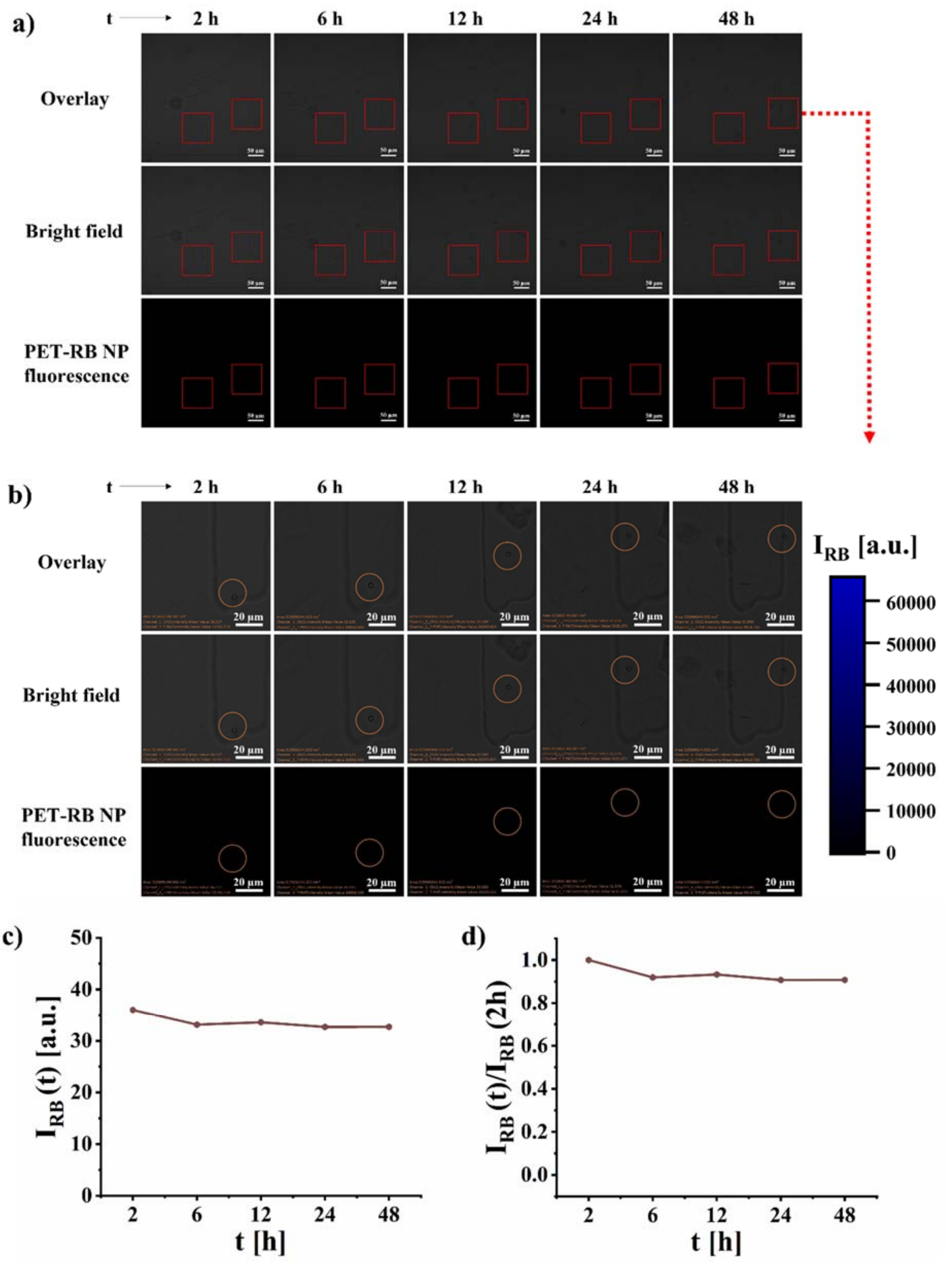

Figure S47. Example #8 of PETase@caps only. For further explanation see the legend of Figure S20.

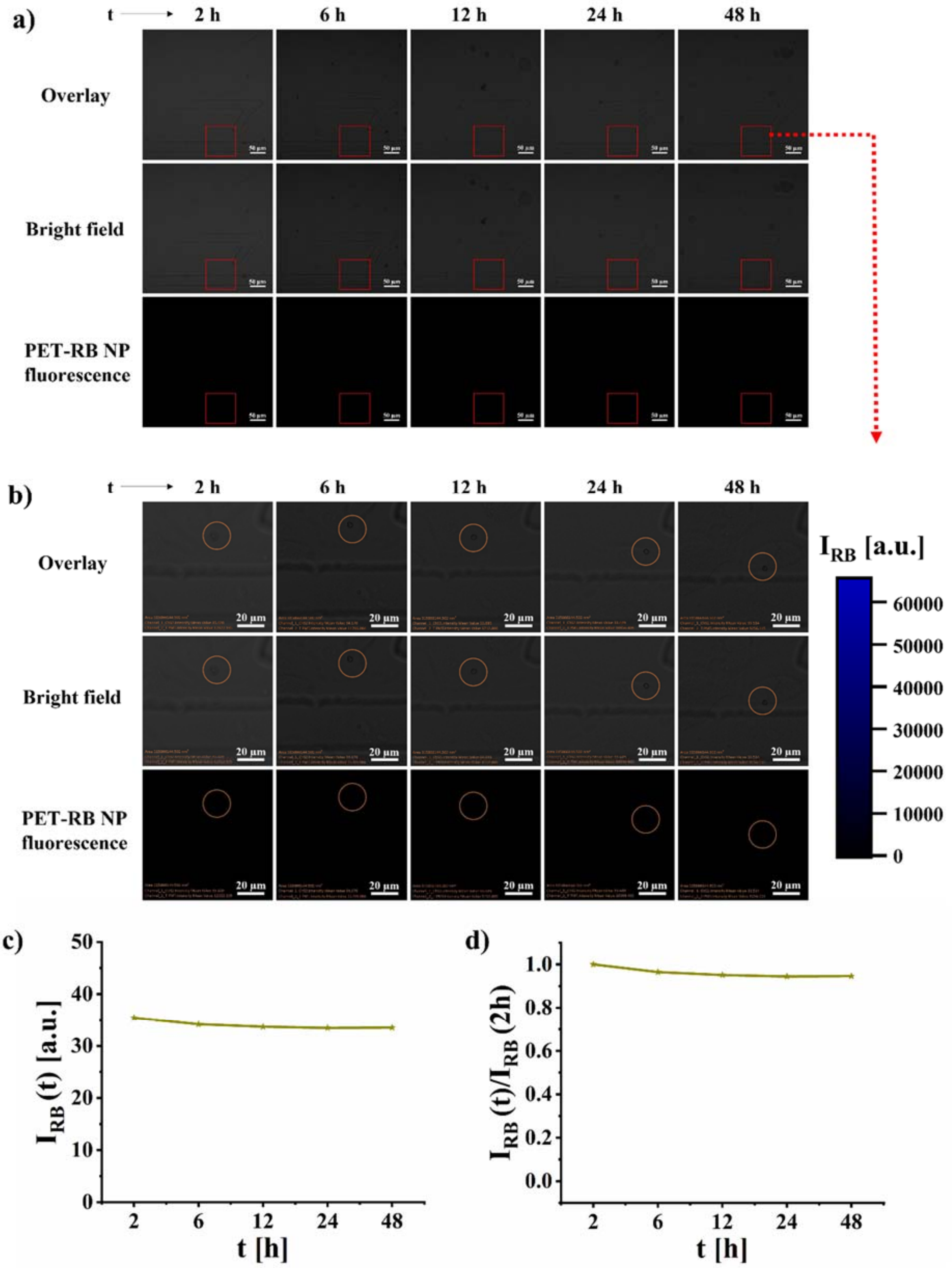

Figure S48. Example #9 of PETase@caps only. For further explanation see the legend of Figure S20.

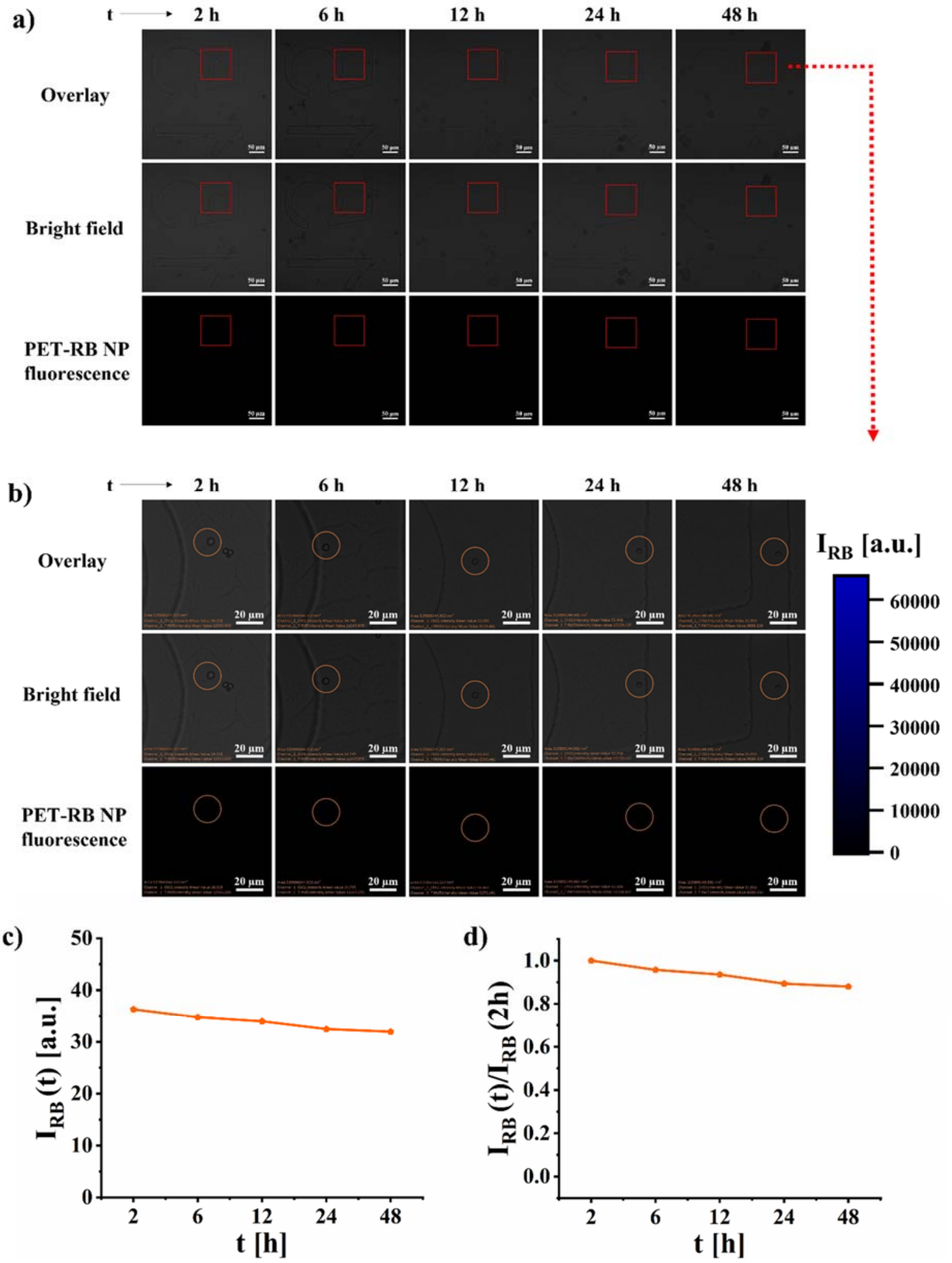

Figure S49. Example #10 of PETase@caps only. For further explanation see the legend of Figure S20.

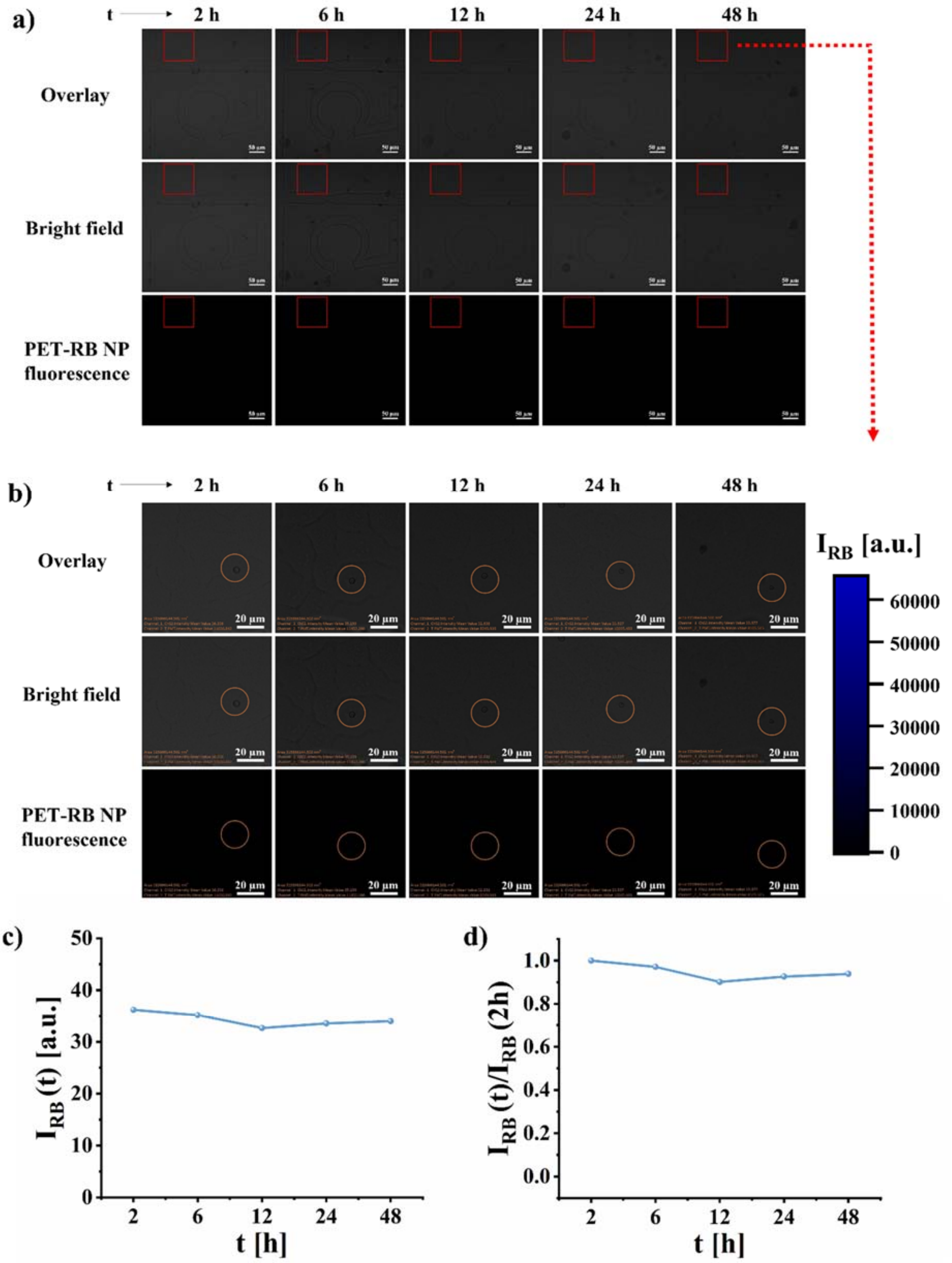

Figure S50. Example #11 of PETase@caps only. For further explanation see the legend of Figure S20.

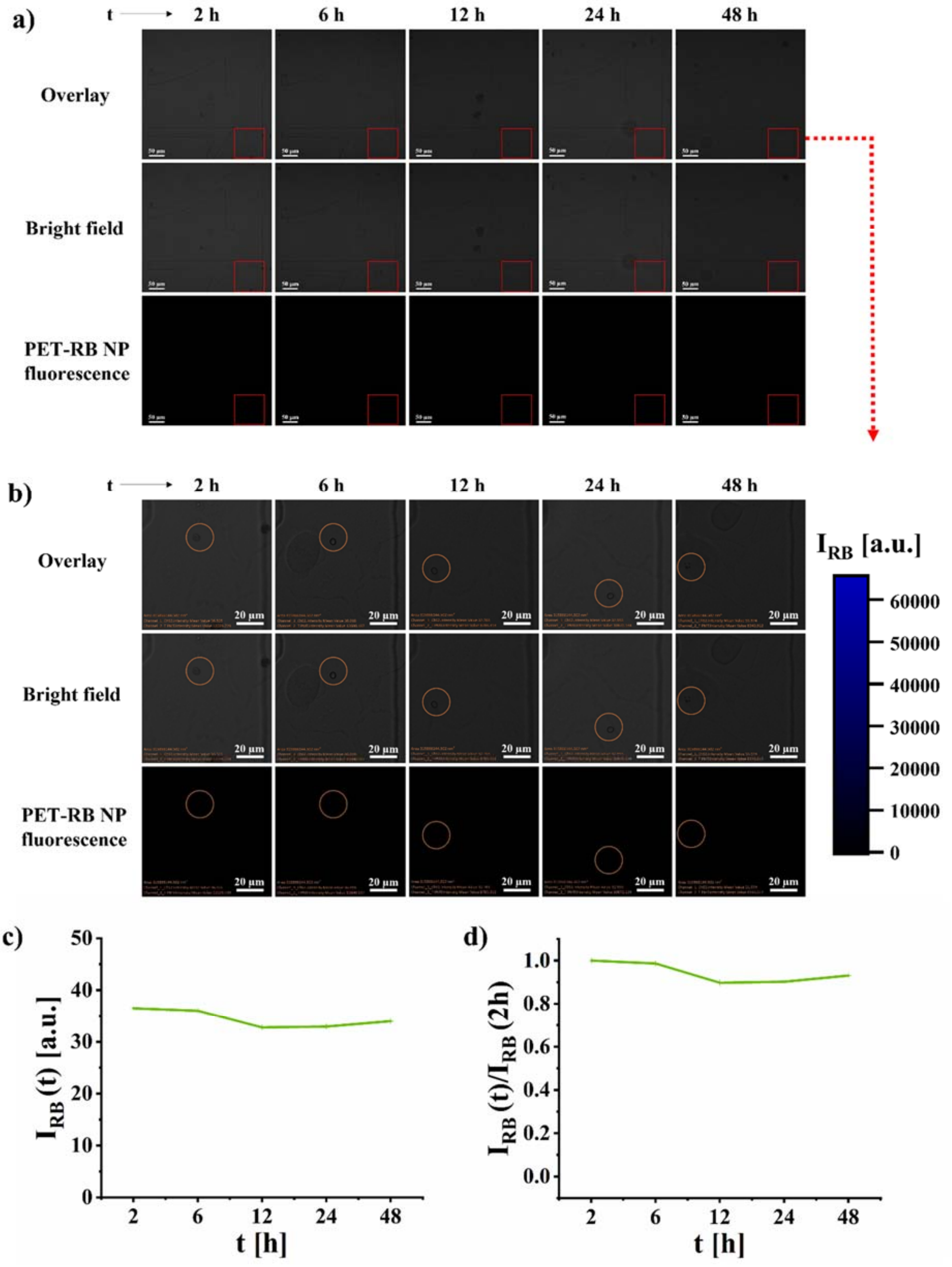

Figure S51. Example #12 of PETase@caps only. For further explanation see the legend of Figure S20.

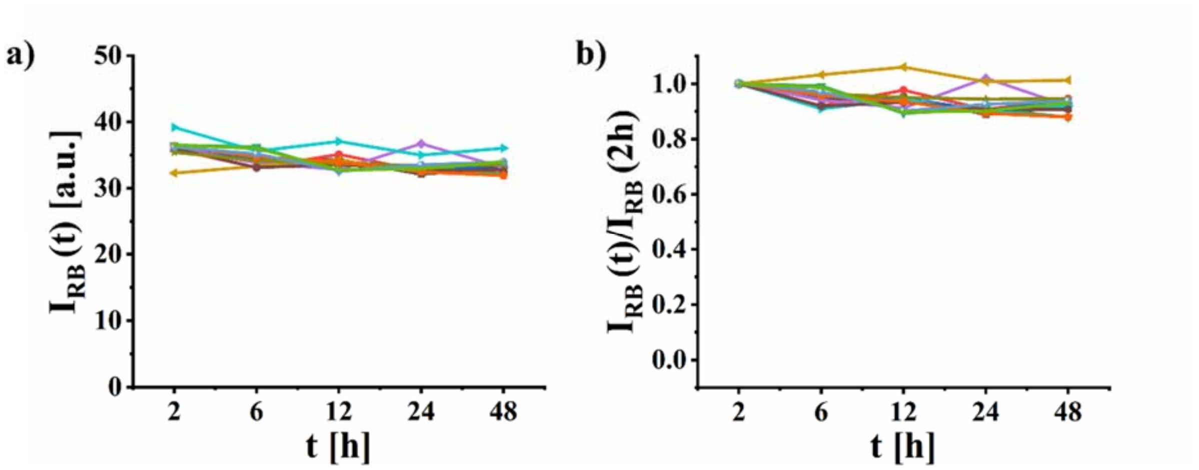

Figure S52. Compilation of all graphs shown in Figure S40c,d - Figure S51c,d. The colors of the curves correspond to the individual curves shown in the respective Figures. Figure b is the same as shown in Figure 5d and is shown also here, to visualize how his Figure has been composed from the data shown in Figures S40 - S51.

8) References

1. Johnson, L. M.; Mecham, J. B.; Krovi, S. A.; Caffaro, M. M. M.; Aravamudhan, S.; Kovach, A. L.; Fennell, T. R.; Mortensen, N. P., Fabrication of polyethylene terephthalate (PET) nanoparticles with fluorescent tracers for studies in mammalian cells. *Nanoscale Advances* **2021,** *3* (2), 339-346.
2. Dhaka, V.; Singh, S.; Anil, A. G.; Naik, T. S. S. K.; Garg, S.; Samuel, J.; Kumar, M.; Ramamurthy, P. C.; Singh, J., Occurrence, toxicity and remediation of polyethylene terephthalate plastics. A review. *Environmental Chemistry Letters* **2022,** *20* (3), 1777–1800.
3. Brkovic, N.; Zhang, L.; Peters, J. N.; Kleine-Doepke, S.; Parak, W. J.; Zhu, D., Quantitative Assessment of Endosomal Escape of Various Endocytosed Polymer-Encapsulated Molecular Cargos upon Photothermal Heating. *SMALL* **2020,** *16* (46), 2003639.
4. Parakhonskiy, B. V.; Parak, W. J.; Volodkin, D.; Skirtach, A. G., Hybrids of Polymeric Capsules, Lipids, and Nanoparticles: Thermodynamics and Temperature Rise at the Nanoscale and Emerging Applications. *Langmuir* **2019,** *35*, 8574–8583.
5. Wang, Y.; Angelatos, A. S.; Caruso, F., Template synthesis of nanostructured materials via layer-by-layer assembly. *Chemistry Of Materials* **2008,** *20* (3), 848-858.
6. Roy, S.; Zhu, D.; Parak, W. J.; Feliu, N., Lysosomal Proton Buffering of Poly(ethylenimine) Measured In Situ by Fluorescent pH-Sensor Microcapsules. *ACS Nano* **2020,** *14*, 8012-8023.
7. Fang, C.; Bhattarai, N.; Sun, C.; Zhang, M., Functionalized nanoparticles with long-term stability in biological media. *Small (Weinheim an der Bergstrasse, Germany)* **2009,** *5* (14), 1637.
8. Moore, T. L.; Rodriguez-Lorenzo, L.; Hirsch, V.; Balog, S.; Urban, D.; Jud, C.; Rothen-Rutishauser, B.; Lattuada, M.; Petri-Fink, A., Nanoparticle colloidal stability in cell culture media and impact on cellular interactions. *Chem Soc Rev* **2015,** *44* (17), 6287-305.
9. del Mercato, L. L.; Abbasi, A. Z.; Parak, W. J., Synthesis and characterization of ratiometric ion-sensitive polyelectrolyte capsules. *Small* **2011,** *7*, 351-363.
10. Zyuzin, M. V.; Díez, P.; Goldsmith, M.; Carregal-Romero, S.; Teodosio, C.; Rejman, J.; Feliu, N.; Escudero, A.; Almendral, M. J. s.; Linne, U.; Peer, D.; Fuentes, M.; Parak, W. J., Comprehensive and Systematic Analysis of the Immunocompatibility of Polyelectrolyte Capsules. *Bioconjugate Chemistry* **2017,** *28*, 556−564.
11. Bradford, M. M., A rapid and sensitive method for the quantitation of microgram quantities of protein utilizing the principle of protein-dye binding. *Analytical Biochemistry* **1976,** *72* (1), 248-254.
12. Ott, A.; Yu, X.; Hartmann, R.; Rejman, J.; Schütz, A.; Ochs, M.; Parak, W. J.; Carregal Romero, S., Light-addressable and degradable silica capsules for delivery of molecular cargo to the cytosol of cells. *Chemistry of Materials* **2015,** *27*, 1929−1942.
13. Chaves, M. R.; Lima, M. L.; Malafatti-Picca, L.; De Angelis, D. A.; Castro, A. M. d.; Valoni, É.; Marsaioli, A. J., A practical fluorescence-based screening protocol for polyethylene terephthalate degrading microorganisms. *Journal of the Brazilian Chemical Society* **2018,** *29* (6), 1278-1285.
14. Pfaff, L.; Breite, D.; Badenhorst, C. P.; Bornscheuer, U. T.; Wei, R., Fluorimetric high-throughput screening method for polyester hydrolase activity using polyethylene terephthalate nanoparticles. In *Methods in enzymology*, Elsevier: 2021; Vol. 648, pp 253-270.
15. Kirchner, C.; Javier, A. M.; Susha, A. S.; Rogach, A. L.; Kreft, O.; Sukhorukov, G. B.; Parak, W. J., Cytotoxicity of nanoparticle-loaded polymer capsules. *Talanta* **2005,** *67*, 486-491.
